# Mak5 and Ebp2 Act Together on Early Pre-60S Particles and Their Reduced Functionality Bypasses the Requirement for the Essential Pre-60S Factor Nsa1

**DOI:** 10.1371/journal.pone.0082741

**Published:** 2013-12-02

**Authors:** Dagmar Pratte, Ujjwala Singh, Guillaume Murat, Dieter Kressler

**Affiliations:** Unit of Biochemistry, Department of Biology, University of Fribourg, Fribourg, Switzerland; Univ. of Edinburgh, United Kingdom

## Abstract

Ribosomes are the molecular machines that translate mRNAs into proteins. The synthesis of ribosomes is therefore a fundamental cellular process and consists in the ordered assembly of 79 ribosomal proteins (r-proteins) and four ribosomal RNAs (rRNAs) into a small 40S and a large 60S ribosomal subunit that form the translating 80S ribosomes. Most of our knowledge concerning this dynamic multi-step process comes from studies with the yeast *Saccharomyces cerevisiae*, which have shown that assembly and maturation of pre-ribosomal particles, as they travel from the nucleolus to the cytoplasm, relies on a multitude (>200) of biogenesis factors. Amongst these are many energy-consuming enzymes, including 19 ATP-dependent RNA helicases and three AAA-ATPases. We have previously shown that the AAA-ATPase Rix7 promotes the release of the essential biogenesis factor Nsa1 from late nucleolar pre-60S particles. Here we show that mutant alleles of genes encoding the DEAD-box RNA helicase Mak5, the C/D-box snoRNP component Nop1 and the rRNA-binding protein Nop4 bypass the requirement for Nsa1. Interestingly, dominant-negative alleles of *RIX7* retain their phenotype in the absence of Nsa1, suggesting that Rix7 may have additional nuclear substrates besides Nsa1. Mak5 is associated with the Nsa1 pre-60S particle and synthetic lethal screens with *mak5* alleles identified the r-protein Rpl14 and the 60S biogenesis factors Ebp2, Nop16 and Rpf1, which are genetically linked amongst each other. We propose that these ’Mak5 cluster’ factors orchestrate the structural arrangement of a eukaryote-specific 60S subunit surface composed of Rpl6, Rpl14 and Rpl16 and rRNA expansion segments ES7L and ES39L. Finally, over-expression of Rix7 negatively affects growth of *mak5* and *ebp2* mutant cells both in the absence and presence of Nsa1, suggesting that Rix7, at least when excessively abundant, may act on structurally defective pre-60S subunits and may subject these to degradation.

## Introduction

The biogenesis of ribosomes is a fundamental cellular process, which provides cells with the molecular machines that translate the genetic information, contained within mRNAs, into proteins. Basically, the biogenesis of ribosomes consists in the ordered assembly of 33 ribosomal proteins (r-proteins) with the 18S ribosomal RNA (rRNA) and 46 r-proteins with the 25S, 5.8S and 5S rRNA into a small 40S and a large 60S ribosomal subunit (r-subunit), respectively, that form upon subunit joining in the cytoplasm the translating 80S ribosomes. Most of our current knowledge concerning this highly dynamic multi-step process comes from studies with the yeast *Saccharomyces cerevisiae*, which have shown that the assembly and maturation of pre-ribosomal particles, as they travel from the nucleolus to the cytoplasm, relies on a multitude (>200) of biogenesis factors [1–3]. Amongst these are, in agreement with the high complexity of this macromolecular assembly process, many energy-consuming enzymes, notably including 19 ATP-dependent RNA helicases and three AAA-type ATPases (ATPases associated with various cellular activities) [2,4–7]. The energy expenditure by these enzymes is thought to be required to trigger irreversible steps of the assembly path and to shape this sophisticated ribonucleoprotein (RNP) complex into its active conformation. Within the nucleolus, the site of rDNA transcription, the emerging precursor rRNA (pre-rRNA) associates with primary binding r-proteins and early biogenesis factors to form the small-subunit (SSU) processome / 90S pre-ribosome [8–10]. Concomitantly, the pre-rRNA associates with 75 small nucleolar RNAs (snoRNAs), with most of these acting, integrated into C/D-box or H/ACA-box snoRNPs, as guides for the modification of pre-rRNA by 2’-O-ribose methylation and pseudouridylation, respectively [11–14]. These modifications, which are concentrated in conserved regions that are functionally important for ribosome function, could help to fine-tune (pre-)rRNA folding, RNP assembly and translation [11,15]. Still within the nucleolar compartment, a subsequent cleavage step, either co- or post-transcriptional, separates the pre-rRNA and generates the 43S and 66S pre-ribosomal particles that upon further maturation eventually give rise to mature 40S and 60S subunits. Pre-40S subunits exhibit a relatively simple composition and are rapidly exported to the cytoplasm where final maturation steps, including 20S pre-rRNA cleavage at site D by the endonuclease Nob1 [16], confer translation competence [17]. In sharp contrast, proteomic approaches revealed several distinct nucleolar and nucleoplasmic pre-60S intermediates that undergo drastic compositional changes [2,18]. Upon arrival of pre-60S subunits in the cytoplasm, the last biogenesis factors are dis- and re-placed by r-proteins in an ordered series of events, thereby enabling subunit joining [3,19].

Due to the complexity of the process and the importance to build ribosomes that translate with high fidelity, it is not surprising that pre-ribosomal particles and mature r-subunits are subjected to quality control [20–22]. The (pre-)rRNAs of defective nuclear pre-ribosomes undergo polyadenylation by the TRAMP complex and are subsequently degraded by the exosome, with both steps occurring in a sub-nucleolar focus termed No-body [20,23–25]. Recent evidence indicates that the D-site cleavage of 20S pre-rRNA, and hence final maturation of pre-40S subunits, occurs within 80S-like ribosomes, thus representing a proofreading step that probes their suitability to engage in translation [26–28]. Likewise, during cytoplasmic maturation of pre-60S subunits, verification of P-site integrity is coupled to the release of the anti-association factor Tif6 [29]. Finally, stalled or functionally defective r-subunits are subjected to ’non-functional rRNA decay’ (NRD) pathways, which eliminate non-functional 40S and 60S r-subunits *via* degradation of the rRNA or targeting to the proteasome, respectively [22,30–33].

Most of the DExD/H-box RNA helicases involved in ribosome biogenesis act at early steps of ribosome assembly within the nucleolus where extensive structural rearrangements of (pre-)-rRNA and incorporation of the majority of r-proteins are expected to occur [2,6]. Considering the reported functional and enzymatic properties, DExD/H-box RNA helicases can be generally viewed as energy-consuming chaperones or modulators of RNA and RNP structures [34–36]. In most cases, however, we are far from understanding their exact molecular functions during ribosome assembly (reviewed in [2,5,6]); it will therefore be an enormous future challenge to obtain insight into their pre-rRNA binding sites and to unravel the regulation and timing of their enzymatic activity. On the other hand, GTPases, kinases and the three AAA-type ATPases act predominantly at later stages during ribosome biogenesis [2].

Contrary to the proposed pre-rRNA or RNP remodelling functions of most DExD/H-box RNA helicases involved in ribosome biogenesis, recent evidence revealed that the three AAA-ATPases are required for the release of distinct substrate proteins from nucleolar, nucleoplasmic and cytoplasmic pre-60S intermediates [37–40]. The type II AAA-ATPases Rix7 and Drg1, which are closely related to the well-known Cdc48 (p97 in mammals) whose diverse functions are largely linked to the recognition of ubiquitinated substrate proteins [41,42], mediate the release of the essential biogenesis factors Nsa1 and Rlp24 from nucleolar or cytoplasmic pre-60S particles, respectively [38,39]. Despite their striking similarity to Cdc48/p97 [4], there is so far no evidence that links Rix7 or Drg1 to the degradation of defective pre-60S ribosomes or parts thereof. On the other hand, Rea1, the largest yeast protein, shares similarity with the dynein heavy chain protein Dyn1 [4]. Rea1 is involved in two successive pre-60S maturation steps and promotes the release of distinct biogenesis modules (Ytm1-Erb1-Nop7 / Rsa4, Rix1-Ipi3-Ipi1), thus mediating nucleolar exit and priming pre-60S subunits for nuclear export [37,40]. In all cases, the removal of these biogenesis factors from pre-60S particles is essential for their recycling and, importantly, triggers major compositional or conformational changes that constitute key maturation events necessary for the progression of ribosome assembly.

We are ultimately interested in understanding the mechanistic details of the Rix7-mediated release of Nsa1 from late nucleolar pre-60S particles and how the entailed structural rearrangements reshape pre-60S intermediates and allow progression of ribosome assembly. Here we reveal that the lethality associated with the absence of Nsa1 can be suppressed by mutant alleles of the biogenesis factors Ebp2, Mak5, Nop1 and Nop4, thus genetically unravelling possible structural changes within pre-60S particles that influence their assembly kinetics and/or path in order to compensate for the lack of Nsa1 recruitment. Moreover, genetic experiments indicate that Rix7 may have other nuclear substrates besides Nsa1 and that it may recognize and target defective pre-60S subunits for degradation. Finally, our analysis of the functional network around the DEAD-box RNA helicase Mak5 suggests that the ’Mak5 cluster’ factors (Mak5, Ebp2, Nop16 and Rpf1) may orchestrate the structural arrangement of a eukaryote-specific 60S subunit surface composed of Rpl6, Rpl14 and Rpl16 and rRNA expansion segments ES7L and ES39L.

## Materials and Methods

### Yeast strains and genetic methods

The S. *cerevisiae* strains used in this study are listed in Table S1, all strains are derivatives of W303 [43] or DS1-2b [44]. For yeast two-hybrid analyses the reporter strain PJ69-4A was used [45]. Deletion disruption and C-terminal tagging at the genomic locus were performed as described [46–48]. Preparation of media, yeast transformation, and genetic manipulations were done according to established procedures.

### Plasmid constructs

All recombinant DNA techniques were according to established procedures using *Escherichia coli* DH5α for cloning and plasmid propagation. All cloned DNA fragments generated by PCR amplification were verified by sequencing. Human NSA1 was PCR-amplified from a cDNA library and first cloned into pBSKS(-) (Stratagene) and then, after a fusion PCR to eliminate mutations within the hNSA1 gene, cloned under the control of the *ADH1* promoter in a YCplac111-based vector. More information on the plasmids, which are listed in Table S2, is available upon request.

### Isolation and cloning of spontaneous *∆nsa1* bypass suppressors

The *NSA1* shuffle strain Y3900 (see Table S1) was transformed with pADH111-hNSA1.N328 and restreaked or spotted, after selection of cells having lost the *NSA1* shuffle plasmid pHT4467∆-*NSA1* (*URA3*-*ADE3* ) on 5-FoA-containing plates, on YPD plates. The gene complementing the suppressor mutation S5 was cloned by transformation of strain YHD60, which is a meiotic segregant of a cross between suppressor strain S5 and Y3274, with a YCplac111-based yeast genomic library [49]. The library plasmid was rescued from one transformant that showed complementation of the slow-growth phenotype but did not grow on 5-FoA-containing plates. The gene complementing the temperature-sensitive (ts) phenotype associated with the suppressor mutation S4 was cloned by transformation of a strain originating from a backcross of suppressor strain S4 with the *NSA1* shuffle strain Y3903 with a yeast genomic library in YCplac111. To clone the gene complementing the ts phenotype of the suppressor mutant Sj, the suppressor strain Sj was first transformed with YCplac33-*NSA1* and then with the YCplac111-based yeast genomic library. To ascertain that the suppressor strains carried indeed mutations in the genes complementing the growth and suppression phenotypes, *mak5-S5*, *nop4-S4* and *nop1-Sj* were amplified by PCR from genomic DNA of the respective suppressor strains. Upon cloning of the PCR products, the nature of the mutation within the suppressor alleles was identified by sequencing.

### Synthetic lethal screens

Synthetic lethal (sl) screens with the *mak5.G218D* and mak5.R728* alleles were essentially performed as previously described [49]. The above *mak5* alleles were cloned into YCplac22, and the resulting plasmids were transformed into the *MAK5* shuffle strains YDP1 and YDP4, which bear *MAK5* on the instable *URA3 ADE3* marker containing plasmids pHT4467∆. Cells were grown to exponential phase in liquid SC-Ura-Trp medium, plated on SC-Trp plates and then mutagenized by irradiation with UV light in a Stratalinker 1800 (Stratagene). Cloning of the genes complementing the respective sl-mutations was achieved by using a yeast genomic library in YCplac111 or YEplac181 [49,50]. The sl-screen with mak5.R728* yielded the following candidates whose complementing genes could be cloned: six times *NOP16* (SL2, SL4, SL63, SL67, SL85 and SL113), two times *RPF1* (SL3 and SL33) and one time *RPL14A* (SL103). The sl-screen with *mak5.G218D* yielded only one sl-candidate for which it was possible to clone the complementing gene: *EBP2* (SL19). To ascertain that the genes cloned by complementation indeed harboured mutations in the sl-mutant strains, genomic DNA was prepared from the sl-strains and the corresponding mutant alleles were amplified by PCR and directly sequenced and/or cloned into suitable plasmids and then sequenced.

### Sucrose gradient analysis and fractionation

Cell extracts for polysome profile analyses were prepared as previously described [51] and layered onto 10-50% sucrose gradients that were centrifuged at 38’000 rpm in a Sorvall TH-641 rotor at 4°C for 2 h 30 min or 2 h 45 min. Sucrose gradients were analysed and fractionated using an ISCO UA-6 system with continuous monitoring at A_254_.

### Preparation of total yeast protein extracts and Western analysis

Total yeast protein extracts were prepared as previously described [52]. Cultures were grown to an OD_600_ of around 0.8 and protein extracts were prepared from an equivalent of one OD_600_ of cells. Western blot analysis was carried out according to standard protocols. The following primary antibodies were used in this study: mouse monoclonal anti-GFP (1:3’000; Roche) and anti-Rpl3 (1:5’000; J. Warner, Albert Einstein College of Medicine, New York, NY); rabbit polyclonal anti-CBP (1:15’000; Open Biosystems), anti-Arc1 (1:20’000; [53]), anti-Ebp2 (1:10’000; J. Woolford, Carnegie Mellon University, Pittsburgh, PA), and anti-Nop7 (1:40’000; B. Stillman, Cold Spring Harbour Laboratory, Cold Spring Harbour, NY). Secondary goat anti-rabbit (Bio-Rad) or anti-mouse (Bio-Rad) horseradish peroxidase-conjugated antibodies were used at a dilution of 1:10’000. For detection of TAP-tagged proteins the Peroxidase-Anti-Peroxidase soluble complex was used at a dilution of 1:15’000 (Sigma). Proteins were visualized using enhanced chemiluminescence detection kits (Immobilon Western, Millipore; Amersham ECL, GE Healthcare).

### Tandem affinity purification (TAP)

TAP purifications of TAP-tagged bait proteins were performed in a buffer containing 50 mM Tris-HCl pH 7.5, 100 mM NaCl, 1.5 mM MgCl_2_, 5% glycerol and 0.1% NP-40 essentially as described previously [44,48]. For TEV cleavage, DTT was added to a final concentration of 1 mM to the above buffer. Elution from Calmodulin-Sepharose beads was performed in the presence of 5 mM EGTA. The EGTA eluates were precipitated by the addition of TCA to a final concentration of 10% and dissolved in SDS sample buffer. The samples were then separated on NuPAGE SDS 4-12% gradient polyacrylamide gels (Invitrogen) and stained with colloidal Coomassie (Sigma) or analyzed by Western blotting.

### Sequence alignments, secondary structure prediction and analysis of 3D structures

Multiple sequence alignments of orthologous proteins were generated in the ClustalW output format with T-Coffee using the default settings of the EBI website interface [54]. Secondary structure prediction was performed with the PSIPRED v3.3 prediction method available at the PSIPRED website interface [55]. Analysis and image preparation of three-dimensional structures, downloaded from the PDB archive, was carried out with the PyMOL software (PyMOL Molecular Graphics System). For analysis of the eukaryote-specific 60S subunit surface surrounding Rpl14 (L14e), we used the coordinates for 60S r-subunits from the recent *S. cerevisiae* 80S crystal structure (PDB 3U5H and 3U5I; [56]) and human 80S cryo-EM structure (PDB 3J3B and 3J3F; [57]).

## Results

### The essential Nsa1 function can be bypassed by mutation of *MAK5*, *NOP1* and *NOP4*

We have previously shown that the AAA-type ATPase Rix7 strips the essential biogenesis factor Nsa1 from a distinct nucleolar pre-60S particle (see Introduction and [39]). To address whether Nsa1 fulfils an evolutionarily conserved function in ribosome biogenesis, we tested if human NSA1 complemented the lethality of the *∆nsa1* null mutant. Nsa1 is predicted to form an N-terminal 7-bladed WD β-propeller followed by a non-essential C-terminal extension (D.K., unpublished data). As shown in Figure S1, human NSA1 lacking the C-terminal extension (hNSA1.N328) was capable of conferring weak growth to *∆nsa1* null mutant cells (Figure S1). Interestingly, we observed that faster growing colonies appeared spontaneously at a rather high frequency (Figure S1), indicating that the dysfunctionality or even absence of Nsa1 can be easily suppressed. To facilitate cloning of the genes complementing the suppressor mutations, we selected in a first instance those suppressor strains where the suppressor mutation conferred a clear slow-growth or temperature-sensitive (ts) phenotype. By this approach, we managed to clone the genes complementing the suppressor mutation of three suppressor strains (see Materials and Methods). Subsequent PCR amplification and sequencing of the suppressor alleles revealed that all three strains indeed contained mutations in the genes that we had cloned by complementation. Suppressor strain S5 harbours a mutation in *MAK5* [mak5.R728*; Arg728(AGA) to stop (TGA)], which encodes a nucleolar DEAD-box RNA helicase involved in 60S subunit biogenesis [58]. Suppressor strain S4 contains a mutation in *NOP4* [*nop4.S460L*; Ser460(TCG) to Leu(TTG)], encoding a nucleolar protein with four RRM motifs that binds to the pre-rRNA in proximity of the 5’-end of the 5.8S rRNA and is required for the stable formation of pre-60S subunits containing the 27SA and 27SB pre-rRNAs [59–61]. Suppressor strain Sj comprises a mutation in *NOP1* [*nop1.M232K*; Met232(ATG) to Lys(AAG)], coding for the methyltransferase component of C/D-box snoRNPs [62–66]. We first assessed the growth phenotypes of these three mutant alleles when expressed under the control of their cognate promoters from a centromeric plasmid (Figure S2A-C). While the mak5.R728* mutant shows a slow-growth phenotype at all temperatures, especially at 37°C, the *nop4.S460L* and *nop1.M232K* mutants display only a very mild growth defect at 30°C and are ts at 37°C. Polysome profile analyses revealed that all three mutations confer a deficiency in 60S subunit production, as evidenced by the reduction in free 60S subunits and the occurrence of half-mer polysomes (Figure S2A-C). Since Nop4 was originally identified in a synthetic lethal (sl) screen with the *nop1-5* allele [59], we tested whether the *nop1.M232K* and *nop4.S460L* alleles are also genetically linked. As shown in Figure S3, combination of these two mutations resulted in a synergistic slow-growth phenotype (Figure S3).

**Figure 1:**
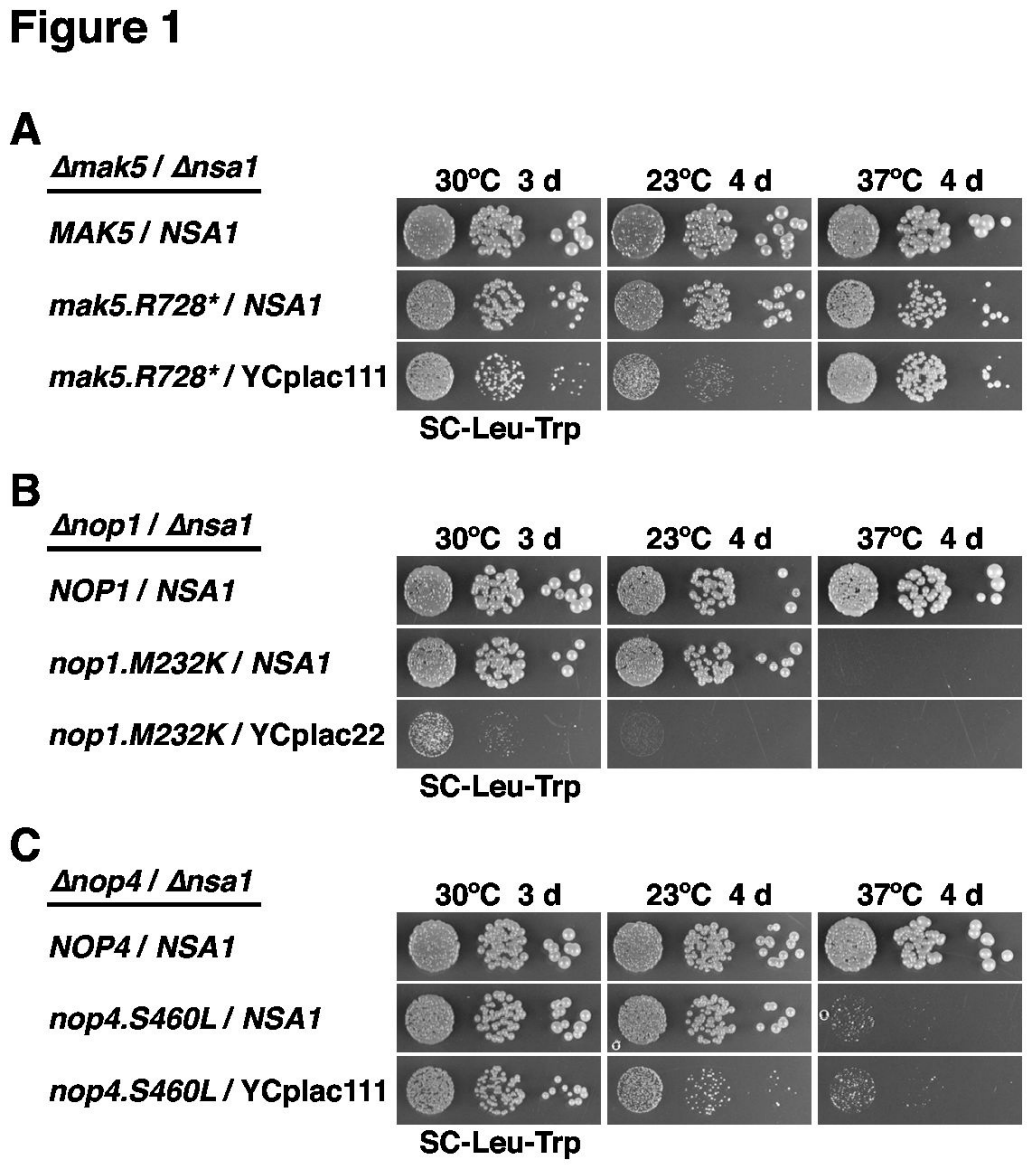
Bypass suppression of *∆nsa1* null lethality by the mak5.R728*, *nop1.M232K* and *nop4.S460L* alleles. *MAK5*/*NSA1* (**A**), *NOP1*/*NSA1* (**B**) and *NOP4*/*NSA1* (**C**) double shuffle strains were co-transformed with plasmids harbouring the indicated wild-type and mutant alleles and/or empty vectors (YCplac111 or YCplac22). After plasmid shuffling on plates containing 5-FoA, cells were restreaked on synthetic complete medium lacking leucine and tryptophan (SC-Leu-Trp) and then spotted in 10-fold serial dilution steps onto SC-Leu-Trp plates, which were incubated for 3 d at 30°C, 4 d at 23°C and 4 d at 37°C.

We next determined whether the isolated *mak5*, *nop1* and *nop4* alleles, when expressed from a centromeric plasmid, had the capacity to suppress the absence of Nsa1 in the setting of clean double shuffle strains. All three alleles bypassed the requirement for Nsa1, as revealed by the growth of *∆nsa1* null mutant cells expressing the mutant variants of Mak5, Nop1 and Nop4 on plates containing 5-fluoroorotic acid (5-FoA) (Figure S4). To better assess the extent of suppression, we compared the growth properties of cells harbouring the suppressor alleles in the presence or absence of *NSA1* after plasmid shuffling (Figure 1A-C). This growth analysis showed that the *nop4.S460L* allele, even though conferring only a very mild growth defect on its own, very efficiently suppressed the lethality of the *∆nsa1* null mutant (Figure 1C), while the mak5.R728* and *nop1.M232K* alleles exhibited a moderate and weak extent of suppression, respectively (Figure 1A and B). Notably, suppression was for all three alleles more efficient at 30°C than at 23°C; moreover, growth of the mak5.R728* mutant was identical in the presence or absence of Nsa1 at 37°C (Figure 1A-C). We conclude that the absence of the essential, pre-60S associated Nsa1 can be relatively easily bypassed by mutation of genes encoding biogenesis factors that act at different nucleolar stages of pre-60S maturation (see Discussion and also below). Since we did not carry out an in-depth suppressor screen, it is very likely that reduced functionality of other protein trans-acting factors, besides Mak5, Nop1 and Nop4, may also lead to bypass suppression of the lethal *∆nsa1* null phenotype.

### Rix7 may have additional nuclear functions besides stripping Nsa1 from pre-60S particles

**Figure 2:**
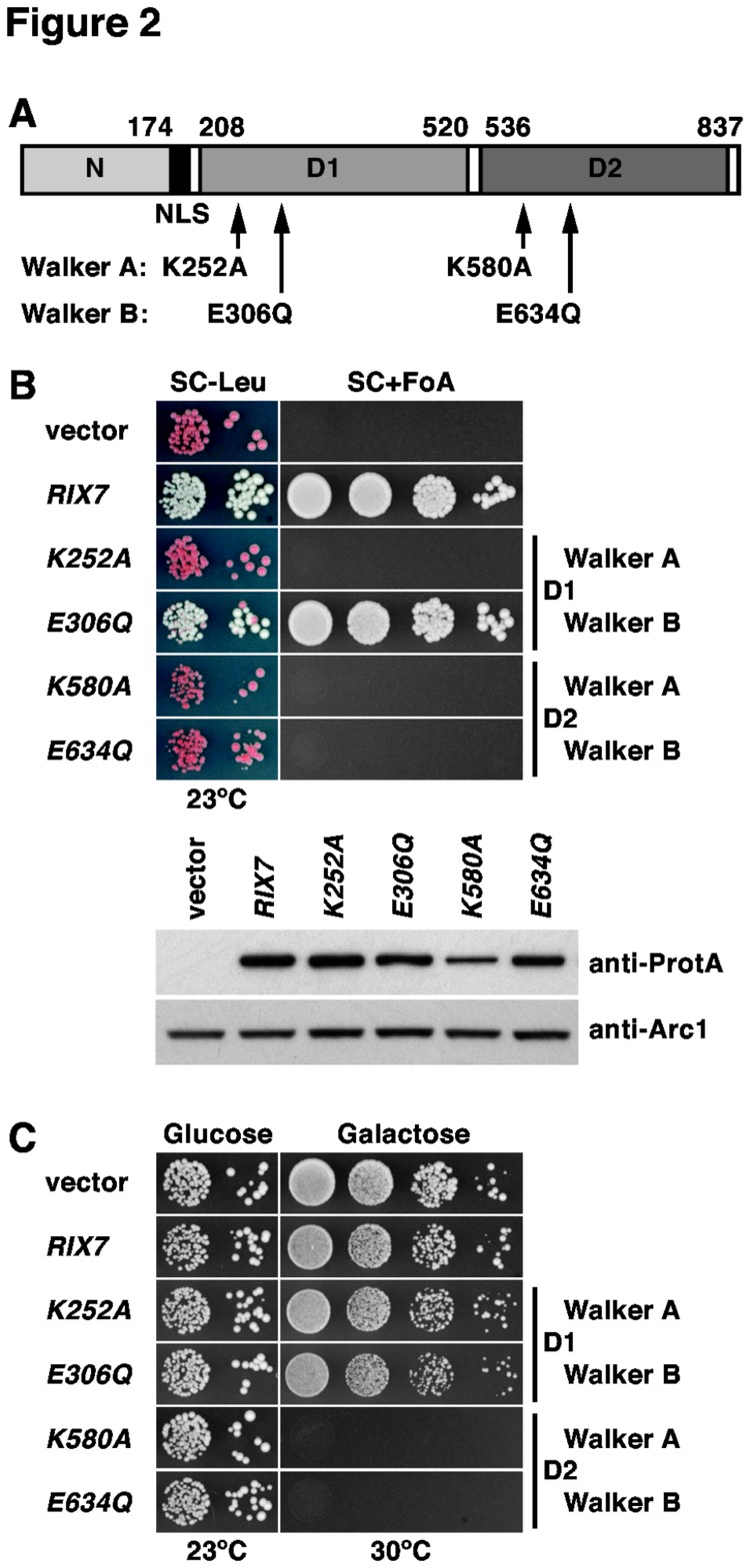
The D2 domain of the AAA-ATPase Rix7 harbors the catalytic activity. (**A**) Schematic representation of the domain organization of Rix7. N, N-terminal domain (amino acids 1-174); NLS, predicted bipartite nuclear localization signal (amino acids 175-194); D1, AAA-domain D1 (amino acids 208-520); D2, AAA-domain D2 (amino acids 536-823). The amino acid changes within the Walker A and B motifs of D1 or D2, respectively, are indicated. (**B**) *In* *vivo* phenotypes of the Walker A and B mutants generated in D1 and D2. Empty vector (YCplac111) and plasmid-borne wild-type RIX7 or the indicated *rix7* mutant alleles under the control of the authentic promoter were transformed into the RIX7 shuffle strain. Transformants were spotted in 10-fold serial dilution steps onto SC-Leu and SC+5-FoA plates, which were incubated for 5 d at 23°C (upper panel). Expression levels of plasmid-borne TAP-tagged wild-type RIX7 and the indicated *rix7* Walker A and B mutant alleles, expressed from their cognate promoter in a haploid wild-type strain, were determined in whole cell lysates by Western analysis using anti-ProtA and anti-Arc1 (loading control) antibodies (lower panel). (**C**) Walker A and B mutations within AAA-domain D2 confer a dominant-lethal phenotype. Empty vector and wild-type RIX7 or the indicated *rix7* mutant alleles, expressed under the control of the inducible *GAL1-10* promoter, were transformed into a haploid wild-type strain. Transformants were spotted in 10-fold serial dilution steps onto SC-Leu (Glucose) and SGal-Leu (Galactose) plates, which were incubated for 4 d at 23°C and 30°C, respectively.

The availability of bypass suppressors of the lethal *∆nsa1* null mutant put us in the unique situation to address whether the AAA-ATPase Rix7 may have additional substrates besides Nsa1 by testing the dominant-negative effect of *RIX7* alleles in the ’suppressor’ strains. However, a pre-requisite for this analysis was the construction of dominant-negative alleles of *RIX7*. Rix7 exhibits the typical domain organization of type II AAA-ATPases, notably containing two central AAA-domains (termed D1 and D2) flanked by a long N-terminal and a very short C-terminal extension (Figure 2A; [4,39]). To define whether ATP binding and ATPase activity are essential features of both D1 and D2 by *in vivo* experiments, we mutated conserved residues that are implicated in ATP binding (Walker A motif / P-loop) and ATP hydrolysis (Walker B motif / DExx-box) [67], then we assessed the complementation of the lethal *∆rix7* null mutant phenotype by these constructs. This analysis revealed that Rix7 variants with a mutated Walker A motif within AAA-domain D1 (K252A) or mutated Walker A or B motifs within D2 (K580A and E634Q, respectively) are non-functional, while mutation of the Walker B motif within D1 (E306Q) has no significant effect on cell growth (Figure 2B). In conclusion, adenosine nucleotide binding (ATP or ADP) is necessary whereas ATP hydrolysis seems to be dispensable for AAA-domain D1 function; however, AAA-domain D2 function requires both ATP binding and hydrolysis. In agreement with this interpretation, both D2 mutants, but not the D1 Walker A mutant, conferred a dominant-negative phenotype when over-expressed from a galactose-inducible promoter in a wild-type background (Figure 2C). Similarly to Rix7, the main catalytic activity of the closely related AAA-ATPases Cdc48 and Drg1 also resides in their D2 domains [38,68,69].

**Figure 3:**
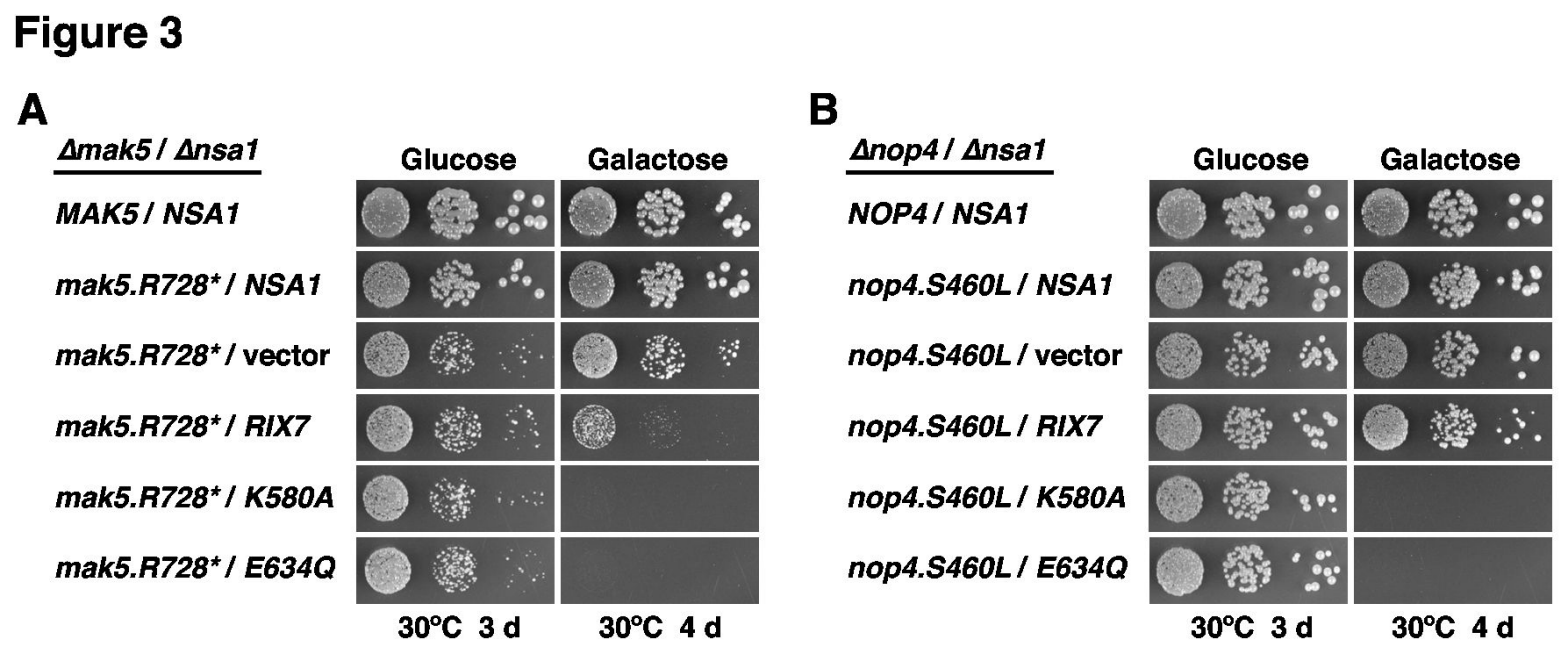
Dominant-lethal *RIX7* alleles retain their negative effect on growth in the absence of Nsa1. *MAK5*/*NSA1* (**A**) and *NOP4*/*NSA1* (**B**) double shuffle strains were co-transformed with plasmids harbouring either the MAK5 or NOP4 wild-type genes or the mak5.R728* or *nop4.S460L* mutant alleles and plasmids carrying the NSA1 wild-type gene or, expressed under the control of the *GAL1-10* promoter, wild-type RIX7 and the dominant-negative *RIX7.K580A* or *RIX7.E634Q* alleles or empty vector. After plasmid shuffling on plates containing 5-FoA, cells were restreaked on SC-Leu-Trp plates and then spotted in 10-fold serial dilution steps onto SC-Leu-Trp (Glucose) and SGal-Leu-Trp (Galactose) plates, which were incubated for 3 d or 4 d at 30°C, respectively.

Having dominant-negative alleles of *RIX7* at hand, we next assessed their effect on the growth of the mak5.R728* and *nop4.S460L ∆nsa1* null ’suppressor’ strains. Interestingly, galactose-induced over-expression of the dominant-negative *RIX7.K580A* and *E634Q* mutants still resulted in a lethal phenotype (Figure 3A and B); thus, suggesting that Rix7 may have additional nuclear substrates besides Nsa1. Intriguingly, we noticed that over-expression of wild-type *RIX7* already had a strong negative effect on the growth of mak5.R728* cells lacking Nsa1 (Figure 3A) – this effect was less pronounced when the *∆nsa1* null mutation was suppressed by *nop4.S460L* (Figure 3B). This observation indicates that Rix7, in order to exert such a negative effect on growth, is capable of recognizing pre-60S particles that lack Nsa1. However, since no negative effect of Rix7 over-expression was observed in a wild-type background (Figure 2C), Rix7 presumably only acts on damaged pre-60S particles, which likely manifest their structural deficiency in a characteristic manner (see below and Discussion).

### Mak5 is associated with the Nsa1 pre-60S particle

**Figure 4:**
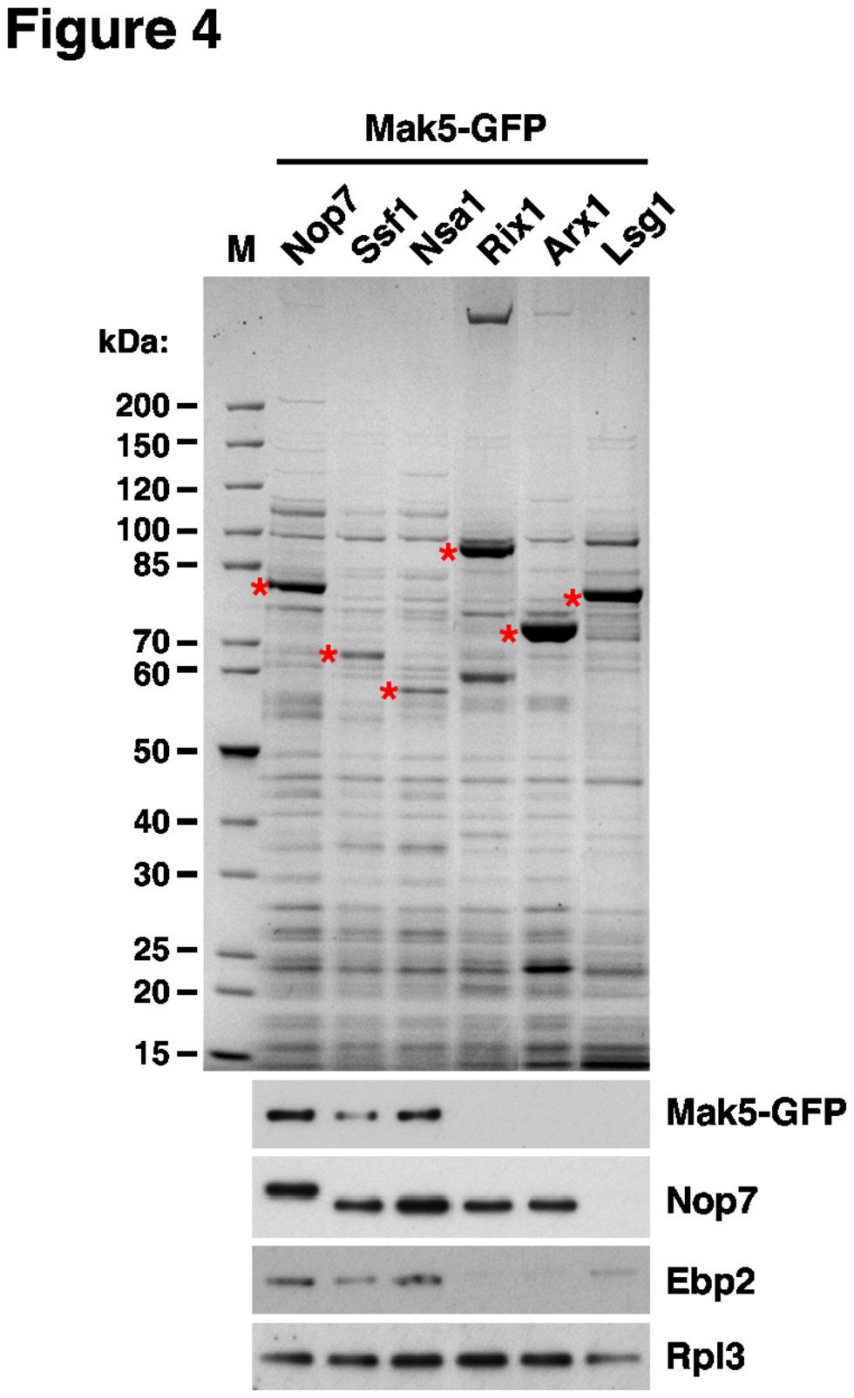
The DEAD-box protein Mak5 is associated with the Nsa1 pre-60S particle. Mak5 is associated with the early nucleolar Ssf1 and late nucleolar Nsa1 pre-60S particles. The indicated TAP-tagged bait proteins were affinity-purified from cells expressing Mak5-GFP. The final EGTA eluates were analyzed by SDS-PAGE and Coomassie staining (top) and Western blotting using anti-GFP, anti-Nop7, anti-Ebp2 and anti-Rpl3 antibodies (bottom).

Since we are ultimately interested in understanding how Rix7 strips Nsa1 from late nucleolar pre-60S particles, we did not consider to further investigate the basis of the suppression of *∆nsa1* by the *nop1.M232K* and *nop4.S460L* allele in the framework of this study, because these two factors act substantially upstream of the Nsa1 pre-60S particle: (i) Nop1, as the methyltransferase component of C/D-box snoRNPs [62–66], acts primarily on 90S pre-ribosomes and very early pre-60S particles and (ii) Nop4 is mainly associated with very early pre-60S particles [8,9,39,70–73]. On the other hand, the reduced formation of mature 25S and 5.8S rRNA from 27S pre-rRNAs upon genetic depletion of Mak5 suggested an involvement of Mak5 in a late step during the nucleolar phase of pre-60S maturation [58]. To address whether Mak5 is specifically associated with nucleolar pre-60S particles, we purified, by applying the tandem-affinity purification (TAP) method, several distinct pre-60S particles from cells expressing chromosomally integrated Mak5-GFP (Figure 4). The TAP-tagged bait proteins subjected to the purifications notably define early nucleolar (Ssf1-TAP), late nucleolar (Nsa1-TAP), nucleoplasmic (Rix1-TAP), nucleoplasmic/cytoplasmic (Arx1-TAP) and late cytoplasmic (Lsg1-TAP) pre-60S particles, while the Nop7-TAP bait purifies a mixture of very early to late nuclear pre-60S particles [19,39,44,74]. As revealed by Western analysis using anti-GFP antibodies, Mak5 is exclusively associated with nucleolar pre-60S particles purified by the Ssf1 and Nsa1 baits (Figure 4). Since Mak5 was previously neither found as a stoichiometric component of Ssf1- nor Nsa1-defined pre-60S particles [39,74], we conclude that Mak5 is only transiently or weakly associated with pre-60S ribosomes.

### The C-terminal extension to the DEAD-box core is important for Mak5 function

Mak5 contains N- and C-terminal extensions of around 170 amino acids flanking the central DEAD-box core (Figures S5A and S5B). The identification of mak5.R728* as a *∆nsa1* suppressor allele suggested that the C-terminal extension is important for Mak5 function. Sequence comparison revealed that the C-terminal extension is relatively well conserved both in primary sequence and secondary structure, notably predicted to harbour four conserved α-helices (Figure S5A). On the other hand, the N-terminal extension contains only few conserved regions and there is no clear conservation of the predicted secondary structure elements (data not shown). To better define the contribution of the N- and C-terminal extension to the biological function of Mak5, we undertook a detailed deletion analysis (Figures S5B and S5C). While a Mak5 variant lacking almost the complete C-terminal extension (*mak5.N629*; amino acids 1-629) did not support growth (Figure S5B), the N-terminal deletion construct of Mak5 (*mak5.166C*; amino acids 166-773) exhibited a moderate slow-growth phenotype at 30°C and a pronounced growth defect at 37°C (Figures S5B and S5C). Progressive C-terminal deletion mapping revealed that already a construct expressing amino acids 1-673 of Mak5 (*mak5.N673*) was not able to confer growth and that the first viable mutant harboured a C-terminal deletion of 50 amino acids (*mak5.N723*) (Figure S5B). Next, we assessed the growth properties of the viable N- and C-terminal deletion mutants after plasmid shuffling on YPD plates at different temperatures (Figure S5C), revealing in both cases that the growth defects, which were not due to decreased expression levels (data not shown), were more pronounced at 37°C. Finally, polysome profile analysis showed a shortage of 60S subunits both for mutants lacking the complete N-terminal extension (*mak5.166C*) or the C-terminal 50 amino acids (*mak5.N723*) (Figure S6). Altogether, we conclude that the C-terminal extension of Mak5, in agreement with its higher degree of evolutionary conservation, is functionally more relevant than the N-terminal extension. Moreover, due to its essential nature, we predict that the C-terminal extension may mediate recognition of the cognate RNA or RNA:protein substrate on pre-60S particles.

### Mak5 functionally interacts with Ebp2, Nop16, Rpf1 and Rpl14

Since DExD/H-box RNA helicases largely rely on a specific RNA substrate and/or protein co-factors to efficiently carry out their biological functions (see for example [75–81]), we set out to perform a sl-screen with different *mak5* alleles in order to establish the functional network around Mak5 and, if possible, to identify the protein partner(s) that may be required for stimulation of Mak5’s ATPase activity and/or for its recruitment to the cognate substrate. To maximize an optimal recovery of a broad range of distinct sl-partners, we chose to use *mak5* alleles harbouring mutations within motif I (Walker A motif; involved in ATP binding) or the C-terminal extension, which are therefore likely affecting ATPase activity (*mak5.G218D*) or substrate recognition (mak5.R728*). To validate the suitability of the *mak5.G218D* allele [58], we determined its growth characteristic and polysome profile, revealing a moderate growth defect 23°C, 30°C and 37°C and a deficiency in production of 60S subunits (Figures S5C and S6). The sl-screen with the mak5.R728* allele yielded nine sl-mutants for which we managed to clone the complementing genes, with six of these being complemented by *NOP16*, two by *RPF1* and one by *RPL14A* (see also Materials and Methods). Of the obtained sl-candidates from the *mak5.G218D* screen we could complement one sl-mutant with *EBP2*. In agreement with a function of Mak5 during the nucleolar phase of pre-60S maturation, all the proteins encoded by the identified genes are known to have a role in 60S biogenesis and/or are associated with the Nsa1-defined pre-60S particle or, as for the r-protein Rpl14a, to be a constituent of mature 60S subunits [39,56,73,82–85].

As a next step, we confirmed that the sl-mutant strains indeed contained genomic mutations within the genes that were cloned by complementation. To this end, we PCR amplified and either sequenced directly the PCR products or first cloned and then sequenced the inserts of the plasmids containing the mutant alleles. To visualize the mutations that are present in the individual sl-alleles of the four different genes, we have highlighted the entailed amino acid changes within multiple sequence alignments of the orthologous proteins from *S. cerevisiae*, *Schizosaccharomyces pombe* and *Homo sapiens* (Figures S7A, S8A, S9A, and S10A). To assess the phenotypic consequences of these mutations on growth and 60S subunit biogenesis, we either expressed the mutant alleles from centromeric plasmids under the control of their cognate promoters in the respective shuffle strains (for the *ebp2*, *rpf1* and *rpl14a* alleles) or generated a *∆nop16* null mutant. The ebp2.K287* allele introduces a stop codon after amino acid 286, thereby truncating Ebp2 at the beginning of a predicted, ~60 amino acid long α-helix within the highly conserved Ebp2-core domain (Figures S7A and S14), and confers a relatively strong slow-growth phenotype, which is exacerbated at 37°C, that coincides with a clear reduction in free 60S subunits, the accumulation of half-mer polysomes and an overall reduction in polysome content (Figure S7B). The two *rpf1* alleles, *rpf1.L199P* and *rpf1.C139R*, similarly affect growth (mild slow growth at 30°C and almost ts at 37°C) and 60S subunit production (Figure S8B). Notably, Leucine 199 is within a highly conserved stretch of Rpf1 and precedes the C-terminally located σ^70^-like RNA-binding motif by ~55 amino acids (Figure S8A and [85]). The mutations within the six *NOP16*-complemented sl-mutants represent five different alleles of *nop16*, with all of these, due to the introduction of frameshifts and/or pre-mature stop codons, likely completely abrogating Nop16 function (Figure S9A). We therefore assessed the growth and polysome profile phenotype of *∆nop16* null mutant cells, revealing a very modest growth defect and a mild reduction in free 60S subunits that was notably visible by the appearance of half-mer polysomes (Figure S9B). We note that Nop16 – despite the very mild impact of the absence of Nop16 on yeast growth under optimal laboratory conditions – is still present in higher eukaryotic and even mammalian organisms (Figure S9A). The essential r-protein Rpl14 (L14e according to the newly proposed nomenclature for ribosomal proteins [86]), which is conserved in some but not all archaeal organisms [56,87], is encoded in *S. cerevisiae* by the duplicated genes *RPL14A* and *RPL14B*. The rpl14a.L123* allele, by changing the triplet coding for Leucine 123 to a stop codon, encodes a mutant Rpl14a protein with a truncated C-terminal α-helix (Figure S10A), which notably mediates interactions with the eukaryote-specific C-terminal α-helix of Rpl16 (L13), the C-terminal region of the eukaryote-specific ribosomal protein Rpl6 (L6e), and the eukaryote-specific rRNA expansion segment ES39L (see Figures S10B, S10C and S11B; [56]). Interestingly, the two C-terminal α-helices of eukaryotic Rpl14 are absent from the existing archaeal L14 orthologues (Figure S11A). The rpl14a.L123* mutant variant, when expressed under the control of its cognate promoter from a centromeric plasmid in *∆rpl14a* null mutant cells, confers a mild growth defect that is exacerbated a lower temperatures (Figure S12A). In agreement with an effect on the biogenesis of 60S subunits, the rpl14.L123* mutant displayed a slight decrease in free 60S subunits and some accumulation of half-mer polysomes (Figure S12A). However, when supplied as the sole Rpl14 copy in a *RPL14* shuffle strain (*∆rpl14a*/*∆rpl14b*), the rpl14a.L123* allele elicited a more pronounced growth and 60S subunit biogenesis defect (Figure S12B). Notably, growth of rpl14a.L123* cells was particularly affected at 37°C and 18°C (Figure S12B). Nevertheless, we still observed even stronger growth and 60S subunit deficits in *∆rpl14a* cells (Figure S12A). On the other hand, growth and 60S subunit deficiency of *∆rpl14b* cells was very similar to the one of rpl14a.L123*/RPL14B cells (Figures S12A and S12C). It can therefore be concluded that, even though *RPL14A* and *RPL14B* encode identical proteins (e.g. in our W303 background), the contribution of Rpl14a to the functional pool of Rpl14 is more prominent than the one of Rpl14b (see also [88]).

### Mak5, Ebp2, Nop16, Rpf1 and Rpl14 form a genetically defined functional cluster

**Figure 5:**
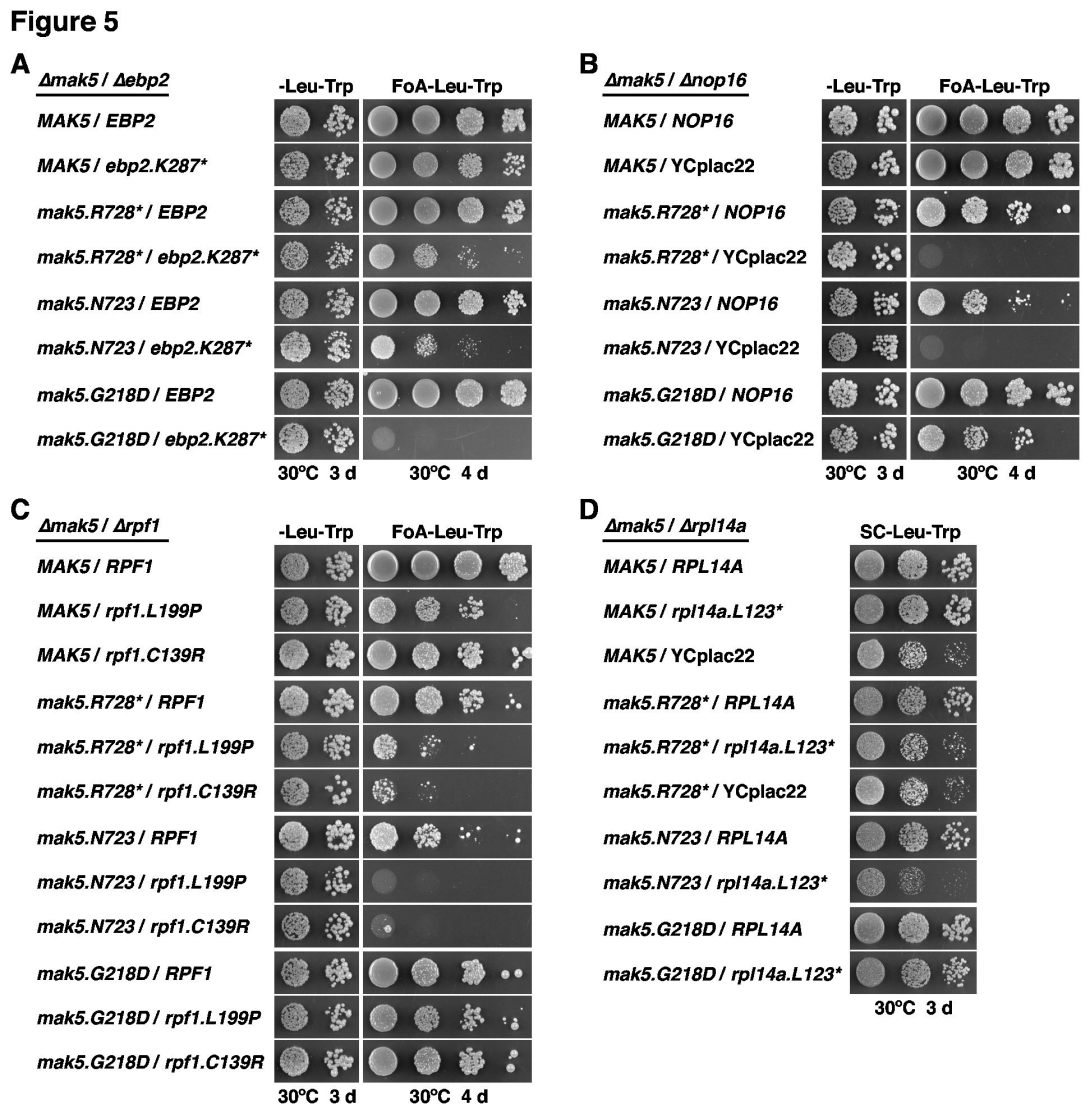
Synthetic lethal interactions between different *mak5* alleles and *ebp2*, *∆nop16*, *rpf1* and *rpl14a* alleles. *MAK5*/*EBP2* (**A**), *MAK5*/*∆nop16* (**B**), *MAK5*/*RPF1* (**C**) and *MAK5*/*RPL14A* (**D**) shuffle or double shuffle strains were co-transformed with plasmids harbouring the indicated wild-type and mutant alleles and/or empty vector (YCplac22). Cells were restreaked on SC-Leu-Trp plates and then spotted in 10-fold serial dilution steps onto SC-Leu-Trp and SC+5-FoA-Leu-Trp plates, which were incubated for 3 d or 4 d at 30°C (**A**, **B**, and **C**). In the case of the *MAK5*/*RPL14A* double shuffle strain, transformed cells were restreaked, after plasmid shuffling on plates containing 5-FoA, on SC-Leu-Trp plates and then spotted in 10-fold serial dilution steps onto SC-Leu-Trp plates, which were incubated for 3 d at 30°C (**D**).

To confirm that the identified sl-mutant alleles were indeed the source of the sl-relation with the *mak5.G218D* or mak5.R728* alleles, we retested their genetic interactions in the setting of *de novo* created double shuffle strains (for the essential *EBP2* and *RPF1* genes and the quasi-essential *RPL14A* gene) or a *MAK5*/*∆nop16* shuffle strain. As expected, the ebp2.K287* allele showed a synthetic lethal phenotype with the *mak5.G218D* mutant. However, the slower-growing C-terminal deletion mutants of *mak5* (mak5.R728* and *mak5.N723*) only exhibited a synthetic enhancement (se) phenotype when combined with the ebp2.K287* allele (Figure 5A). Conversely, combining the mak5.R728* or *mak5.N723* alleles with the *∆nop16* null mutation resulted in synthetic lethality, while the *mak5*.*G218D*/*∆nop16* pair only exhibited a mild se-phenotype (Figure 5B). In agreement with the severe se-phenotype of the two original *RPF1*-complemented sl-mutants isolated by the mak5.R728* sl-screen (data not shown), the distinct mak5.R728*/*rpf1.L199P* and mak5.R728*/*rpf1.C139R* mutant combinations grew clearly slower than the individual mutant strains alone. The synthetic growth defect was much more pronounced, resulting almost in lethality, when the two *rpf1* alleles were combined with the *mak5.N723* allele; however, no se-phenotype could be discerned when paired with the *mak5.G218D* allele (Figure 5C). Due to the non-lethality of the *∆rpl14a* null mutant, we assessed the growth of mak5/rpl14a.L123* double mutants on synthetic medium after plasmid shuffling on 5-FoA-containing plates. In agreement with a complete synthetic effect of the mak5.R728* allele on the rpl14a.L123* allele, mak5.R728*/rpl14a.L123* and mak5.R728*/*∆rpl14a* cells showed the same severe growth defect, which was identical to the one of the *∆rpl14a* single mutant strain (Figure 5D). Similarly, a synthetic relation was observed between the *mak5.N723* and rpl14a.L123* alleles, while the rpl14a.L123* allele exerted only a minor effect on the growth of *mak5.G218D* mutant cells. On the other hand, there was no se-phenotype associated with any of the *mak5*/*∆rpl14b* combinations (data not shown). We conclude that the expression of C-terminally truncated Rpl14a, and not the lower cellular Rpl14 levels (as in *∆rpl14a* or *∆rpl14b* cells), causes a synergistic pre-60S maturation defect specifically in conjunction with the expression of Mak5 variants lacking part of the C-terminal extension. The allele specificity of the observed genetic interactions is particularly striking for the ebp2.K287* allele, which, unlike the *rpf1* mutants or the *∆nop16* null mutant that are mainly linked to the mak5.R728* and *mak5.N723* alleles, shows synthetic lethality only when combined with the *mak5.G218D* allele (see also Discussion).

**Figure 6:**
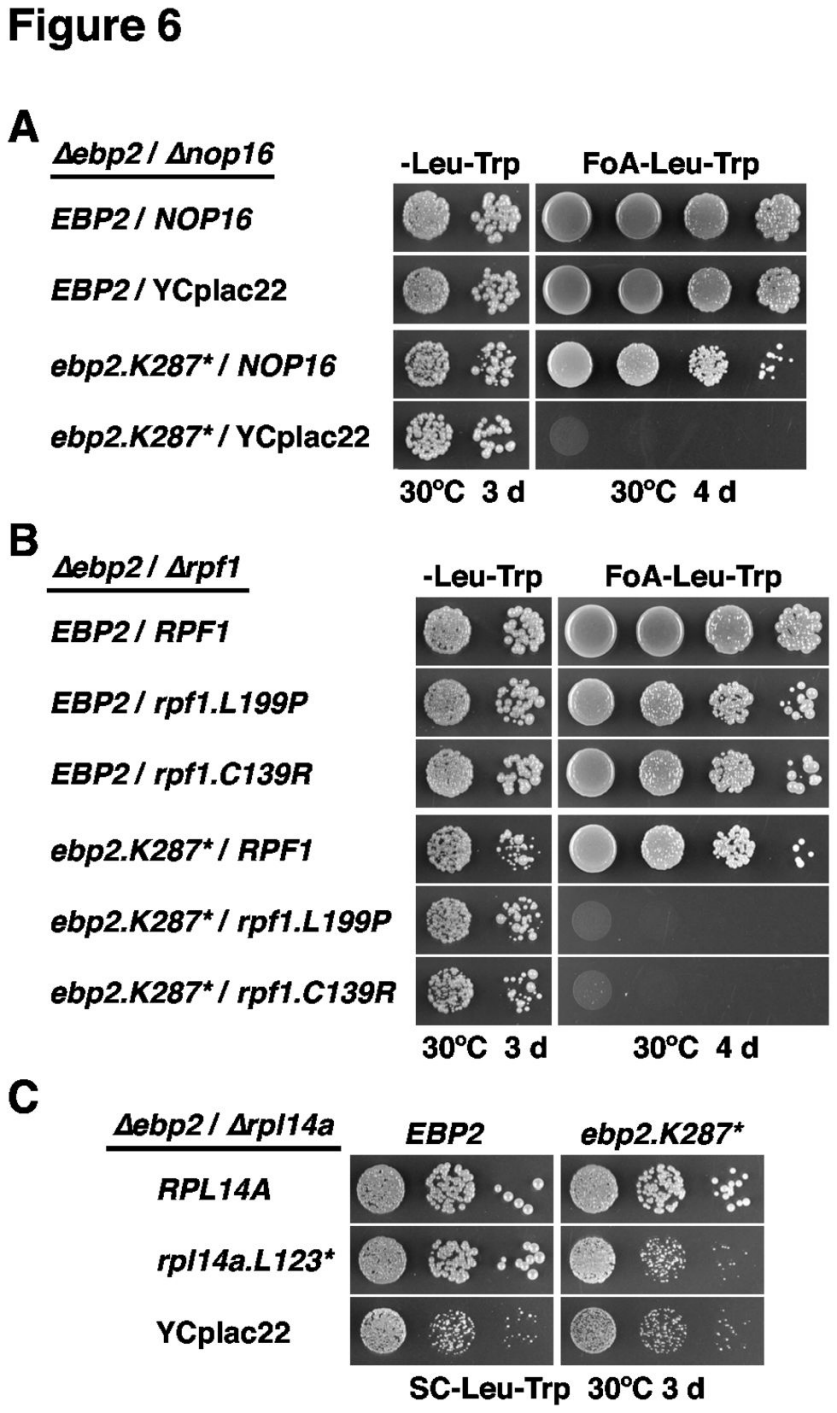
Synthetic lethal interactions between the ebp2.K287* allele and *∆nop16*, *rpf1* and *rpl14a* alleles. *EBP2*/*∆nop16* (**A**), *EBP2*/*RPF1* (**B**) and *EBP2*/*RPL14A* (**C**) shuffle or double shuffle strains were co-transformed with plasmids harbouring the indicated wild-type and mutant alleles and/or empty vector (YCplac22). Cells were restreaked on SC-Leu-Trp plates and then spotted in 10-fold serial dilution steps onto SC-Leu-Trp and SC+5-FoA-Leu-Trp plates, which were incubated for 3 d or 4 d at 30°C (**A** and **B**). In the case of the *EBP2*/*RPL14A* double shuffle strain, transformed cells were restreaked, after plasmid shuffling on plates containing 5-FoA, on SC-Leu-Trp plates and then spotted in 10-fold serial dilution steps onto SC-Leu-Trp plates, which were incubated for 3 d at 30°C (**C**).

With the aim of establishing a complete genetic network, we next tested the alleles identified by the sl-screens among each other for the enhancement of their respective growth phenotypes. Most strikingly, combination of the ebp2.K287* allele with either of the two *rpf1* alleles or the *∆nop16* null allele resulted in synthetic lethality (Figure 6A and 6B). Moreover, the ebp2.K287*/rpl14a.L123* double mutant showed the same severe slow-growth phenotype as the *∆rpl14a* single or the ebp2.K287*/*∆rpl14a* double mutant, revealing that the ebp2.K287* allele minimized the functionality of the rpl14a.L123* mutant to the extent of a complete *∆rpl14a* null mutation (Figure 6C). Combining the *∆nop16* null mutation with the *rpf1* or rpl14a.L123* alleles evoked a clear and very mild se-phenotype, respectively (Figures S13A and S13B). Finally, we could not observe any synergistic growth defect in the case of rpf1/rpl14a.L123* double mutant cells (Figure S13C). We conclude that Mak5, Ebp2, Nop16, Rpl14a and Rpf1 form a functional, genetically defined, cluster of proteins that likely act at a similar step during pre-60S maturation. Moreover, allele-specificity and strength of the interactions suggest a most intimate interplay between Mak5, Ebp2 and Rpl14.

### The central conserved domain of Ebp2 is sufficient for its functionality

**Figure 7:**
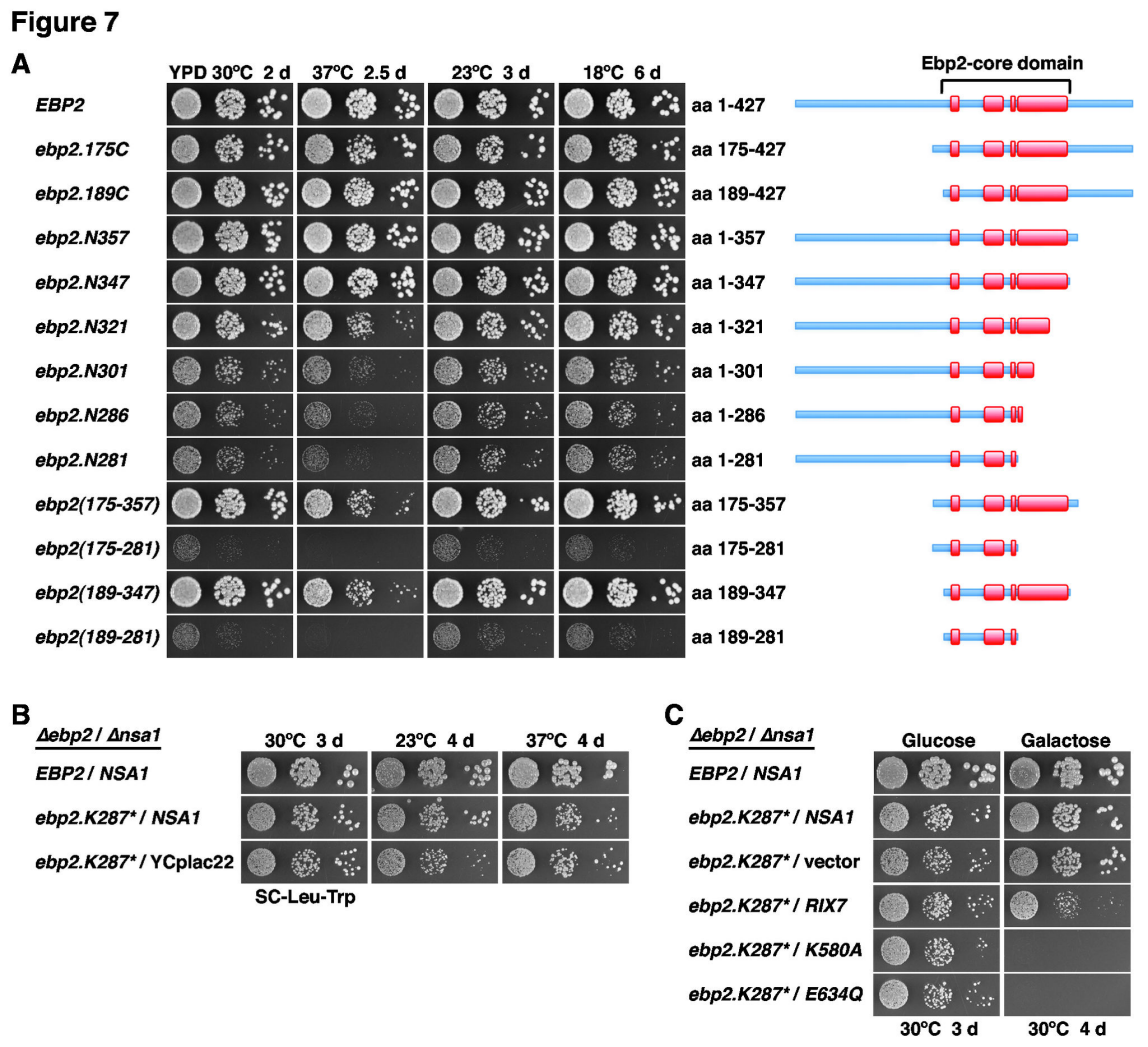
Ebp2 contains an essential, central core domain and the ebp2.K287* allele suppresses *∆nsa1* lethality. (**A**) Growth phenotypes of viable *ebp2* deletion mutants. Plasmid-borne wild-type EBP2 or the indicated *ebp2* deletion mutants under the control of the authentic promoter were transformed into the EBP2 shuffle strain. After plasmid shuffling on plates containing 5-FoA, cells were restreaked on YPD plates and then spotted in 10-fold serial dilution steps onto YPD plates, which were incubated for 2 d at 30°C, 2.5 d at 37°C, 3 d at 23°C and 6 d at 18°C. The proteins encoded by the N- and/or C-terminally truncated *ebp2* mutants are schematically depicted on the right, predicted α-helices within the central Ebp2-core domain are highlighted in red. (**B**) Bypass suppression of *∆nsa1* null lethality by the ebp2.K287* allele. The *EBP2*/*NSA1* double shuffle strain was co-transformed with plasmids harbouring the EBP2 wild-type gene or the ebp2.K287* allele and a plasmid carrying NSA1 or the empty vector (YCplac22). After plasmid shuffling on plates containing 5-FoA, cells were restreaked on SC-Leu-Trp plates and then spotted in 10-fold serial dilution steps onto SC-Leu-Trp plates, which were incubated for 3 d at 30°C, 4 d at 23°C and 4 d at 37°C. (**C**) Dominant-negative RIX7 alleles confer lethality to ebp2.K287*/*∆nsa1* cells. The *EBP2*/*NSA1* double shuffle strain was co-transformed with plasmids harbouring the EBP2 wild-type gene or the ebp2.K287* allele and plasmids carrying the NSA1 wild-type gene or, expressed under the control of the *GAL1-10* promoter, wild-type RIX7 and the dominant-negative *RIX7.K580A* or *RIX7.E634Q* alleles or empty vector. After plasmid shuffling on plates containing 5-FoA, cells were restreaked on SC-Leu-Trp plates and then spotted in 10-fold serial dilution steps onto SC-Leu-Trp (Glucose) and SGal-Leu-Trp (Galactose) plates, which were incubated for 3 d or 4 d at 30°C, respectively.

The finding that the ebp2.K287* allele, which introduces a deletion of the C-terminal 141 amino acids of Ebp2, clearly supported growth is different from previously published results showing that deletion of the C-terminal 105 amino acids conferred lethality (Figure S7B and [83]). We therefore carefully re-examined the boundaries that still enable the expression of fully or partially functional Ebp2 variants by a detailed deletion analysis. Ebp2 is composed of a highly conserved, central domain that is predicted to be built up by four conserved α-helices (Figures S7A and S14A). This central domain is flanked by an N-terminal extension of ~185 amino acids, containing a basic region followed by an acidic region, that is substantially shorter or almost completely absent from the orthologous *S. pombe* or *H. sapiens* Ebp2 proteins, respectively (Figure S7A). On the other hand, the C-terminal extension, defined here as starting after the fourth predicted central α-helix of ~60 amino acid length, comprises the terminal ~80 amino acids of Ebp2 and contains some well-conserved stretches. Deletion of the N-terminal extension (*ebp2.175C* and *ebp2.189C*; amino acids 175-427 and 189-427, respectively), as previously observed for the N∆178 *ebp2* construct [83], resulted in a fully functional Ebp2 variant (Figures 7A and S14B). Further N-terminal deletion constructs (*ebp2.211C* and *ebp2.229C*), removing the first predicted α-helix of the central domain, did not support growth (Figure S14B). Notably, Ebp2 variants lacking most of or the complete C-terminal extension (*ebp2.N357* and *ebp2.N347*; amino acids 1-357 and 1-347, respectively), showed wild-type growth at all tested temperatures (Figure 7A). To further delineate the C-terminal border, we tested the growth of additional deletion constructs (*ebp2.N321*, *ebp2.N301*, *ebp2.N286* and *ebp2.N281*), remarkably revealing that even the construct lacking the complete last predicted α-helix of the central domain supported growth (Figure 7A). The slow-growth phenotype associated with this *ebp2.N281* allele was almost identical to the one of the *ebp2.N286* allele, which corresponds to a ‘clean’ variant of the original ebp2.K287* mutant. Next, we determined whether the central Ebp2 domain would be sufficient to mediate the function of Ebp2. The constructs lacking both the N- and C-terminal extensions, *ebp2*(*175-357*) and *ebp2*(*189-347*), conferred wild-type growth at all temperatures, except at 37°C where a growth defect was clearly visible (Figure 7A). Furthermore, the *ebp2*(*175-281*) and *ebp2*(*189-281*) constructs, having additionally the long predicted α-helix deleted from the C-terminus of the central domain, still sustained growth, albeit at much lower rate (Figures 7A and S14B). Altogether, we conclude that Ebp2 contains a conserved central domain, referred to as the Ebp2-core domain (amino acids 189-347), which forms the minimal unit ensuring almost complete functionality. Strikingly, deletion of the prominent C-terminal α-helix from the Ebp2-core domain still supports growth, indicating that the minimal structural and functional information for Ebp2 to at least partially fulfil its essential task resides in a short segment of ~90 amino acids between residues 189 and 281.

### The ebp2.K287* allele suppresses the lethality of the *∆nsa1* null mutant

Next, we tested the capability of the *∆nop16* allele as well as the *ebp2*, *rpl14a* and *rpf1* alleles, isolated *via* the *mak5* sl-screens, to suppress the lethality of the *∆nsa1* null mutant. To this end, we generated double shuffle strains (for the essential *EBP2* and *RPF1* genes and the quasi-essential *RPL14A* gene) or an *NSA1*/*∆nop16* shuffle strain. While expression of the ebp2.K287* mutant was promoting robust growth in the absence of Nsa1 (Figure S15A), the *∆nop16*, rpl14a.L123*, *rpf1.L199P* and *rpf1.C139R* alleles did not restore growth of *∆nsa1* null mutant cells (Figure S15B-D). To better assess the extent of suppression, we compared the growth properties of ebp2.K287* cells in the presence and absence of *NSA1* after plasmid shuffling on 5-FoA-containing plates. This analysis revealed that the ebp2.K287* allele very efficiently suppressed the lethality of the *∆nsa1* null mutant (Figure 7B). As observed before for the mak5.R728*, *nop1.M232K* and *nop4.S460L* alleles (Figure 1A-C), suppression was more efficient at 30°C than at 23°C (Figure 7B); moreover, growth of the ebp2.K287* mutant was even slightly better in the absence than in the presence of Nsa1 at 37°C (Figure 7B). Moreover, in validation of the above findings that dominant-negative alleles of *RIX7* still exert their phenotypes in the mak5.R728* and *nop4.S460L ∆nsa1* null ’suppressor’ strains (Figure 3A and 3B), galactose-induced over-expression of the dominant-negative *RIX7.K580A* and *E634Q* mutants also conferred lethality to ebp2.K287*-suppressed *∆nsa1* null mutant cells (Figure 7C). As observed for the mak5.R728* ∆nsa1 null ’suppressor’ strain (Figure 3A), over-expression of wild-type *RIX7* already had a strong negative effect on the growth of ebp2.K287* cells lacking Nsa1 (Figure 7C).

### Rix7 over-expression reduces growth and 60S formation in *mak5* and *ebp2* mutants

**Figure 8:**
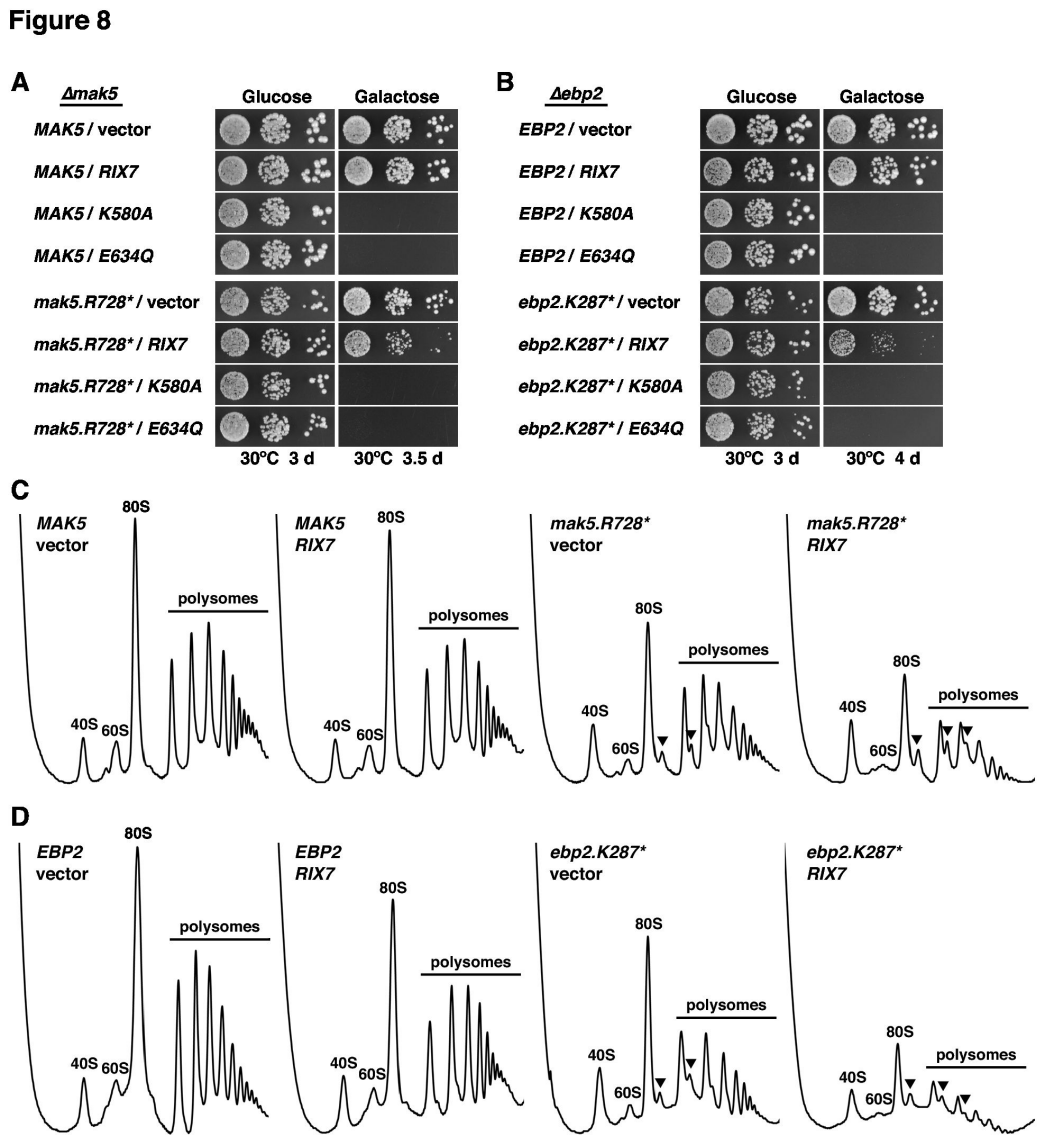
Over-expression of *RIX7* enhances the growth defect and 60S subunit deficiency of mak5.R728* and ebp2.K287* mutants. The MAK5 (**A**) and EBP2 (**B**) shuffle strains were co-transformed with plasmids harbouring either the MAK5 or EBP2 wild-type genes or the mak5.R728* or ebp2.K287* mutant alleles and empty vector or plasmids expressing wild-type RIX7 and the dominant-negative *RIX7.K580A* or *RIX7.E634Q* alleles under the control of the *GAL1-10* promoter. After plasmid shuffling on plates containing 5-FoA, cells were restreaked on SC-Leu-Trp plates and then spotted in 10-fold serial dilution steps onto SC-Leu-Trp (Glucose) and SGal-Leu-Trp (Galactose) plates, which were incubated at 30°C for 3 d, 3.5 d or 4 d, respectively. (**C** and **D**) Over-expression of RIX7 affects 60S subunit biogenesis in mak5.R728* and ebp2.K287* mutant cells. The MAK5 (**C**) and EBP2 (**D**) shuffle strains were co-transformed with plasmids harbouring either the MAK5 or EBP2 wild-type genes or the mak5.R728* or ebp2.K287* mutant alleles and empty vector or a plasmid expressing wild-type RIX7 under the control of the *GAL1-10* promoter. After plasmid shuffling on plates containing 5-FoA, cells were restreaked on SC-Leu-Trp plates. Transformed cells were first pre-grown in SC-Leu-Trp medium, then diluted into SC-Leu-Trp medium with raffinose as carbon source and, finally, expression of wild-type RIX7 was induced by addition of galactose to the medium. After ~16 h of galactose induction, cell extracts were prepared under polysome-conserving conditions and eight A_260_ units were resolved in 10-50% sucrose gradients. The absorption profiles were recorded by continuous monitoring at A_254_. Sedimentation is from left to right. The peaks of free 40S and 60S subunits, 80S free couples/monosomes and polysomes are indicated. Half-mers are highlighted by arrowheads.

To expand on the observation that over-expression of wild-type *RIX7* particularly affected the growth of the mak5.R728* and ebp2.K287* alleles in cells lacking Nsa1 (Figures 3A and 7C), we next determined the effects of galactose-induced over-expression of *RIX7* from the *GAL1-10* promoter on mak5.R728* and ebp2.K287* mutant cells in the presence of Nsa1. While over-expression of *RIX7* had only a very minor effect on the growth of *MAK5* and *EBP2* wild-type cells, growth of the mak5.R728* and, even more dramatically, of the ebp2.K287* mutant was severely affected by *RIX7* over-expression (Figure 8A and 8B). For control purposes, we also tested over-expression of the dominant-negative *RIX7.K580A* and *E634Q* mutants, conferring, as expected, lethality to wild-type as well as mak5.R728* and ebp2.K287* cells.

Next, we assessed the effects of *RIX7* over-expression on 60S subunit biogenesis by polysome profile analysis. To this end, we pre-grew cells in synthetic liquid medium with raffinose as carbon source and then induced *RIX7* expression by addition of galactose for ~16 h before preparing the cell extracts. In agreement with the mild effect on growth, *RIX7* over-expression only resulted in very subtle changes of the polysome profile of *MAK5* and *EBP2* wild-type cells, as indicated by a slight decrease in free 60S subunits (Figure 8C and 8D). On the other hand, over-expression of *RIX7* in mak5.R728* and ebp2.K287* cells aggravated their 60S subunit deficiency, as evidenced by a further decrease in free 60S subunits and a drastic reduction of polysome content (Figure 8C and 8D). We conclude that Rix7, when highly abundant, negatively acts on pre-60S subunits from *mak5* and *ebp2* mutant cells, which are likely structurally deranged and might therefore be prematurely channelled into a clearance pathway by excess Rix7 (see Discussion).

## Discussion

### A genetic network defines the ’Mak5 cluster’ as a novel 60S biogenesis module

**Figure 9:**
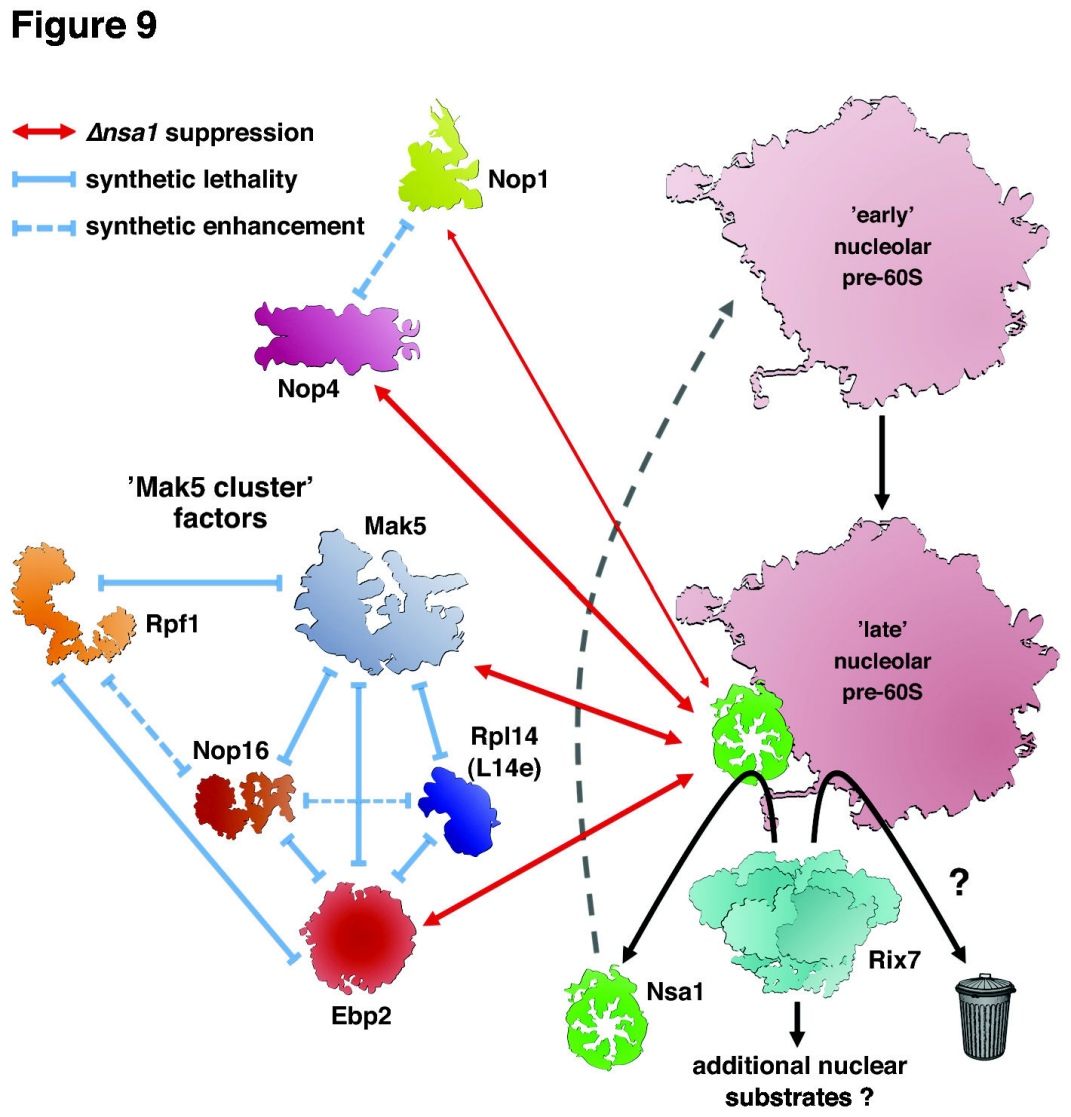
Model summarizing the genetic networks established in this study. Nsa1 (green) is a component of late nucleolar pre-60S particles (light bordeaux) whose release and recycling is mediated by the AAA-type ATPase Rix7 (turqoise) [39]. Mutant alleles of the genes encoding the 60S biogenesis factor Ebp2 (red), the DEAD-box RNA helicase Mak5 (light blue), the methyltransferase component of C/D-box snoRNPs Nop1 (chartreuse) and the RRM-containing RNA-binding protein Nop4 (pink) suppress the lethality of *∆nsa1* null mutant cells. These bypass suppressions are indicated by red double arrows, whose line thickness correlates with the observed strength of the suppression. Mak5 forms a genetic network with Ebp2, Nop16, Rpf1 and the r-protein Rpl14. Synthetic lethal and synthetic enhancement interactions amongst these are depicted by blue continuous or dashed ’negative’ arrows, respectively. We propose that Ebp2, Mak5, Nop16 and Rpf1, referred to as the ’Mak5 cluster’ factors, may orchestrate the structural arrangement of a eukaryote-specific 60S subunit surface composed of the r-proteins Rpl6, Rpl14 and Rpl16 and the rRNA expansion segments ES7L and ES39L (not depicted). Since dominant-negative alleles of RIX7 retain their phenotype in the absence of Nsa1, Rix7 may have additional nuclear substrates besides Nsa1. Finally, over-expression of Rix7 negatively affects growth of *mak5* and *ebp2* mutant cells both in the absence and presence of Nsa1, suggesting that Rix7, at least when excessively abundant, may act on structurally defective pre-60S subunits and may subject them to degradation (gray trashcan). We therefore propose that Rix7 may, besides specifically releasing and recycling Nsa1, sense the structural integrity of pre-60S particles and, if these are excessively damaged, channel them into a clearance pathway.

In this study, we have identified, based on synthetic lethal interactions, the functional environment around the DEAD-box protein Mak5, which includes Ebp2, Nop16, Rpf1 and the r-protein Rpl14 (Figure 9). The intimate genetic interconnection between all four protein trans-acting factors indicates that Mak5, Ebp2, Nop16 and Rpf1 constitute a novel biogenesis cluster, which we propose to refer to as the ’Mak5 cluster’. Importantly, our genetic analysis represents the crucial first step towards the elucidation of the precise molecular function of Mak5, in conjunction with its genetically defined partner proteins, in one of the multiple assembly steps during the nucleolar phase of pre-60S maturation. Several lines of evidence support the genetic conclusion that Mak5, Ebp2, Nop16 and Rpf1 form a functional unit acting together at a distinct step during the assembly of 60S subunits. First, Ebp2, Nop16 and Rpf1 are stoichiometric components of the late nucleolar pre-60S particle defined by the Nsa1-TAP bait [39], and we have shown here that Mak5 is also associated with the Nsa1 pre-60S particle (Figure 4). Notably, these four biogenesis factors are absent from the nucleoplasmic, Rix1-defined pre-60S particle (Figure 4 and [40]). In agreement, affinity purifications of Ebp2-TAP and Rpf1-HA reveal, as observed for the Nsa1 pre-60S particle, a predominant association with 27SB pre-rRNAs [39,73,85]. Moreover, genetic depletion or mutational perturbation of Mak5, Ebp2 and Rpf1 elicit similar pre-rRNA processing phenotypes, i.e. delayed conversion of 27SA_2_ into 27SB pre-rRNAs and reduced formation of mature 25S rRNA from the 27SB pre-rRNAs [58,83–85,89], altogether indicating an instability of 27SB pre-rRNA containing pre-60S ribosomes. Moreover, mutations in *ebp2* and *mak5* likely entail similar structural alterations within pre-60S particles since both the ebp2.K287* and the mak5.R728* allele suppress the lethality of *∆nsa1* null mutant cells (Figures 1A and 7B). Finally, in support of the specificity of the genetic interactions reported here, there have previously no synthetic lethal relations been unveiled for Nop16, Rpf1 and Rpl14. A sl-screen with the *ebp2-14* allele, however, recently revealed a functional interaction between Ebp2 and Brx1 [73], which is, as Rpf1, a member of the σ^70^-like motif family of RNA-binding ribosome biogenesis factors [85,90]. Yeast two-hybrid data further suggest a direct physical interaction between Ebp2 and Brx1 [73]. However, Brx1 clearly remains associated with pre-60S particles that already contain 7S pre-rRNA and mature 25S and 5.8S rRNAs [73,85], and its genetic depletion mainly leads to the accumulation of the 27SA_2_ pre-rRNA [90]. Altogether these observations suggest an additional or slightly different role for Brx1, therefore, future experiments are needed to address the interesting possibility that Brx1 might also be functionally connected with the other ’Mak5 cluster’ factors.

### The ’Mak5 cluster’ factors may orchestrate the structural arrangement of a eukaryote-specific 60S subunit surface composed of r-proteins Rpl6, Rpl14, and Rpl16 and rRNA expansion segments ES7L and ES39L

What could be the role(s) of the ’Mak5 cluster’ during pre-60S maturation? The functional connection between the ’Mak5 cluster’ factors and the r-protein Rpl14a may offer valuable insight into the potential role of these biogenesis factors. The almost exclusively eukaryote-specific Rpl14 (L14e) is located close to the P-stalk on the solvent-side of the 60S r-subunit (Figure S10B and [56]). Interestingly, Rpl14 is found in some, but not all, archaeal organisms; its two prominent C-terminal α-helices at the least, however, are strictly eukaryote specific (Figure S11A). The α-helical part of Rpl14, not including its C-terminal α-helix, is clamped between the long bent helix of the eukaryote-specific rRNA expansion segment ES7L and a short single-stranded region of ES39L on the surface of 60S subunits (Figure S10C and [56]). On the other hand, the β-stranded part of Rpl14 dives a little bit deeper into the 60S subunit where it makes both contact with the eukaryote-specific r-protein Rpl20 (L20e) and the backbone of a short segment (nucleotides 1183-1185) of the 25S rRNA [56]. The N-terminal extension of Rpl14 contacts, by forming a short parallel β-strand, a three-stranded β-sheet of Rpl9 (L6) (Figure S10C and [56]), however, this interaction does not seem to take place in higher eukaryotic 60S subunits (Figure S10A and [57]). Most notably, the eukaryote-specific C-terminal α-helix of Rpl14, which is truncated in the rpl14a.L123* allele (Figures S10A and S10C), is involved in a series of eukaryote-specific interactions, comprising contacts with (i) the long C-terminal, eukaryote-specific α-helix of Rpl16 (L13), (ii) Rpl6 (L6e) and (iii) ES39L (Figures S10C and S11B; [56]). The C-terminal α-helices of Rpl14 and Rpl16 are sandwiched between two distinct structural elements of ES39L, and Rpl6, which is located above the early-assembling Rpl33 (L33e) [91], connects the base of the long bent helix of ES7L with ES39L (see Figure S10C). While the time point of Rpl14 assembly has not been experimentally addressed, Rpl6 and Rpl16 are likely among the very early assembling large subunit r-proteins [91]. Intriguingly however, Rpl14 was not detected in the mass-spectrometric analysis of low-molecular weight proteins of the Ssf1-TAP purification [74], but it was present in Nug1- or Rix1-purified pre-60S particles [40,92], thus suggesting that Rpl14 may only be assembled at the level of late nucleolar pre-60S intermediates. Taken together, the observed genetic connection between Rpl14 and the factors of the ’Mak5 cluster’ may therefore hint at a function of these eukaryote-specific biogenesis factors in the orchestration of the structural arrangement of the eukaryote-specific 60S subunit surface made up of the r-proteins Rpl6, Rpl14 and Rpl16 and the expansion segments ES7L and ES39L. In analogy to the potential role of the Dbp6-containing sub-complex in facilitating incorporation of the r-protein Rpl3 [93,94], the ’Mak5 cluster’ factors might be required for the assembly and stable association of the r-protein Rpl14. At this stage, however, we are still far from understanding the exact molecular role of the ’Mak5 cluster’ factors. Clearly, future experiments are required to corroborate an involvement of the ’Mak5 cluster’ factors in the structural arrangement of this eukaryote-specific 60S subunit surface, for example by defining the (pre-)rRNA binding sites of the ’Mak5 cluster’ factors by CRAC and by structural probing of the local alterations entailed by dysfunctionality of these factors.

Additional open questions concern the recruitment of the putative RNA-dependent ATPase Mak5 to and its activation on pre-60S particles. Since Ebp2, Nop16 and Rpf1, unlike the weakly or transiently associated Mak5, are stoichiometric components of the Nsa1-defined pre-60S particle (Figure 4 and [39]), it can be concluded that these proteins bind independently of Mak5 to pre-60S particles. However, future studies are required to assess whether recruitment of Mak5 to pre-60S particles is dependent on the integrity of its functionally interacting partners. Since our deletion analysis showed that the C-terminal extension, contrary to the N-terminal extension, of Mak5 harbours an essential function (Figure S5B), it is very likely that this C-terminal domain is involved in promoting recognition of the cognate substrate, either by directly interacting with the (pre-)rRNA or by binding to a dedicated, pre-60S associated co-factor. We have so far no evidence for direct physical interactions, as indicated by yeast two-hybrid analyses (data not shown), amongst any of the ’Mak5 cluster’ factors and of these with Rpl14. This suggests that Ebp2, Nop16, Rpf1 and Rpl14 may arrange the local RNP structure such that Mak5 can be efficiently recruited to and activated by its distinct (pre-)rRNA or RNP binding site or, alternatively, that our sl-screen with *mak5* alleles has not yet identified the co-factor responsible for recruitment and/or activation of Mak5. Besides assessing the recruitment of Mak5, it will also be informative to address the interdependence of pre-60S association amongst Ebp2, Nop16, Rpf1 and Rpl14. Our genetic data point for example to the possibility that Rpl14a with a truncated C-terminal α-helix may be no longer recruited to pre-60S subunits in ebp2.K287* or mak5.R728* and *mak5.N723* mutant cells (Figures 5D and 6C). Furthermore, the distinct allele specificity of the *ebp2*/*mak5* sl-relation, i.e. ebp2.K287* is the only allele identified by the *mak5* sl-screens conferring synthetic lethality in combination with the *mak5.G218D* allele (Figure 5), indicates that Ebp2 is particularly linked to the enzymatic activity of Mak5. However, future studies will be required to address how the local, Ebp2-imposed (pre-)rRNA or RNP structure within late nucleolar pre-60S particles might contribute to the activation of Mak5. 

### Mutations in *EBP2*, *MAK5*, *NOP1* and *NOP4* bypass the requirement for the essential 60S biogenesis factor Nsa1

Another interesting, but still not well understood aspect of our present study is the observation that the requirement for the essential biogenesis factor Nsa1 can be bypassed by mutant alleles of the genes encoding Ebp2, Mak5, Nop1 and Nop4 (Figures 1 and 7B). Since we did not carry out an exhaustive screen for *∆nsa1* suppressor alleles, it is very likely that mutant variants of additional genes may confer suppression. Strikingly, the *nop1.M232K* and *nop4.S460L* suppressor alleles confer only very minor growth and 60S biogenesis defects at their optimal *∆nsa1* suppression temperature of 30°C (Figures S2B and S2C). While the suppression by the *nop1.M232K* allele, however, is rather weak, *nop4.S460L* mutant cells grow only slightly slower in the absence of Nsa1 (Figure 1B and 1C). On the other hand, the ebp2.K287* and mak5.R728* alleles confer a clear growth defect (Figures S2A and S7B), nevertheless, they can, especially at 37°C, suppress the absence of Nsa1 to the extent of the growth phenotype of the suppressor mutation (Figures 1A and 7B). Notably, we observed that suppression is in all four cases much less pronounced at 23°C than at 30°C, indicating that the non-recruitment of Nsa1 to pre-60S particles particularly constrains alternative RNP assembly or folding routes at lower temperatures where macromolecular assembly processes are generally less thermodynamically favoured. Since Nop1, besides being associated with the SSU processome / 90S particles [8,9], and Nop4 are present in very early pre-60S particles but are absent from the Nsa1-defined pre-60S particle [39,44,70–72,74], they act on pre-60S particles upstream of the Nsa1 recruitment step. The synthetic enhancement between the *nop1.M232K* and *nop4.S460L ∆nsa1* suppressor alleles suggests that these may affect a similar step of pre-60S assembly (Figure S3). We assume that partial dysfunctionality of Nop1 and Nop4 compensates the lack of Nsa1 on late nucleolar pre-60S particles by having locally altered the RNP structure and/or pre-rRNA folding within very early pre-60S particles, thus permitting alternative assembly and folding intermediates. Alternatively, reduced Nop1 and Nop4 function may kinetically delay early maturation steps, therefore enabling formation of the correct or an acceptable structural intermediate and allowing efficient progression of downstream assembly events in the absence of Nsa1. However, it is at present, without knowing the rRNA binding site(s) of Nsa1, impossible to draw any conclusion about the possible conformational changes of local RNP or (pre-)rRNA structures within pre-60S particles that permit *nop1.M232K* and *nop4.S460L* mutant cells to efficiently assemble 60S subunits in the absence of Nsa1. Likewise, we will also first need to determine the (pre-)rRNA binding sites of Ebp2 and Mak5 in order to evaluate how reduced activity or pre-60S binding of these factors might influence the secondary or tertiary rRNA structure around the Nsa1 binding surface. Once the rRNA binding sites of Ebp2, Mak5 and in particular Nsa1 are known, chemical probing should reveal how mutation of *ebp2* and *mak5* change the secondary structure of these sites and their adjacent rRNA neighbourhood in the absence of Nsa1. While the association of Ebp2 and Mak5 with Nsa1-containing pre-60S ribosomes suggests that they act at the level of late nucleolar pre-60S particles, we cannot rule out that, due to their recruitment at the level of very early pre-60S particles, such as the Npa1 pre-60S particle [70], the suppression conferred by the *ebp2* and *mak5* alleles is a consequence of the reduced binding of the mutant Ebp2 and Mak5 variants to these very early pre-60S particles.

In conclusion, we have unravelled by genetic means possible structural changes within pre-60S particles that influence their assembly kinetics and/or path in order to compensate for the lack of Nsa1 recruitment. We propose that the systematic analysis of *∆nsa1* suppression by mutants affecting different, from the early to the late nucleolar phase, 60S biogenesis steps would be an ideal assay to further categorize these factors and to obtain insight into their *in vivo* contribution to (pre-)rRNA folding and RNP assembly with respect to the Nsa1 binding surface. In general, we believe that such bypass suppression analyses represent a powerful genetic tool that can be viewed as an *in vivo* structure probing approach to unveil functional connections and structural rearrangements during assembly of pre-ribosomal particles, which can then be examined in detail by *in vivo* or *in vitro* probing of the rRNA structure. Moreover, our genetic findings support the view that assembly of ribosomal subunits does not exclusively occur *via* an ordered series of hierarchical steps, but may also rely on multiple parallel pathways [95,96].

### The AAA-ATPase Rix7 may have additional nuclear functions besides stripping Nsa1 from pre-60S particles

We have previously shown that the AAA-type ATPase Rix7 mediates the release of the essential 60S biogenesis factor Nsa1 from a distinct, late nucleolar pre-60S particle [39]. In this study, we show that dominant-negative *RIX7* alleles still confer lethality to cells capable of growing in the absence of Nsa1 due to the suppression of the lethal *∆nsa1* null mutant phenotype by *ebp2*, *mak5* and *nop4* alleles (Figures 3 and 7C). This observation strongly suggests that Rix7, which localizes throughout the nucleus during exponential growth [97], may have additional nuclear substrates besides Nsa1 (Figure 9). Interestingly, over-expression of wild-type Rix7, both in the presence and absence of Nsa1, severely affects growth of the ebp2.K287* and mak5.R728* mutants (Figures 3A, 7C, 8A and 8B), suggesting that Rix7, at least when abundantly present, may recognize and negatively act on pre-60S particles of *mak5* and *ebp2* mutant cells. Accordingly, we observed that Rix7 over-expression drastically enhances the 60S subunit deficiency of ebp2.K287* and mak5.R728* mutant cells (Figure 8C and 8D). Since there is no negative effect associated with Rix7 over-expression in wild-type cells, we conclude that Rix7 may somehow specifically recognize partially defective pre-60S particles that can, at normal Rix7 expression levels, to a certain extent still productively assemble into mature 60S subunits. We therefore propose that Rix7 may, besides specifically releasing and recycling Nsa1, sense the structural integrity of pre-60S particles and, if these are excessively damaged, channel them into a clearance pathway (Figure 9). At present it is not clear whether the proposed clearance pathway depends on Nsa1 at normal Rix7 expression levels and how Rix7 may recognize structurally hampered pre-60S subunits. In any case, the almost exclusive association of Rix7 with the Nsa1-defined pre-60S particle indicates that Rix7 most likely performs the integrity-sensing function at the level of late nucleolar pre-60S particles before their arrival in the nucleoplasm, where a series of further maturation steps promote acquisition of export competence [2,39]. In light of the substantial homology between Rix7 and Cdc48/p97 [4], a dual role for Rix7 in biogenesis factor recycling (Nsa1) and in targeting defective pre-60S ribosomes for degradation would not be surprising. Many of the diverse cellular functions of Cdc48/p97 are linked to the recognition, either direct or indirect *via* substrate-recruiting co-factors, of ubiquitinated substrate proteins and their degradation by the proteasome [41,42]. Moreover, the fate of these substrate proteins depends on their ubiquitination status and the recruited co-factors, as they can either be channelled to degradation or released as stable mono-ubiquitinated or unmodified proteins [41,42]. Future efforts will be required to reveal whether defective pre-60S particles undergo ubiquitination and whether Rix7 indeed recognizes these by binding to a distinct or to several ubiquitinated pre-60S factor(s). Furthermore, the connection between the potential Rix7 clearance pathway and the exosome-mediated degradation of polyadenylated (pre-)rRNAs needs to be explored [23–25]. Altogether, we propose that Rix7 may have additional, yet to be identified nuclear functions, likely involving the structural rearrangement of macromolecular assemblies, besides releasing Nsa1 from pre-60S particles. Moreover, Rix7 may, possibly independently of Nsa1, act on faultily assembled pre-60S particles in the framework of a regular clearance pathway. Clearly, future experiments are required to illuminate these exciting possibilities and understand how Rix7 exerts its potentially diverse nuclear functions.

## Supporting Information

Table S1 **Yeast strains used in this study.**
(PDF)

Table S2 **Plasmids used in this study.**
(PDF)

Figure S1 **Isolation of spontaneous bypass suppressors of the lethal *∆nsa1* null mutant phenotype.** The *NSA1* shuffle strain was transformed with plasmids carrying *NSA1* under the control of the authentic promoter or human NSA1, encoding only the predicted WD-40 β-propeller (hNSA1.N328), under the control of the strong *ADH1* promoter. After plasmid shuffling on plates containing 5-FoA, cells were restreaked on YPD plates and then spotted in 10-fold serial dilution steps onto YPD plates, which were incubated for 7 d at 23°C, 5 d at 30°C and 5 d at 37°C. While hNSA1.N328 complements very weakly the lethality of *∆nsa1* null mutant cells, spontaneous suppressors of this slow-growth phenotype arise with high frequency. Subsequent cloning and allele sequencing revealed that suppressor strains S4 and S5 contain the *nop4.S460L* and mak5.R728* alleles, respectively.
(PDF)

Figure S2 **Growth and polysome profile analyses of *∆nsa1* suppressor alleles.** *MAK5* (**A**), *NOP1* (**B**) and *NOP4* (**C**) shuffle strains were transformed with plasmids harbouring, under the control of the authentic promoters, either the *MAK5*, *NOP1* or *NOP4* wild-type genes or the mak5.R728*, *nop1.M232K* or *nop4.S460L* mutant alleles, respectively. After plasmid shuffling on plates containing 5-FoA, cells were restreaked on YPD plates and then spotted in 10-fold serial dilution steps onto YPD plates, which were incubated for the indicated times at 30°C, 37°C, 23°C and 18°C (upper parts). Polysome profiles of the above wild-type and mutant strains are shown in the lower parts of each subfigure. Briefly, cells were grown in YPD medium to an OD_600_ of ~0.8 at 30°C or shifted for 3 h to 37°C. Cell extracts were prepared under polysome-conserving conditions and eight A_260_ units were resolved in 10-50% sucrose gradients. The absorption profiles were recorded by continuous monitoring at A_254_. Sedimentation is from left to right. The peaks of free 40S and 60S subunits, 80S free couples/monosomes and polysomes are indicated. Half-mers are highlighted by arrowheads. The polysome profiles of *NOP1* and *NOP4* wild-type strains shifted for 3 h to 37°C are very similar to the ones obtained at 30°C and have been therefore omitted to increase the clarity of the Figure.
(PDF)

Figure S3 **The *nop1.M232K* and *nop4.S460L* alleles synergistically affect growth.** The *NOP1*/*NOP4* double shuffle strain was co-transformed with plasmids harbouring wild-type *NOP1* or the *nop1.M232K* allele and wild-type *NOP4* or the *nop4.S460L* allele. Cells were restreaked, after plasmid shuffling on plates containing 5-FoA, on YPD plates and then spotted in 10-fold serial dilution steps onto YPD plates, which were incubated for 2 d at 30°C, 3 d at 23°C and 3 d at 37°C.
(PDF)

Figure S4 The mak5.R728*, *nop1.M232K* and *nop4.S460L* alleles suppress the lethality of *∆nsa1* null mutant cells. *MAK5*/*NSA1* (**A**), *NOP1*/*NSA1* (**B**) and *NOP4*/*NSA1* (**C**) double shuffle strains were co-transformed with plasmids harbouring the indicated wild-type and mutant alleles and/or empty vectors (YCplac111 or YCplac22). Transformed cells were restreaked on SC-Leu-Trp plates and then spotted in 10-fold serial dilution steps onto SC-Leu-Trp and SC+5-FoA-Leu-Trp plates, which were incubated for 3 d at 30°C and 4 d or 6 d at 30°C, respectively.
(PDF)

Figure S5 **The C-terminal extension to the DEAD-box core of Mak5 harbours an essential function.** (**A**) Multiple sequence alignment, generated in the ClustalW output format with T-Coffee, of the C-terminal extensions of Mak5 from *S. cerevisiae*, *Schizosaccharomyces pombe* (Accession: NP_596107) and *Homo sapiens* (DDX24; Accession: NP_065147). Conserved (*), strongly similar (:) and weakly similar (.) amino acids are indicated below the alignment. The last motif of the DEAD-box core (motif VI, highlighted in green) was used as the conserved starting point for the alignment. The C-terminal end of the DEAD-box core, derived from sequence comparisons and known DEAD-box RNA helicase structures, is indicated by a dashed green line. Secondary structure elements were predicted by PSIPRED; α-helices are highlighted in red and β-strands in blue. Blue arrowheads indicate the C-terminal ends of the different C-terminal deletion constructs. The position of the mak5.R728* mutation is also shown. (**B**) Growth phenotypes of N- and C-terminal *mak5* deletion mutants. Plasmid-borne wild-type *MAK5* or the indicated *mak5* deletion mutants, all under the control of the authentic promoter, were transformed into the *MAK5* shuffle strain. Transformed cells were first restreaked on SC-Leu plates and then spotted in 10-fold serial dilution steps onto SC-Leu and SC+5-FoA plates, which were incubated for 3 d or 4 d at 30ºC. The proteins encoded by the N- and/or C-terminally truncated *mak5* mutants are schematically depicted on the right; the DEAD-box core is indicated in green and predicted α-helices within the C-terminal extension are highlighted in red. (**C**) Growth phenotypes of viable N- and C-terminal deletion mutants and the *mak5.G218D* mutant. Plasmid-borne wild-type *MAK5* or the indicated *mak5* mutants, all under the control of the authentic promoter, were transformed into the *MAK5* shuffle strain. After plasmid shuffling on plates containing 5-FoA, cells were restreaked on YPD plates and then spotted in 10-fold serial dilution steps onto YPD plates, which were incubated for 2 d at 30ºC, 2.5 d at 37ºC, 3 d at 23ºC and 6 d at 18ºC.
(PDF)

Figure S6 **The *mak5.166C*, *mak5.N723* and *mak5.G218D* mutants exhibit a deficiency in 60S subunit biogenesis.** Plasmid-borne wild-type *MAK5* or the indicated *mak5* mutants, all under the control of the authentic promoter, were transformed into the *MAK5* shuffle strain. After plasmid shuffling on plates containing 5-FoA, cells were grown in YPD medium to an OD_600_ of ~0.8 at 30°C. Cell extracts were prepared under polysome-conserving conditions and eight A_260_ units were resolved in 10-50% sucrose gradients. The absorption profiles were recorded by continuous monitoring at A_254_. Sedimentation is from left to right. The peaks of free 40S and 60S subunits, 80S free couples/monosomes and polysomes are indicated. Half-mers are highlighted by arrowheads.
(PDF)

Figure S7 Ebp2 contains a conserved α-helical core domain and ebp2.K287* displays a 60S subunit deficiency. (**A**) Multiple sequence alignment, generated in the ClustalW output format with T-Coffee, of Ebp2 from *S. cerevisiae*, *S.* pombe (Accession: NP_593575) and *H. sapiens* (hEBP2 isoform 2; Accession: NP_006815). Conserved (*), strongly similar (:) and weakly similar (.) amino acids are indicated below the alignment. Secondary structure elements were predicted by PSIPRED; α-helices are highlighted in red. The position of the ebp2.K287* mutation, which truncates Ebp2 after amino acid 286, present in sl-mutant strain SL19 is indicated by a blue arrowhead. (**B**) Growth phenotype (upper part) and polysome profile (lower part) of the ebp2.K287* mutant. Plasmid-borne wild-type *EBP2* or the ebp2.K287* allele under the control of the authentic promoter were transformed into the *EBP2* shuffle strain. After plasmid shuffling on plates containing 5-FoA, cells were restreaked on YPD plates and then spotted in 10-fold serial dilution steps onto YPD plates, which were incubated for 2 d at 30ºC, 2.5 d at 37ºC, 2.5 d at 23ºC and 5 d at 18ºC (upper part). Schematic representations of Ebp2 and the C-terminally truncated Ebp2.K287* protein are depicted on the right of the upper part. For simplicity, only the predicted α-helices, highlighted in red, within the conserved Ebp2-core domain are indicated. *EBP2* and ebp2.K287* cells were grown in YPD medium to an OD_600_ of ~0.8 at 30ºC. Cell extracts were prepared under polysome-conserving conditions and eight A_260_ units were resolved in 10-50% sucrose gradients. The absorption profiles were recorded by continuous monitoring at A_254_ (lower part). Sedimentation is from left to right. The peaks of free 40S and 60S subunits, 80S free couples/monosomes and polysomes are indicated. Half-mers are highlighted by arrowheads.
(PDF)

Figure S8 **The *rpf1.L199P* and *rpf1.C139R* mutants are temperature sensitive and display a 60S biogenesis defect.** (**A**) Multiple sequence alignment, generated in the ClustalW output format with T-Coffee, of Rpf1 from *S. cerevisiae*, *S.* pombe (Accession: NP_593868) and *H. sapiens* (Accession: NP_079341). Conserved (*), strongly similar (:) and weakly similar (.) amino acids are indicated below the alignment. Secondary structure elements were predicted by PSIPRED; α-helices are highlighted in red and β-strands in blue. The positions of the *rpf1.L199P* and *rpf1.C139R* mutations, present in sl-mutant strains SL3 and SL33, are indicated by blue arrowheads. The σ^70^-like RNA binding motif, which is conserved among Brix-family ribosome biogenesis factors, is also indicated. (**B**) Growth phenotype (upper part) and polysome profile (lower part) of the *rpf1.L199P* and *rpf1.C139R* mutants. Plasmid-borne wild-type *RPF1* or the *rpf1.L199P* and *rpf1.C139R* alleles under the control of the authentic promoter were transformed into the *RPF1* shuffle strain. After plasmid shuffling on plates containing 5-FoA, cells were restreaked on YPD plates and then spotted in 10-fold serial dilution steps onto YPD plates, which were incubated for 1.5 d at 30ºC, 2 d at 37ºC, 2.5 d at 23ºC and 5 d at 18ºC (upper part). *RPF1*, *rpf1.L199P* and *rpf1.C139R* cells were grown in YPD medium to an OD_600_ of ~0.8 at 30ºC. Cell extracts were prepared under polysome-conserving conditions and eight A_260_ units were resolved in 10-50% sucrose gradients. The absorption profiles were recorded by continuous monitoring at A_254_ (lower part). Sedimentation is from left to right. The peaks of free 40S and 60S subunits, 80S free couples/monosomes and polysomes are indicated. Half-mers are highlighted by arrowheads.
(PDF)

Figure S9 **The *∆nop16*** **null mutant displays only minor growth and 60S biogenesis defects**. (**A**) Multiple sequence alignment, generated in the ClustalW output format with T-Coffee, of Nop16 from *S. cerevisiae*, *S.* pombe (Accession: NP_596824) and *H. sapiens* (hNOP16 isoform 3; Accession: NP_057475). Conserved (*), strongly similar (:) and weakly similar (.) amino acids are indicated below the alignment. Secondary structure elements were predicted by PSIPRED; α-helices are highlighted in red and β-strands in blue. The effects of the *nop16* mutations, present in sl-mutant strains SL2, SL4, SL63, SL67, SL85 and SL113, are shown and their positions indicated by blue arrowheads. (**B**) Growth phenotype (upper part) and polysome profile (lower part) of the *∆nop16* null mutant. The growth of spore clones from a complete tetrad of a heterozygous *NOP16*/*nop16*::natNT2 diploid was assessed by spotting cells in 10-fold serial dilution steps onto YPD plates, which were incubated for 1.5 d at 30ºC, 2 d at 37ºC, 2 d at 23ºC and 4 d at 18ºC (upper part). *NOP16* and *∆nop16* null mutant cells were grown in YPD medium to an OD_600_ of ~0.8 at 30ºC. Cell extracts were prepared under polysome-conserving conditions and eight A_260_ units were resolved in 10-50% sucrose gradients. The absorption profiles were recorded by continuous monitoring at A_254_ (lower part). Sedimentation is from left to right. The peaks of free 40S and 60S subunits, 80S free couples/monosomes and polysomes are indicated. Half-mers are highlighted by arrowheads.
(PDF)

Figure S10 The rpl14a.L123* mutation truncates the C-terminal **α-helix, which is part of a eukaryote-specific 60S subunit surface**. (**A**) Multiple sequence alignment, generated in the ClustalW output format with T-Coffee, of Rpl14 (L14e) from *S. cerevisiae* (Rpl14a; W303 background), *S. pombe* (Accession: NP_593924) and *H. sapiens* (Accession: NP_003964). Conserved (*), strongly similar (:) and weakly similar (.) amino acids are indicated below the alignment. Secondary structure elements of Rpl14 were taken from the yeast 60S crystal structure (PDB 3U5H and 3U5I; [56]) and the human 60S cryo-EM structure (PDB 3J3B and 3J3F; [57]) or predicted by PSIPRED; α-helices are highlighted in red and β-strands in blue. The position of the rpl14a.L123* mutation, which truncates Rpl14a after amino acid 122, present in sl-mutant strain SL103 is indicated by a blue arrowhead. The portion of the C-terminal α-helix that is missing in the Rpl14a.L123* mutant protein is coloured in orange. The short N-terminal segment of *S*. *cerevisiae* Rpl14, mediating an interaction with Rpl9 (L6) (see panel C), is highlighted in green. (**B**) Position of Rpl14 and its close neighbours, forming a eukaryote-specific 60S subunit surface, on the *S*. *cerevisiae* 60S crystal structure (PDB 3U5H and 3U5I [56]). To facilitate the orientation, the 60S subunit is both shown in its solvent-side (back) view (left part) and when rotated counter-clockwise for 90º along the y-axis (right part); moreover characteristic features of 60S subunits, such as the central protuberance (CP), the P-stalk and the L1-stalk are indicated. The 25S, 5.8S and 5S rRNAs are shown in wheat, pink and lime, respectively. (**C**) Close-up of the eukaryote-specific 60S subunit surface formed by the r-proteins Rpl6 (L6e), Rpl14 (L14e) and Rpl16 (L13) and the rRNA expansion segments ES7L and ES39L. The r-proteins Rpl6, Rpl14 and Rpl16 are shown in blue, red and green, respectively. The rRNA expansion segments ES7L and ES39L are shown in bright orange and salmon. Moreover, r-protein Rpl3 (L3) is shown in yellow and r-protein Rpl9 (L6) is labelled by black letters. The portion of the C-terminal α-helix that is missing in the Rpl14a.L123* mutant protein is coloured in orange.
(PDF)

Figure S11 **Rpl14 and Rpl16 contain strictly eukaryote-specific C-terminal** **α-helices**. (**A**) Multiple sequence alignment, generated in the ClustalW output format with T-Coffee, of Rpl14 (L14e) from *S. cerevisiae* (Rpl14a; W303 background) and L14 from the archaeal organisms *Sulfolobus solfataricus* (Accession: YP_005643206) and *Methanocaldococcus jannaschii* (Accession: NP_247641). Conserved (*), strongly similar (:) and weakly similar (.) amino acids are indicated below the alignment. Secondary structure elements of Rpl14 were taken from the yeast 60S crystal structure (PDB 3U5H and 3U5I; [56]) or predicted by PSIPRED; α-helices are highlighted in red and β-strands in blue. The exclusively eukaryote-specific C-terminal α-helices of Rpl14 are indicated. The portion of the C-terminal α-helix that is missing in the Rpl14a.L123* mutant protein is coloured in orange. Note that L14 is not present in *Haloarcula marismortui*. (**B**) Multiple sequence alignment, generated in the ClustalW output format with T-Coffee, of Rpl16 (L13) from *S*. *cerevisiae* (Rpl16b) and L13 from the archaeal organisms *S*. *solfataricus* (Accession: NP_341641), *M*. *jannaschii* (Accession: NP_247162) and *H*. *marismortui* (Accession: YP_134851). Conserved (*), strongly similar (:) and weakly similar (.) amino acids are indicated below the alignment. Secondary structure elements of Rpl16 and L13 were taken from the yeast 60S crystal structure (PDB 3U5H and 3U5I; [56]) and the *H*. *marismortui* 50S crystal structure (PDB 1VQO; [98]), respectively, or predicted by PSIPRED; α-helices are highlighted in red and β-strands in blue. The exclusively eukaryote-specific C-terminal α-helix of Rpl16 is indicated.
(PDF)

Figure S12 The rpl14a.L123* allele confers a 60S biogenesis defect. Growth phenotype (upper parts) and polysome profile (lower parts) of the rpl14a.L123* mutant in the *∆rpl14a*/*RPL14B* (**A**) and *∆rpl14a*/*∆rpl14b* (**B**) background. Empty vector or plasmid-borne wild-type *RPL14A* and the rpl14a.L123* allele under the control of the authentic promoter were transformed into *RPL14A* shuffle strains containing the genomic *RPL14B* copy (**A**) or carrying a *∆rpl14b* null mutation (**B**). After plasmid shuffling on plates containing 5-FoA, cells were restreaked on YPD plates and then spotted in 10-fold serial dilution steps onto YPD plates, which were incubated for 1.5 d at 30°C, 2 d at 37°C, 2.5 d at 23°C and 4 d at 18°C (upper parts). Cells were grown in YPD medium to an OD_600_ of ~0.8 at 30°C. Cell extracts were prepared under polysome-conserving conditions and eight A_260_ units were resolved in 10-50% sucrose gradients. The absorption profiles were recorded by continuous monitoring at A_254_ (lower parts). Sedimentation is from left to right. The peaks of free 40S and 60S subunits, 80S free couples/monosomes and polysomes are indicated. Half-mers are highlighted by arrowheads. (**C**) Growth phenotype (upper part) and polysome profile (lower part) of the *∆rpl14b* null mutant. The growth of a *RPL14B* and a *∆rpl14b* spore clone, originating from the same complete tetrad of a heterozygous *RPL14B*/*rpl14b*::HIS3MX4 diploid, was assessed by spotting cells in 10-fold serial dilution steps onto YPD plates, which were incubated for 1.5 d at 30°C, 2 d at 37°C, 2.5 d at 23°C and 4 d at 18°C (upper part). *RPL14B* and *∆rpl14b* null mutant cells were grown in YPD medium to an OD_600_ of ~0.8 at 30°C. Preparation of cell extracts as well as analysis and labeling of polysome profiles (lower part) is as described in the legend to Figures S12A and S12B.
(PDF)

Figure S13 Analysis of synthetic enhancement interactions between *rpf1*, *∆nop16* and rpl14a.L123* alleles. *RPF1*/*∆nop16* (**A**), *RPL14A*/*∆nop16* (**B**) and *RPL14A*/*RPF1* (**C**) shuffle or double shuffle strains were co-transformed with plasmids harbouring the indicated wild-type and mutant alleles and/or empty vectors (YCplac111 or YCplac22). Cells were restreaked on SC-Leu-Trp plates and then spotted in 10-fold serial dilution steps onto SC-Leu-Trp and SC+5-FoA-Leu-Trp plates, which were incubated for 3 d or 4 d at 30°C (**A**). In the case of the *RPL14A*/*∆nop16* (**B**) and *RPL14A*/*RPF1* (**C**) shuffle or double shuffle strains, transformed cells were restreaked, after plasmid shuffling on plates containing 5-FoA, on SC-Leu-Trp plates and then spotted in 10-fold serial dilution steps onto SC-Leu-Trp plates, which were incubated for 3 d at 30°C or 4 d at 23°C.
(PDF)

Figure S14 **The conserved and α-helical Ebp2-core domain is sufficient to fulfill the essential Ebp2 function.** (**A**) Multiple sequence alignment, generated in the ClustalW output format with T-Coffee, of Ebp2, excluding its weakly conserved N-terminal extension, from *S. cerevisiae*, *S.* pombe (Accession: NP_593575) and *H. sapiens* (hEBP2 isoform 2; Accession: NP_006815). Conserved (*), strongly similar (:) and weakly similar (.) amino acids are indicated below the alignment. Secondary structure elements were predicted by PSIPRED; α-helices are highlighted in red. Blue arrowheads indicate the N- and C-terminal ends of the different N- and/or C-terminal deletion constructs. (**B**) Growth phenotype of N- and/or C-terminal *ebp2* deletion mutants. Empty vector or plasmid-borne wild-type *EBP2* and the indicated *ebp2* deletion mutants under the control of the authentic promoter were transformed into the *EBP2* shuffle strain. Transformed cells were first restreaked on SC-Leu plates and then spotted in 10-fold serial dilution steps onto SC-Leu and SC+5-FoA-Leu plates, which were incubated for 3 d or 4 d at 30ºC. Schematic representations of Ebp2 and the N- and/or C-terminally truncated Ebp2 variants are depicted on the right. For simplicity, only the predicted α-helices, highlighted in red, within the conserved Ebp2-core domain are indicated.
(PDF)

Figure S15 The ebp2.K287* allele suppresses the lethality of *∆nsa1* null mutant cells. *EBP2*/*NSA1* (**A**), *∆nop16*/*NSA1* (**B**), *RPL14A*/*NSA1* (**C**) and *RPF1*/*NSA1* (**D**) single and double shuffle strains were co-transformed with plasmids harbouring the indicated wild-type and mutant alleles and/or empty vectors (YCplac111 or YCplac22). Transformed cells were restreaked on SC-Leu-Trp plates and then spotted in 10-fold serial dilution steps onto SC-Leu-Trp and SC+5-FoA-Leu-Trp plates, which were incubated for 3 d at 30°C and 4 d at 30°C, respectively.
(PDF)

## References

[1] Henras AK, Soudet J, Gérus M, Lebaron S, Caizergues-Ferrer M et al. (2008) The post-transcriptional steps of eukaryotic ribosome biogenesis. Cell Mol Life Sci 65: 2334-2359. doi:10.1007/s00018-008-8027-0. PubMed: 18408888. 18408888 PMC11131730

[2] Kressler D, Hurt E, Baßler J (2010) Driving ribosome assembly. Biochim Biophys Acta 1803: 673-683. doi:10.1016/j.bbamcr.2009.10.009. PubMed: 19879902. 19879902

[3] Panse VG, Johnson AW (2010) Maturation of eukaryotic ribosomes: acquisition of functionality. Trends Biochem Sci 35: 260-266. doi:10.1016/j.tibs.2010.01.001. PubMed: 20137954. 20137954 PMC2866757

[4] Kressler D, Hurt E, Bergler H, Baßler J (2012) The power of AAA-ATPases on the road of pre-60S ribosome maturation - molecular machines that strip pre-ribosomal particles. Biochim Biophys Acta 1823: 92-100. doi:10.1016/j.bbamcr.2011.06.017. PubMed: 21763358. 21763358 PMC3264779

[5] Martin R, Straub AU, Doebele C, Bohnsack MT (2013) DExD/H-box RNA helicases in ribosome biogenesis. RNA Biol 10: 4-18. doi:10.4161/rna.21879. PubMed: 22922795. 22922795 PMC3590236

[6] Rodríguez-Galán O, García-Gómez JJ, de la Cruz J (2013) Yeast and human RNA helicases involved in ribosome biogenesis: Current status and perspectives. Biochim Biophys Acta 1829: 775-790. doi:10.1016/j.bbagrm.2013.01.007. PubMed: 23357782. 23357782

[7] Strunk BS, Karbstein K (2009) Powering through ribosome assembly. RNA 15: 2083-2104. doi:10.1261/rna.1792109. PubMed: 19850913. 19850913 PMC2779681

[8] Dragon F, Gallagher JE, Compagnone-Post PA, Mitchell BM, Porwancher KA et al. (2002) A large nucleolar U3 ribonucleoprotein required for 18S ribosomal RNA biogenesis. Nature 417: 967-970. doi:10.1038/nature00769. PubMed: 12068309. 12068309 PMC11487672

[9] Grandi P, Rybin V, Baßler J, Petfalski E, Strauß D et al. (2002) 90S pre-ribosomes include the 35S pre-rRNA, the U3 snoRNP, and 40S subunit processing factors but predominantly lack 60S synthesis factors. Mol Cell 10: 105-115. doi:10.1016/S1097-2765(02)00579-8. PubMed: 12150911. 12150911

[10] Osheim YN, French SL, Keck KM, Champion EA, Spasov K et al. (2004) Pre-18S ribosomal RNA is structurally compacted into the SSU processome prior to being cleaved from nascent transcripts in Saccharomyces cerevisiae. Mol Cell 16: 943-954. doi:10.1016/j.molcel.2004.11.031. PubMed: 15610737. 15610737

[11] Decatur WA, Fournier MJ (2003) RNA-guided nucleotide modification of ribosomal and other RNAs. J Biol Chem 278: 695-698. doi:10.1074/jbc.R200023200. PubMed: 12431975. 12431975

[12] Kressler D, Linder P, de la Cruz J (1999) Protein trans-acting factors involved in ribosome biogenesis in *Saccharomyces* *cerevisiae* . Mol Cell Biol 19: 7897-7912. PubMed: 10567516. 10567516 10.1128/mcb.19.12.7897 PMC84875

[13] Piekna-Przybylska D, Decatur WA, Fournier MJ (2007) New bioinformatic tools for analysis of nucleotide modifications in eukaryotic rRNA. RNA 13: 305-312. doi:10.1261/rna.373107. PubMed: 17283215. 17283215 PMC1800513

[14] Venema J, Tollervey D (1999) Ribosome synthesis in *Saccharomyces* *cerevisiae* . Annu Rev Genet 33: 261-311. doi:10.1146/annurev.genet.33.1.261. PubMed: 10690410. 10690410

[15] Decatur WA, Fournier MJ (2002) rRNA modifications and ribosome function. Trends Biochem Sci 27: 344-351. doi:10.1016/S0968-0004(02)02109-6. PubMed: 12114023. 12114023

[16] Pertschy B, Schneider C, Gnädig M, Schäfer T, Tollervey D et al. (2009) RNA helicase Prp43 and its co-factor Pfa1 promote 20 to 18 S rRNA processing catalyzed by the endonuclease Nob1. J Biol Chem 284: 35079-35091. doi:10.1074/jbc.M109.040774. PubMed: 19801658. 19801658 PMC2787369

[17] Karbstein K (2011) Inside the 40S ribosome assembly machinery. Curr Opin Chem Biol 15: 657-663. doi:10.1016/j.cbpa.2011.07.023. PubMed: 21862385. 21862385 PMC3329787

[18] Tschochner H, Hurt E (2003) Pre-ribosomes on the road from the nucleolus to the cytoplasm. Trends Cell Biol 13: 255-263. doi:10.1016/S0962-8924(03)00054-0. PubMed: 12742169. 12742169

[19] Lo KY, Li Z, Bussiere C, Bresson S, Marcotte EM et al. (2010) Defining the pathway of cytoplasmic maturation of the 60S ribosomal subunit. Mol Cell 39: 196-208. doi:10.1016/j.molcel.2010.06.018. PubMed: 20670889. 20670889 PMC2925414

[20] Houseley J, LaCava J, Tollervey D (2006) RNA-quality control by the exosome. Nat Rev Mol Cell Biol 7: 529-539. doi:10.1038/nrm1964. PubMed: 16829983. 16829983

[21] Karbstein K (2013) Quality control mechanisms during ribosome maturation. Trends Cell Biol 23: 242-250. doi:10.1016/j.tcb.2013.01.004. PubMed: 23375955. 23375955 PMC3640646

[22] Lafontaine DL (2010) A 'garbage can' for ribosomes: how eukaryotes degrade their ribosomes. Trends Biochem Sci 35: 267-277. doi:10.1016/j.tibs.2009.12.006. PubMed: 20097077. 20097077

[23] Dez C, Houseley J, Tollervey D (2006) Surveillance of nuclear-restricted pre-ribosomes within a subnucleolar region of Saccharomyces cerevisiae. EMBO J 25: 1534-1546. doi:10.1038/sj.emboj.7601035. PubMed: 16541108. 16541108 PMC1440318

[24] Houseley J, Tollervey D (2006) Yeast Trf5p is a nuclear poly(A) polymerase. EMBO Rep 7: 205-211. doi:10.1038/sj.embor.7400612. PubMed: 16374505. 16374505 PMC1369253

[25] LaCava J, Houseley J, Saveanu C, Petfalski E, Thompson E et al. (2005) RNA degradation by the exosome is promoted by a nuclear polyadenylation complex. Cell 121: 713-724. doi:10.1016/j.cell.2005.04.029. PubMed: 15935758. 15935758

[26] Lebaron S, Schneider C, van Nues RW, Swiatkowska A, Walsh D et al. (2012) Proofreading of pre-40S ribosome maturation by a translation initiation factor and 60S subunits. Nat Struct Mol Biol 19: 744-753. doi:10.1038/nsmb.2308. PubMed: 22751017. 22751017 PMC3654374

[27] Schütz S, Panse VG (2012) Getting ready to commit: ribosomes rehearse translation. Nat Struct Mol Biol 19: 861-862. doi:10.1038/nsmb.2368. PubMed: 22955931. 22955931

[28] Strunk BS, Novak MN, Young CL, Karbstein K (2012) A Translation-Like Cycle Is a Quality Control Checkpoint for Maturing 40S Ribosome Subunits. Cell 150: 111-121. doi:10.1016/j.cell.2012.04.044. PubMed: 22770215. 22770215 PMC3615461

[29] Bussiere C, Hashem Y, Arora S, Frank J, Johnson AW (2012) Integrity of the P-site is probed during maturation of the 60S ribosomal subunit. J Cell Biol 197: 747-759. doi:10.1083/jcb.201112131. PubMed: 22689654. 22689654 PMC3373404

[30] Cole SE, LaRiviere FJ, Merrikh CN, Moore MJ (2009) A convergence of rRNA and mRNA quality control pathways revealed by mechanistic analysis of nonfunctional rRNA decay. Mol Cell 34: 440-450. doi:10.1016/j.molcel.2009.04.017. PubMed: 19481524. 19481524 PMC2712825

[31] Fujii K, Kitabatake M, Sakata T, Miyata A, Ohno M (2009) A role for ubiquitin in the clearance of nonfunctional rRNAs. Genes Dev 23: 963-974. doi:10.1101/gad.1775609. PubMed: 19390089. 19390089 PMC2675866

[32] Hinnebusch AG (2009) Active destruction of defective ribosomes by a ubiquitin ligase involved in DNA repair. Genes Dev 23: 891-895. doi:10.1101/gad.1800509. PubMed: 19390082. 19390082 PMC2763502

[33] LaRiviere FJ, Cole SE, Ferullo DJ, Moore MJ (2006) A late-acting quality control process for mature eukaryotic rRNAs. Mol Cell 24: 619-626. doi:10.1016/j.molcel.2006.10.008. PubMed: 17188037. 17188037

[34] Jankowsky E (2011) RNA helicases at work: binding and rearranging. Trends Biochem Sci 36: 19-29. doi:10.1016/j.tibs.2010.07.008. PubMed: 20813532. 20813532 PMC3017212

[35] Jankowsky E, Bowers H (2006) Remodeling of ribonucleoprotein complexes with DExH/D RNA helicases. Nucleic Acids Res 34: 4181-4188. doi:10.1093/nar/gkl410. PubMed: 16935886. 16935886 PMC1616955

[36] Linder P, Jankowsky E (2011) From unwinding to clamping - the DEAD box RNA helicase family. Nat Rev Mol Cell Biol 12: 505-516. doi:10.1038/nrm3154. PubMed: 21779027. 21779027

[37] Baßler J, Kallas M, Pertschy B, Ulbrich C, Thoms M et al. (2010) The AAA-ATPase Rea1 drives removal of biogenesis factors during multiple stages of 60S ribosome assembly. Mol Cell 38: 712-721. doi:10.1016/j.molcel.2010.05.024. PubMed: 20542003. 20542003 PMC3372891

[38] Kappel L, Loibl M, Zisser G, Klein I, Fruhmann G et al. (2012) Rlp24 activates the AAA-ATPase Drg1 to initiate cytoplasmic pre-60S maturation. J Cell Biol 199: 771-782. doi:10.1083/jcb.201205021. PubMed: 23185031. 23185031 PMC3514788

[39] Kressler D, Roser D, Pertschy B, Hurt E (2008) The AAA ATPase Rix7 powers progression of ribosome biogenesis by stripping Nsa1 from pre-60S particles. J Cell Biol 181: 935-944. doi:10.1083/jcb.200801181. PubMed: 18559667. 18559667 PMC2426938

[40] Ulbrich C, Diepholz M, Bassler J, Kressler D, Pertschy B et al. (2009) Mechanochemical removal of ribosome biogenesis factors from nascent 60S ribosomal subunits. Cell 138: 911-922. doi:10.1016/j.cell.2009.06.045. PubMed: 19737519. 19737519

[41] Jentsch S, Rumpf S (2007) Cdc48 (p97): a "molecular gearbox" in the ubiquitin pathway? Trends Biochem Sci 32: 6-11. doi:10.1016/j.tibs.2006.11.005. PubMed: 17142044. 17142044

[42] Stolz A, Hilt W, Buchberger A, Wolf DH (2011) Cdc48: a power machine in protein degradation. Trends Biochem Sci 36: 515-523. doi:10.1016/j.tibs.2011.06.001. PubMed: 21741246. 21741246

[43] Thomas BJ, Rothstein R (1989) Elevated recombination rates in transcriptionally active DNA. Cell 56: 619-630. doi:10.1016/0092-8674(89)90584-9. PubMed: 2645056. 2645056

[44] Nissan TA, Baßler J, Petfalski E, Tollervey D, Hurt E (2002) 60S pre-ribosome formation viewed from assembly in the nucleolus until export to the cytoplasm. EMBO J 21: 5539-5547. doi:10.1093/emboj/cdf547. PubMed: 12374754. 12374754 PMC129079

[45] James P, Halladay J, Craig EA (1996) Genomic libraries and a host strain designed for highly efficient two-hybrid selection in yeast. Genetics 144: 1425-1436. PubMed: 8978031. 8978031 10.1093/genetics/144.4.1425 PMC1207695

[46] Janke C, Magiera MM, Rathfelder N, Taxis C, Reber S et al. (2004) A versatile toolbox for PCR-based tagging of yeast genes: new fluorescent proteins, more markers and promoter substitution cassettes. Yeast 21: 947-962. doi:10.1002/yea.1142. PubMed: 15334558. 15334558

[47] Longtine MS, McKenzie A 3rd, Demarini DJ, Shah NG, Wach A et al. (1998) Additional modules for versatile and economical PCR-based gene deletion and modification in *Saccharomyces* *cerevisiae* . Yeast 14: 953-961. doi:10.1002/(SICI)1097-0061(199807)14:10. PubMed: 9717241. 9717241

[48] Puig O, Caspary F, Rigaut G, Rutz B, Bouveret E et al. (2001) The tandem affinity purification (TAP) method: a general procedure of protein complex purification. Methods 24: 218-229. doi:10.1006/meth.2001.1183. PubMed: 11403571. 11403571

[49] Kressler D, Doère M, Rojo M, Linder P (1999) Synthetic lethality with conditional *dbp6* alleles identifies Rsa1p, a nucleoplasmic protein involved in the assembly of 60S ribosomal subunits. Mol Cell Biol 19: 8633-8645. PubMed: 10567587. 10567587 10.1128/mcb.19.12.8633 PMC85000

[50] de la Cruz J, Iost I, Kressler D, Linder P (1997) The p20 and Ded1 proteins have antagonistic roles in eIF4E-dependent translation in Saccharomyces cerevisiae. Proc Natl Acad Sci U S A 94: 5201-5206. doi:10.1073/pnas.94.10.5201. PubMed: 9144215. 9144215 PMC24656

[51] Kressler D, de la Cruz J, Rojo M, Linder P (1997) Fal1p is an essential DEAD-box protein involved in 40S-ribosomal-subunit biogenesis in *Saccharomyces* *cerevisiae* . Mol Cell Biol 17: 7283-7294. PubMed: 9372960. 9372960 10.1128/mcb.17.12.7283 PMC232585

[52] Yaffe MP, Schatz G (1984) Two nuclear mutations that block mitochondrial protein import in yeast. Proc Natl Acad Sci U S A 81: 4819-4823. doi:10.1073/pnas.81.15.4819. PubMed: 6235522. 6235522 PMC391582

[53] Simos G, Segref A, Fasiolo F, Hellmuth K, Shevchenko A et al. (1996) The yeast protein Arc1p binds to tRNA and functions as a cofactor for the methionyl- and glutamyl-tRNA synthetases. EMBO J 15: 5437-5448. PubMed: 8895587. 8895587 PMC452286

[54] Notredame C, Higgins DG, Heringa J (2000) T-Coffee: A novel method for fast and accurate multiple sequence alignment. J Mol Biol 302: 205-217. doi:10.1006/jmbi.2000.4042. PubMed: 10964570. 10964570

[55] Jones DT (1999) Protein secondary structure prediction based on position-specific scoring matrices. J Mol Biol 292: 195-202. doi:10.1006/jmbi.1999.3091. PubMed: 10493868. 10493868

[56] Ben-Shem A, Garreau de Loubresse N, Melnikov S, Jenner L, Yusupova G et al. (2011) The structure of the eukaryotic ribosome at 3.0 Å resolution. Science 334: 1524-1529. doi:10.1126/science.1212642. PubMed: 22096102. 22096102

[57] Anger AM, Armache JP, Berninghausen O, Habeck M, Subklewe M et al. (2013) Structures of the human and Drosophila 80S ribosome. Nature 497: 80-85. doi:10.1038/nature12104. PubMed: 23636399. 23636399

[58] Zagulski M, Kressler D, Bécam AM, Rytka J, Herbert CJ (2003) Mak5p, which is required for the maintenance of the M1 dsRNA virus, is encoded by the yeast ORF YBR142w and is involved in the biogenesis of the 60S subunit of the ribosome. Mol Genet Genomics 270: 216-224. doi:10.1007/s00438-003-0913-4. PubMed: 13680366. 13680366

[59] Bergès T, Petfalski E, Tollervey D, Hurt EC (1994) Synthetic lethality with fibrillarin identifies NOP77p, a nucleolar protein required for pre-rRNA processing and modification. EMBO J 13: 3136-3148. PubMed: 8039506. 8039506 10.1002/j.1460-2075.1994.tb06612.x PMC395205

[60] Granneman S, Petfalski E, Tollervey D (2011) A cluster of ribosome synthesis factors regulate pre-rRNA folding and 5.8S rRNA maturation by the Rat1 exonuclease. EMBO J 30: 4006-4019. doi:10.1038/emboj.2011.256. PubMed: 21811236. 21811236 PMC3209772

[61] Sun C, Woolford JL Jr. (1994) The yeast NOP4 gene product is an essential nucleolar protein required for pre-rRNA processing and accumulation of 60S ribosomal subunits. EMBO J 13: 3127-3135. PubMed: 8039505. 8039505 10.1002/j.1460-2075.1994.tb06611.x PMC395203

[62] Galardi S, Fatica A, Bachi A, Scaloni A, Presutti C et al. (2002) Purified box C/D snoRNPs are able to reproduce site-specific 2'-O-methylation of target RNA in vitro. Mol Cell Biol 22: 6663-6668. doi:10.1128/MCB.22.19.6663-6668.2002. PubMed: 12215523. 12215523 PMC134041

[63] Lin J, Lai S, Jia R, Xu A, Zhang L et al. (2011) Structural basis for site-specific ribose methylation by box C/D RNA protein complexes. Nature 469: 559-563. doi:10.1038/nature09688. PubMed: 21270896. 21270896

[64] Niewmierzycka A, Clarke S (1999) S-Adenosylmethionine-dependent methylation in Saccharomyces cerevisiae. Identification of a novel protein arginine methyltransferase. J Biol Chem 274: 814-824. doi:10.1074/jbc.274.2.814. PubMed: 9873020. 9873020

[65] Tollervey D, Lehtonen H, Jansen R, Kern H, Hurt EC (1993) Temperature-sensitive mutations demonstrate roles for yeast fibrillarin in pre-rRNA processing, pre-rRNA methylation, and ribosome assembly. Cell 72: 443-457. doi:10.1016/0092-8674(93)90120-F. PubMed: 8431947. 8431947

[66] Wang H, Boisvert D, Kim KK, Kim R, Kim SH (2000) Crystal structure of a fibrillarin homologue from Methanococcus jannaschii, a hyperthermophile, at 1.6 A resolution. EMBO J 19: 317-323. doi:10.1093/emboj/19.3.317. PubMed: 10654930. 10654930 PMC305568

[67] Hanson PI, Whiteheart SW (2005) AAA+ proteins: have engine, will work. Nat Rev Mol Cell Biol 6: 519-529. doi:10.1038/nrg1649. PubMed: 16072036. 16072036

[68] Esaki M, Ogura T (2010) ATP-bound form of the D1 AAA domain inhibits an essential function of Cdc48p/p97. Biochem Cell Biol 88: 109-117. doi:10.1139/O09-116. PubMed: 20130684. 20130684

[69] Ye Y, Meyer HH, Rapoport TA (2003) Function of the p97-Ufd1-Npl4 complex in retrotranslocation from the ER to the cytosol: dual recognition of nonubiquitinated polypeptide segments and polyubiquitin chains. J Cell Biol 162: 71-84. doi:10.1083/jcb.200302169. PubMed: 12847084. 12847084 PMC2172719

[70] Dez C, Froment C, Noaillac-Depeyre J, Monsarrat B, Caizergues-Ferrer M et al. (2004) Npa1p, a component of very early pre-60S ribosomal particles, associates with a subset of small nucleolar RNPs required for peptidyl transferase center modification. Mol Cell Biol 24: 6324-6337. doi:10.1128/MCB.24.14.6324-6337.2004. PubMed: 15226434. 15226434 PMC434229

[71] Gavin AC, Aloy P, Grandi P, Krause R, Boesche M et al. (2006) Proteome survey reveals modularity of the yeast cell machinery. Nature 440: 631-636. doi:10.1038/nature04532. PubMed: 16429126. 16429126

[72] Gavin AC, Bösche M, Krause R, Grandi P, Marzioch M et al. (2002) Functional organization of the yeast proteome by systematic analysis of protein complexes. Nature 415: 141-147. doi:10.1038/415141a. PubMed: 11805826. 11805826

[73] Shimoji K, Jakovljevic J, Tsuchihashi K, Umeki Y, Wan K et al. (2012) Ebp2 and Brx1 function cooperatively in 60S ribosomal subunit assembly in Saccharomyces cerevisiae. Nucleic Acids Res 40: 4574-4588. doi:10.1093/nar/gks057. PubMed: 22319211. 22319211 PMC3378894

[74] Fatica A, Cronshaw AD, Dlakić M, Tollervey D (2002) Ssf1p prevents premature processing of an early pre-60S ribosomal particle. Mol Cell 9: 341-351. doi:10.1016/S1097-2765(02)00458-6. PubMed: 11864607. 11864607

[75] Alexandrov A, Colognori D, Steitz JA (2011) Human eIF4AIII interacts with an eIF4G-like partner, NOM1, revealing an evolutionarily conserved function outside the exon junction complex. Genes Dev 25: 1078-1090. doi:10.1101/gad.2045411. PubMed: 21576267. 21576267 PMC3093123

[76] Granneman S, Lin C, Champion EA, Nandineni MR, Zorca C et al. (2006) The nucleolar protein Esf2 interacts directly with the DExD/H box RNA helicase, Dbp8, to stimulate ATP hydrolysis. Nucleic Acids Res 34: 3189-3199. doi:10.1093/nar/gkl419. PubMed: 16772403. 16772403 PMC1483223

[77] Kossen K, Karginov FV, Uhlenbeck OC (2002) The carboxy-terminal domain of the DExDH protein YxiN is sufficient to confer specificity for 23S rRNA. J Mol Biol 324: 625-636. doi:10.1016/S0022-2836(02)01140-3. PubMed: 12460566. 12460566

[78] Lebaron S, Papin C, Capeyrou R, Chen YL, Froment C et al. (2009) The ATPase and helicase activities of Prp43p are stimulated by the G-patch protein Pfa1p during yeast ribosome biogenesis. EMBO J 28: 3808-3819. doi:10.1038/emboj.2009.335. PubMed: 19927118. 19927118 PMC2797057

[79] Marintchev A, Edmonds KA, Marintcheva B, Hendrickson E, Oberer M et al. (2009) Topology and regulation of the human eIF4A/4G/4H helicase complex in translation initiation. Cell 136: 447-460. doi:10.1016/j.cell.2009.01.014. PubMed: 19203580. 19203580 PMC2656774

[80] Walbott H, Mouffok S, Capeyrou R, Lebaron S, Humbert O et al. (2010) Prp43p contains a processive helicase structural architecture with a specific regulatory domain. EMBO J 29: 2194-2204. doi:10.1038/emboj.2010.102. PubMed: 20512115. 20512115 PMC2905241

[81] Young CL, Khoshnevis S, Karbstein K (2013) Cofactor-dependent specificity of a DEAD-box protein. Proc Natl Acad Sci U S A 110: E2668-E2676. doi:10.1073/pnas.1302577110. PubMed: 23630256. 23630256 PMC3718167

[82] Harnpicharnchai P, Jakovljevic J, Horsey E, Miles T, Roman J et al. (2001) Composition and functional characterization of yeast 66S ribosome assembly intermediates. Mol Cell 8: 505-515. doi:10.1016/S1097-2765(01)00344-6. PubMed: 11583614. 11583614

[83] Huber MD, Dworet JH, Shire K, Frappier L, McAlear MA (2000) The budding yeast homolog of the human EBNA1-binding protein 2 (Ebp2p) is an essential nucleolar protein required for pre-rRNA processing. J Biol Chem 275: 28764-28773. doi:10.1074/jbc.M000594200. PubMed: 10849420. 10849420

[84] Tsujii R, Miyoshi K, Tsuno A, Matsui Y, Toh-e A, et al (2000) Ebp2p, yeast homologue of a human protein that interacts with Epstein-Barr virus nuclear antigen 1, is required for pre-rRNA processing and ribosomal subunit assembly. Genes Cells 5: 543-553. 10947841 10.1046/j.1365-2443.2000.00346.x

[85] Wehner KA, Baserga SJ (2002) The sigma(70)-like motif: a eukaryotic RNA binding domain unique to a superfamily of proteins required for ribosome biogenesis. Mol Cell 9: 329-339. doi:10.1016/S1097-2765(02)00438-0. PubMed: 11864606. 11864606

[86] Jenner L, Melnikov S, de Loubresse NG, Ben-Shem A, Iskakova M et al. (2012) Crystal structure of the 80S yeast ribosome. Curr Opin Struct Biol 22: 759-767. doi:10.1016/j.sbi.2012.07.013. PubMed: 22884264. 22884264

[87] Ban N, Nissen P, Hansen J, Moore PB, Steitz TA (2000) The complete atomic structure of the large ribosomal subunit at 2.4 A resolution. Science 289: 905-920. doi:10.1126/science.289.5481.905. PubMed: 10937989. 10937989

[88] Steffen KK, McCormick MA, Pham KM, MacKay VL, Delaney JR et al. (2012) Ribosome deficiency protects against ER stress in Saccharomyces cerevisiae. Genetics 191: 107-118. doi:10.1534/genetics.111.136549. PubMed: 22377630. 22377630 PMC3338253

[89] Lebreton A, Rousselle JC, Lenormand P, Namane A, Jacquier A et al. (2008) 60S ribosomal subunit assembly dynamics defined by semi-quantitative mass spectrometry of purified complexes. Nucleic Acids Res 36: 4988-4999. doi:10.1093/nar/gkn469. PubMed: 18658244. 18658244 PMC2528192

[90] Kaser A, Bogengruber E, Hallegger M, Doppler E, Lepperdinger G et al. (2001) Brix from xenopus laevis and Brx1p from yeast define a new family of proteins involved in the biogenesis of large ribosomal subunits. Biol Chem 382: 1637-1647. PubMed: 11843177. 11843177 10.1515/BC.2001.199

[91] Kruiswijk T, Planta RJ, Krop JM (1978) The course of the assembly of ribosomal subunits in yeast. Biochim Biophys Acta 517: 378-389. doi:10.1016/0005-2787(78)90204-6. PubMed: 626744. 626744

[92] Baßler J, Grandi P, Gadal O, Leßmann T, Petfalski E et al. (2001) Identification of a 60S preribosomal particle that is closely linked to nuclear export. Mol Cell 8: 517-529. doi:10.1016/S1097-2765(01)00342-2. PubMed: 11583615. 11583615

[93] de la Cruz J, Lacombe T, Deloche O, Linder P, Kressler D (2004) The putative RNA helicase Dbp6p functionally interacts with Rpl3p, Nop8p and the novel trans-acting Factor Rsa3p during biogenesis of 60S ribosomal subunits in *Saccharomyces* *cerevisiae* . Genetics 166: 1687-1699. doi:10.1534/genetics.166.4.1687. PubMed: 15126390. 15126390 PMC1470830

[94] Rosado IV, Dez C, Lebaron S, Caizergues-Ferrer M, Henry Y et al. (2007) Characterization of Saccharomyces cerevisiae Npa2p (Urb2p) reveals a low-molecular-mass complex containing Dbp6p, Npa1p (Urb1p), Nop8p, and Rsa3p involved in early steps of 60S ribosomal subunit biogenesis. Mol Cell Biol 27: 1207-1221. doi:10.1128/MCB.01523-06. PubMed: 17145778. 17145778 PMC1800719

[95] Mulder AM, Yoshioka C, Beck AH, Bunner AE, Milligan RA et al. (2010) Visualizing ribosome biogenesis: parallel assembly pathways for the 30S subunit. Science 330: 673-677. doi:10.1126/science.1193220. PubMed: 21030658. 21030658 PMC2990404

[96] Talkington MW, Siuzdak G, Williamson JR (2005) An assembly landscape for the 30S ribosomal subunit. Nature 438: 628-632. doi:10.1038/nature04261. PubMed: 16319883. 16319883 PMC1444899

[97] Gadal O, Strauß D, Braspenning J, Hoepfner D, Petfalski E et al. (2001) A nuclear AAA-type ATPase (Rix7p) is required for biogenesis and nuclear export of 60S ribosomal subunits. EMBO J 20: 3695-3704. doi:10.1093/emboj/20.14.3695. PubMed: 11447111. 11447111 PMC125552

[98] Schmeing TM, Huang KS, Kitchen DE, Strobel SA, Steitz TA (2005) Structural insights into the roles of water and the 2' hydroxyl of the P site tRNA in the peptidyl transferase reaction. Mol Cell 20: 437-448. doi:10.1016/j.molcel.2005.09.006. PubMed: 16285925. 16285925

