# Supplementary material for: Mak5 and Ebp2 Act Together on Early Pre-60S Particles and Their Reduced Functionality Bypasses the Requirement for the Essential Pre-60S Factor Nsa1
Source: PLoS One. 2013 Dec 2;8(12):e82741. doi: 10.1371/journal.pone.0082741 (PMC3846774; doi:10.1371/journal.pone.0082741)
Supplement: Table S1 — Yeast strains used in this study. (PDF) [file pone.0082741.s001.pdf]

Supporting Table S1. Yeast strains used in this study.

Name		Relevant genotype	Source
Y4138	*MAT***a**	*rix7*::kanMX4 *ade3*::kanMX4 pHT4467Δ-*RIX7*	Kressler *et al*. 2008
Y3274	*MAT*α	*nsa1*::kanMX4 *ade3*::kanMX4 YCplac33-*NSA1*	This study
Y3900	*MAT***a**	*nsa1*::kanMX4 *ade3*::kanMX4 pHT4467Δ-*NSA1*	Kressler *et al*. 2008
Y3903	*MAT*α	*nsa1*::kanMX4 *ade3*::kanMX4 pHT4467Δ-*NSA1*	This study
YDK11-5A	*MAT*α	*ade3*::kanMX4	Kressler *et al*. 1999
YHD60	*MAT***a**	*nsa1*::kanMX4 *ade3*::kanMX4 *mak5-S5* YCplac33-*NSA1*	This study
YDP1	*MAT*α	*mak5*::natNT2 *ade3*::kanMX4 pHT4467Δ-*MAK5*	This study
YDP4	*MAT***a**	*mak5*::natNT2 *ade3*::kanMX4 pHT4467Δ-*MAK5*	This study
YDP264	*MAT***a**	*nop1*::HIS3MX4 YCplac33-*NOP1*	This study
YDP9	*MAT***a**	*nop4*::HIS3MX4 YCplac33-*NOP4*	This study
YKL481	*MAT***a**	*nop1*::natNT2 YCplac33-*NOP1*<br>*nop4*::HIS3MX4 YCplac33-*NOP4*	This study
YDP22	*MAT***a**	*nsa1*::kanMX4 YCplac33-*NSA1*<br>*mak5*::natNT2 YCplac33-*MAK5*	This study
YKL447	*MAT***a**	*nsa1*::kanMX4 YCplac33-*NSA1*<br>*nop1*::HIS3MX4 YCplac33-*NOP1*	This study
YDP13	*MAT*α	*nsa1*::kanMX4 YCplac33-*NSA1*<br>*nop4*::HIS3MX4 YCplac33-*NOP4*	This study
YKL409	*MAT***a**	*ebp2*::HIS3MX4 *ade3*::kanMX4 pHT4467Δ-*EBP2*	This study
YUS53	*MAT***a**	*rpf1*::HIS3MX4 YCplac33-*RPF1*	This study
YUS115	*MAT*α	*NOP16*	This study
YUS116	*MAT***a**	*nop16*::natNT2	This study
YUS117	*MAT*α	*nop16*::natNT2	This study
YUS118	*MAT***a**	*NOP16*	This study
YUS90	*MAT***a**	*rpl14a*::natNT2 YCplac33-*RPL14A*	This study
YUS171	*MAT***a**	*rpl14a*::natNT2 *rpl14b*::HIS3MX4 YCplac33-*RPL14A*	This study
YUS33	*MAT***a**	*rpl14b*::HIS3MX4	This study
YUS34	*MAT***a**	*RPL14B*	This study
YKL508	*MAT***a**	*mak5*::natNT2 YCplac33-*MAK5*<br>*ebp2*::HIS3MX4 YCplac33-*EBP2*	This study
YUS126	*MAT*α	*mak5*::natNT2 *nop16*::HIS3MX4 YCplac33-*MAK5*	This study
YUS108	*MAT***a**	*mak5*::natNT2 YCplac33-*MAK5*<br>*rpf1*::HIS3MX4 YCplac33-*RPF1*	This study
YUS168	*MAT*α	*mak5*::natNT2 YCplac33-*MAK5*<br>*rpl14a*::HIS3MX4 YCplac33-*RPL14A*	This study
YUS144	*MAT*α	*ebp2*::HIS3MX4 *nop16*::natNT2 pHT4467Δ-*EBP2*	This study
YUS149	*MAT*α	*ebp2*::HIS3MX4 pHT4467Δ-*EBP2*<br>*rpf1*::natNT2 YCplac33-*RPF1*	This study
YKL503	*MAT*α	*ebp2*::HIS3MX4 YCplac33-*EBP2*<br>*rpl14a*::natNT2 YCplac33-*RPL14A*	This study
YUS131	*MAT***a**	*rpf1*::natNT2 *nop16*::HIS3MX4 YCplac33-*RPF1*	This study
YUS165	*MAT*α	*rpl14a*::natNT2 *nop16*::HIS3MX4 YCplac33-*RPL14A*	This study
YUS280	*MAT***a**	*rpl14a*::natNT2 YCplac33-*RPL14A*<br>*rpf1*::HIS3MX4 YCplac33-*RPF1*	This study

Supporting Table S1 continued. Yeast strains used in this study.

Name		Relevant genotype	Source
YKL452	*MAT***a**	*nsa1*::kanMX4 YCplac33-*NSA1* *ebp2*::HIS3MX4 pHT4467Δ-*EBP2*	This study
YKL485	*MAT***a**	*nsa1*::kanMX4 YCplac33-*NSA1* *ebp2*::HIS3MX4 YCplac33-*EBP2*	This study
YKL432	*MAT***a**	*nsa1*::kanMX4 *nop16*::HIS3MX4 YCplac33-*NSA1*	This study
YKL440	*MAT***a**	*nsa1*::kanMX4 YCplac33-*NSA1* *rpf1*::HIS3MX4 YCplac33-*RPF1*	This study
YKL442	*MAT***a**	*nsa1*::kanMX4 YCplac33-*NSA1* *rpl14a*::HIS3MX4 YCplac33-*RPL14A*	This study
YDP229*	*MAT*α	*NOP7*-TAP::kl*TRP1 MAK5*-GFP::HIS3MX4	This study
YDP236*	*MAT*α	*SSF1*-TAP::kl*TRP1 MAK5*-GFP::HIS3MX4	This study
YDP239*	*MAT*α	*NSA1*-TAP::kl*TRP1 MAK5*-GFP::HIS3MX4	This study
YDP237*	*MAT*α	*RIX1*-TAP::kl*TRP1 MAK5*-GFP::HIS3MX4	This study
YDP233*	*MAT*α	*ARX1*-TAP::kl*TRP1 MAK5*-GFP::HIS3MX4	This study
YDP231*	*MAT*α	*LSG1*-TAP::kl*TRP1 MAK5*-GFP::HIS3MX4	This study

Strains used in this study were either derived from W303 (*MAT***a**/*MAT*α *ade2-1/ade2-1 his3-11,15/his3-11,15 leu2-3,112/leu2-3,112 trp1-1/trp1-1 ura3-1/ura3-1 can1-100/can1-100*) or from DS1-2b (*MAT*α *his3-Δ200 leu2-Δ1 trp1-Δ63 ura3-52*), the latter are marked by an asterisk.
