# Supplementary material for: Mak5 and Ebp2 Act Together on Early Pre-60S Particles and Their Reduced Functionality Bypasses the Requirement for the Essential Pre-60S Factor Nsa1
Source: PLoS One. 2013 Dec 2;8(12):e82741. doi: 10.1371/journal.pone.0082741 (PMC3846774; doi:10.1371/journal.pone.0082741)
Supplement: Table S2 — Plasmids used in this study. (PDF) [file pone.0082741.s002.pdf]

Supporting Table S2. Plasmids used in this study*.

Name	Relevant information	Source
pHT4467Δ-*MAK5*	CEN6 (instable), *URA3*, *ADE3*	This study
YCplac33-*MAK5*	CEN, *URA3*, P*MAK5*, T*ADH1*	This study
YCplac111-*MAK5*	CEN, *LEU2*, P*MAK5*, T*ADH1*	This study
YCplac22-*MAK5*	CEN, *TRP1*, P*MAK5*, T*ADH1*	This study
pNTAPF111-*MAK5*	CEN, *LEU2*, P*MAK5*, T*ADH1*, N-terminal TAP/Flag tag	This study
pHT4467Δ-*NSA1*	CEN6 (instable), *URA3*, *ADE3*	Kressler *et al*. 2008
YCplac33-*NSA1*	CEN, *URA3*	Kressler *et al*. 2008
YCplac111-*NSA1*	CEN, *LEU2*	Kressler *et al*. 2008
YCplac22-*NSA1*	CEN, *TRP1*	Kressler *et al*. 2008
pADH111-hNSA1.N328	CEN, *LEU2*, P*ADH1*, T*ADH1*	This study
YCplac33-*NOP1*	CEN, *URA3*, P*NOP1*, T*ADH1*	This study
YCplac111-*NOP1*	CEN, *LEU2*, P*NOP1*, T*ADH1*	This study
YCplac33-*NOP4*	CEN, *URA3*, P*NOP4*, T*ADH1*	This study
YCplac111-*NOP4*	CEN, *LEU2*, P*NOP4*, T*ADH1*	This study
YCplac22-*NOP4*	CEN, *TRP1*, P*NOP4*, T*ADH1*	This study
pHT4467Δ-*RIX7*	CEN6 (instable), *URA3*, *ADE3*	Kressler *et al*. 2008
YCplac111-*RIX7*	CEN, *LEU2*, P*RIX7*, T*ADH1*	Kressler *et al*. 2008
pTAPC111-*RIX7*	CEN, *LEU2*, P*RIX7*, T*CYC1*, C-terminal TAP tag	Kressler *et al*. 2008
pGTAPC111-*RIX7*	CEN, *LEU2*, P*GAL1-10*, T*CYC1*, C-terminal TAP tag	This study
pHT4467Δ-*EBP2*	CEN6 (instable), *URA3*, *ADE3*	This study
YCplac33-*EBP2*	CEN, *URA3*, P*EBP2*, T*ADH1*	This study
YCplac111-*EBP2*	CEN, *LEU2*, P*EBP2*, T*ADH1*	This study
YCplac22-*EBP2*	CEN, *TRP1*, P*EBP2*, T*ADH1*	This study
YCplac111-*NOP16*	CEN, *LEU2*, P*NOP16*, T*ADH1*	This study
YCplac22-*NOP16*	CEN, *TRP1*, P*NOP16*, T*ADH1*	This study
YCplac33-*RPF1*	CEN, *URA3*, P*RPF1*, T*ADH1*	This study
YCplac111-*RPF1*	CEN, *LEU2*, P*RPF1*, T*ADH1*	This study
YCplac22-*RPF1*	CEN, *TRP1*, P*RPF1*, T*ADH1*	This study
YCplac33-*RPL14A*	CEN, *URA3*, P*RPL14A*, T*RPL14A*	This study
YCplac111-*RPL14A*	CEN, *LEU2*, P*RPL14A*, T*RPL14A*	This study
YCplac22-*RPL14A*	CEN, *TRP1*, P*RPL14A*, T*RPL14A*	This study
pFA6a-HIS3MX4	for genomic deletion disruption	Longtine *et al*. 1998
pFA6a-natNT2	for genomic deletion disruption	Janke *et al*. 2004
pFA6a-GFP-HIS3MX4	GFP(S65T), T*ADH1*; for genomic C-terminal tagging	Longtine *et al*. 1998
pBS1479	TAP tag; for genomic C-terminal tagging	Puig *et al*. 2001

*For simplicity, only the plasmids containing the respective wild-type genes are listed. The mutant variants thereof used in this study were cloned into the listed plasmids. P and T denote promoter and terminator, respectively.
