# Supplementary figures and images for: Mak5 and Ebp2 Act Together on Early Pre-60S Particles and Their Reduced Functionality Bypasses the Requirement for the Essential Pre-60S Factor Nsa1
Source: PLoS One. 2013 Dec 2;8(12):e82741. doi: 10.1371/journal.pone.0082741 (PMC3846774; doi:10.1371/journal.pone.0082741)

Supporting Figure S1

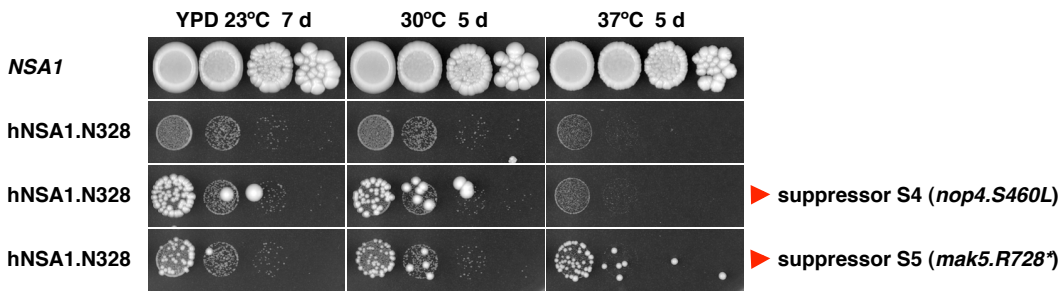

Supplement: Figure S1 — Isolation of spontaneous bypass suppressors of the lethal ∆nsa1 null mutant phenotype. The NSA1 shuffle strain was transformed with plasmids carrying NSA1 under the control of the authentic promoter or human NSA1, encoding only the predicted WD-40 β-propeller (hNSA1.N328), under the control of the strong ADH1 promoter. After plasmid shuffling on plates containing 5-FoA, cells were restreaked on YPD plates and then spotted in 10-fold serial dilution steps onto YPD plates, which were incubated for 7 d at 23°C, 5 d at 30°C and 5 d at 37°C. While hNSA1.N328 complements very weakly the lethality of ∆nsa1 null mutant cells, spontaneous suppressors of this slow-growth phenotype arise with high frequency. Subsequent cloning and allele sequencing revealed that suppressor strains S4 and S5 contain the nop4.S460L and mak5.R728* alleles, respectively. (PDF) [file pone.0082741.s003.pdf]

Supporting Figure S2

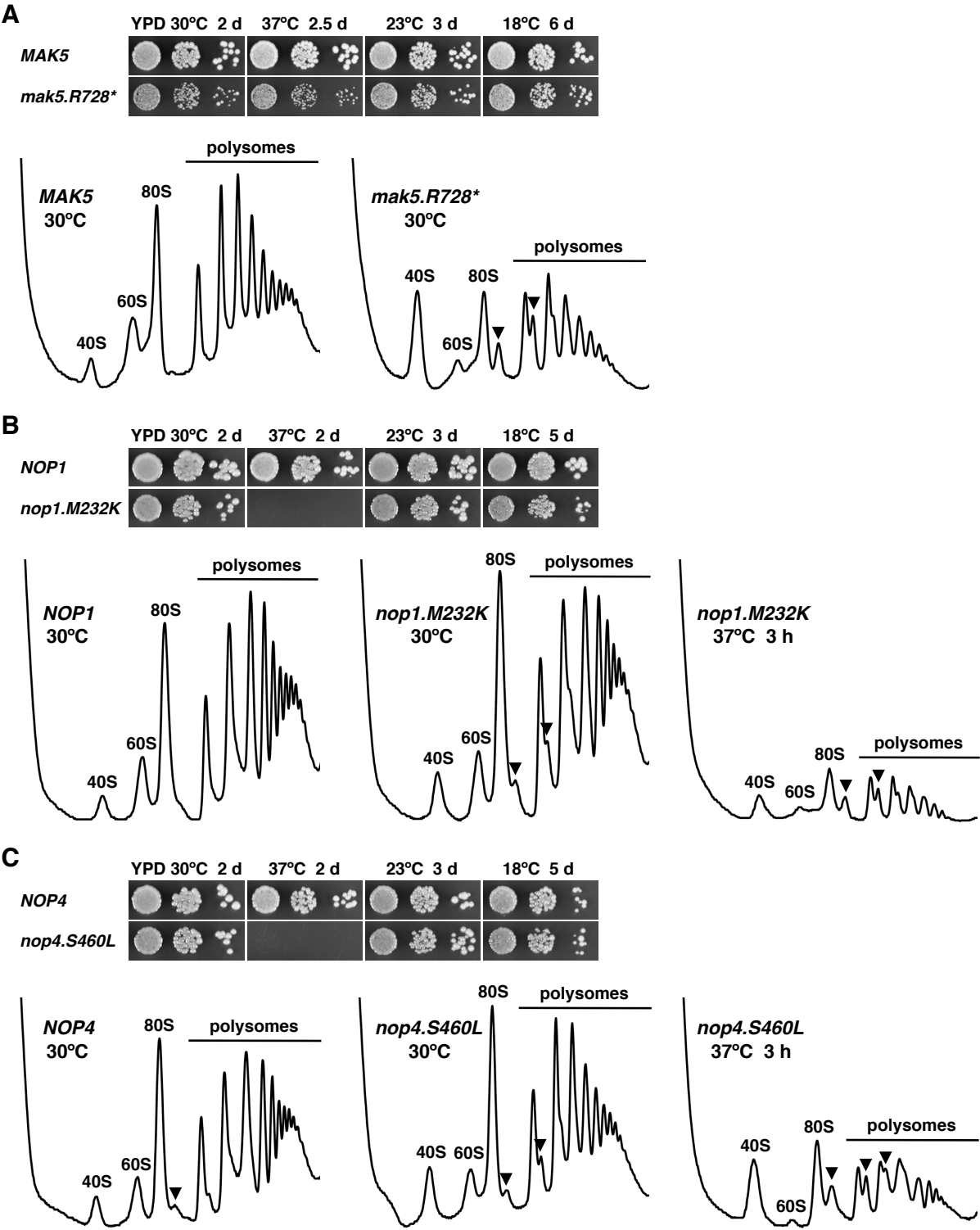

Supplement: Figure S2 — Growth and polysome profile analyses of ∆nsa1 suppressor alleles. MAK5 (A), NOP1 (B) and NOP4 (C) shuffle strains were transformed with plasmids harbouring, under the control of the authentic promoters, either the MAK5, NOP1 or NOP4 wild-type genes or the mak5.R728*, nop1.M232K or nop4.S460L mutant alleles, respectively. After plasmid shuffling on plates containing 5-FoA, cells were restreaked on YPD plates and then spotted in 10-fold serial dilution steps onto YPD plates, which were incubated for the indicated times at 30°C, 37°C, 23°C and 18°C (upper parts). Polysome profiles of the above wild-type and mutant strains are shown in the lower parts of each subfigure. Briefly, cells were grown in YPD medium to an OD600 of ~0.8 at 30°C or shifted for 3 h to 37°C. Cell extracts were prepared under polysome-conserving conditions and eight A260 units were resolved in 10-50% sucrose gradients. The absorption profiles were recorded by continuous monitoring at A254. Sedimentation is from left to right. The peaks of free 40S and 60S subunits, 80S free couples/monosomes and polysomes are indicated. Half-mers are highlighted by arrowheads. The polysome profiles of NOP1 and NOP4 wild-type strains shifted for 3 h to 37°C are very similar to the ones obtained at 30°C and have been therefore omitted to increase the clarity of the Figure. (PDF) [file pone.0082741.s004.pdf]

Supporting Figure S3

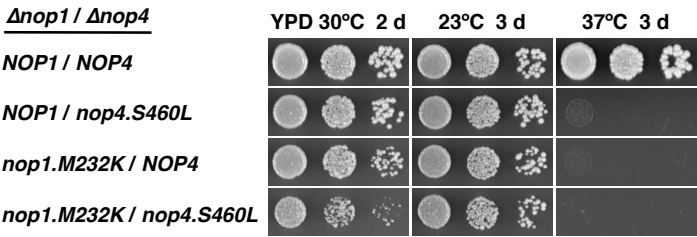

Supplement: Figure S3 — The nop1.M232K and nop4.S460L alleles synergistically affect growth. The NOP1/NOP4 double shuffle strain was co-transformed with plasmids harbouring wild-type NOP1 or the nop1.M232K allele and wild-type NOP4 or the nop4.S460L allele. Cells were restreaked, after plasmid shuffling on plates containing 5-FoA, on YPD plates and then spotted in 10-fold serial dilution steps onto YPD plates, which were incubated for 2 d at 30°C, 3 d at 23°C and 3 d at 37°C. (PDF) [file pone.0082741.s005.pdf]

Supporting Figure S4

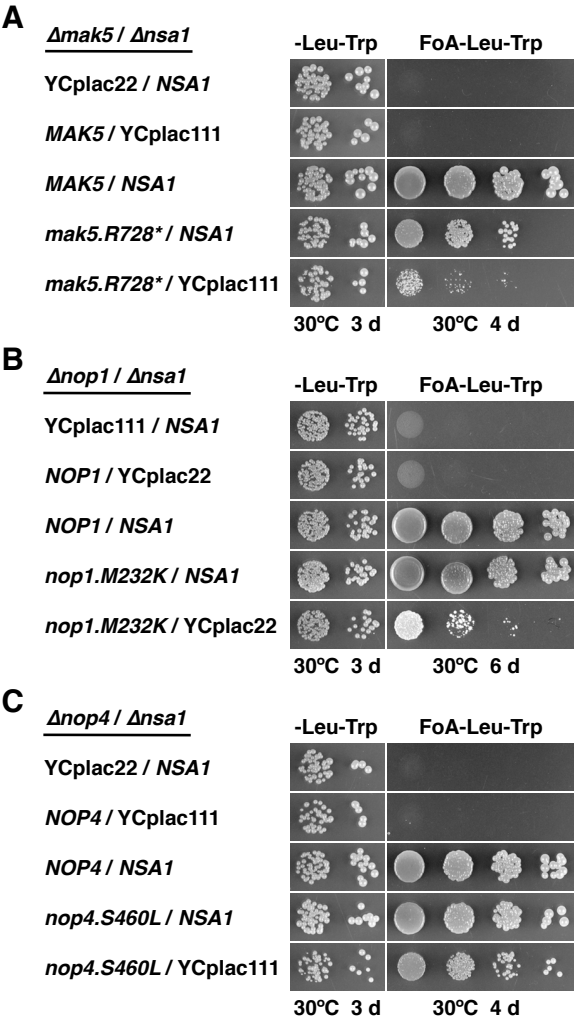

Supplement: Figure S4 — The mak5.R728*, nop1.M232K and nop4.S460L alleles suppress the lethality of ∆nsa1 null mutant cells. MAK5/NSA1 (A), NOP1/NSA1 (B) and NOP4/NSA1 (C) double shuffle strains were co-transformed with plasmids harbouring the indicated wild-type and mutant alleles and/or empty vectors (YCplac111 or YCplac22). Transformed cells were restreaked on SC-Leu-Trp plates and then spotted in 10-fold serial dilution steps onto SC-Leu-Trp and SC+5-FoA-Leu-Trp plates, which were incubated for 3 d at 30°C and 4 d or 6 d at 30°C, respectively. (PDF) [file pone.0082741.s006.pdf]

Supporting Figure S5

A

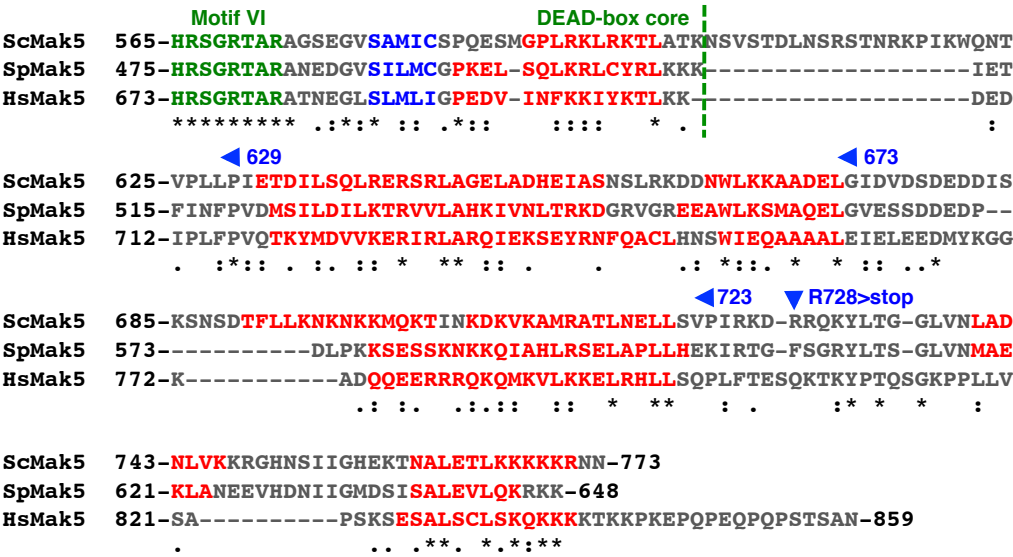

B

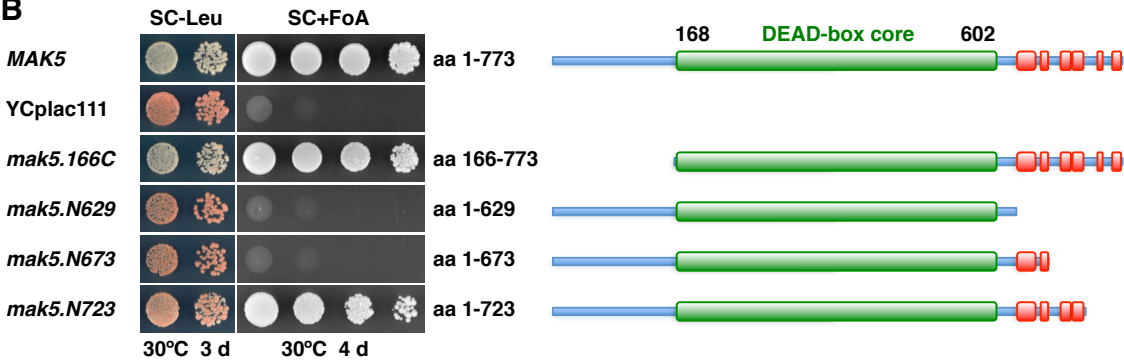

C

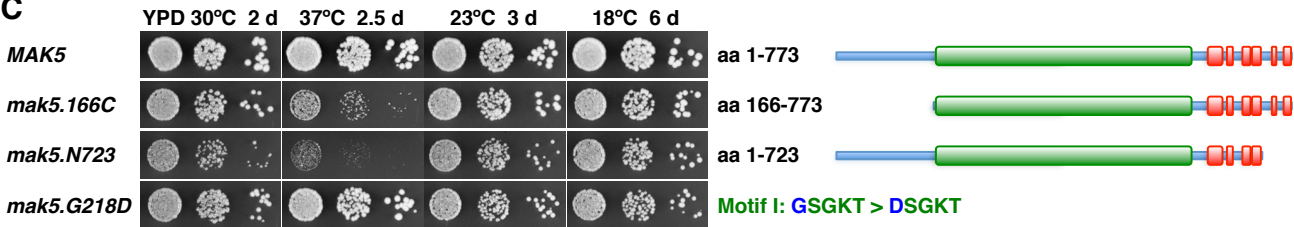

Supplement: Figure S5 — The C-terminal extension to the DEAD-box core of Mak5 harbours an essential function. (A) Multiple sequence alignment, generated in the ClustalW output format with T-Coffee, of the C-terminal extensions of Mak5 from S. cerevisiae, Schizosaccharomyces pombe (Accession: NP_596107) and Homo sapiens (DDX24; Accession: NP_065147). Conserved (*), strongly similar (:) and weakly similar (.) amino acids are indicated below the alignment. The last motif of the DEAD-box core (motif VI, highlighted in green) was used as the conserved starting point for the alignment. The C-terminal end of the DEAD-box core, derived from sequence comparisons and known DEAD-box RNA helicase structures, is indicated by a dashed green line. Secondary structure elements were predicted by PSIPRED; α-helices are highlighted in red and β-strands in blue. Blue arrowheads indicate the C-terminal ends of the different C-terminal deletion constructs. The position of the mak5.R728* mutation is also shown. (B) Growth phenotypes of N- and C-terminal mak5 deletion mutants. Plasmid-borne wild-type MAK5 or the indicated mak5 deletion mutants, all under the control of the authentic promoter, were transformed into the MAK5 shuffle strain. Transformed cells were first restreaked on SC-Leu plates and then spotted in 10-fold serial dilution steps onto SC-Leu and SC+5-FoA plates, which were incubated for 3 d or 4 d at 30ºC. The proteins encoded by the N- and/or C-terminally truncated mak5 mutants are schematically depicted on the right; the DEAD-box core is indicated in green and predicted α-helices within the C-terminal extension are highlighted in red. (C) Growth phenotypes of viable N- and C-terminal deletion mutants and the mak5.G218D mutant. Plasmid-borne wild-type MAK5 or the indicated mak5 mutants, all under the control of the authentic promoter, were transformed into the MAK5 shuffle strain. After plasmid shuffling on plates containing 5-FoA, cells were restreaked on YPD plates and then spotted in 10-fold ser [file pone.0082741.s007.pdf]

Supporting Figure S6

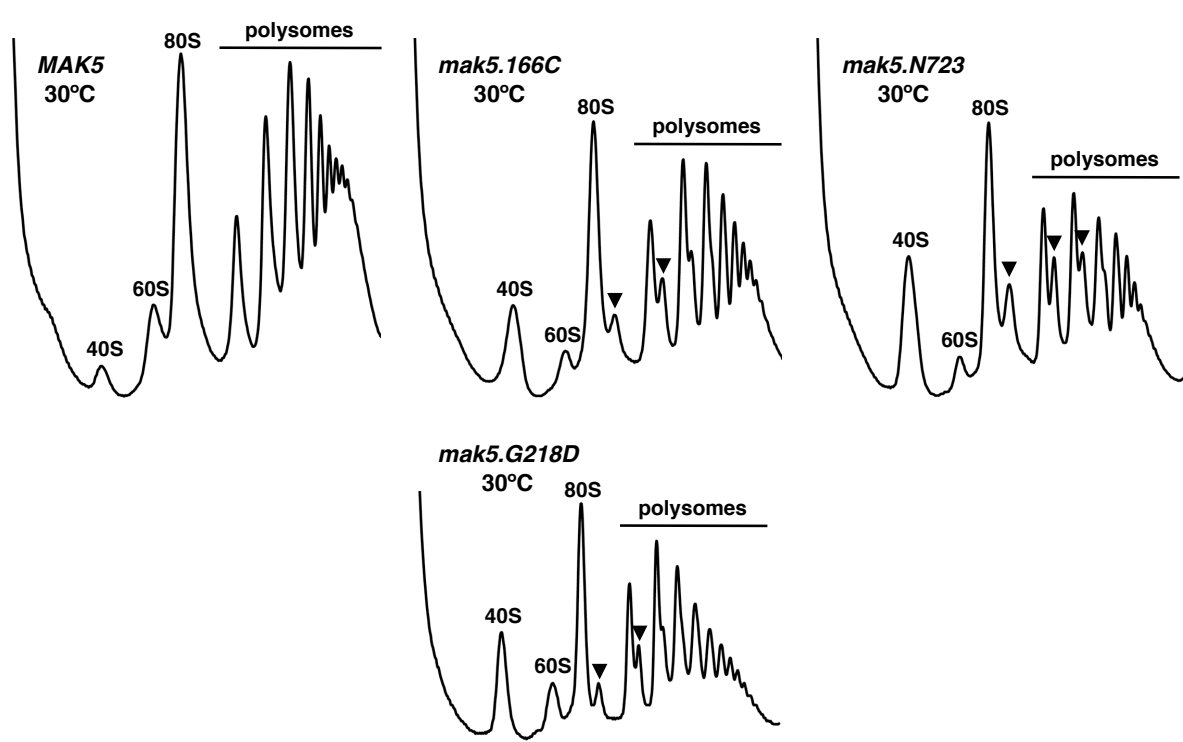

Supplement: Figure S6 — The mak5.166C, mak5.N723 and mak5.G218D mutants exhibit a deficiency in 60S subunit biogenesis. Plasmid-borne wild-type MAK5 or the indicated mak5 mutants, all under the control of the authentic promoter, were transformed into the MAK5 shuffle strain. After plasmid shuffling on plates containing 5-FoA, cells were grown in YPD medium to an OD600 of ~0.8 at 30°C. Cell extracts were prepared under polysome-conserving conditions and eight A260 units were resolved in 10-50% sucrose gradients. The absorption profiles were recorded by continuous monitoring at A254. Sedimentation is from left to right. The peaks of free 40S and 60S subunits, 80S free couples/monosomes and polysomes are indicated. Half-mers are highlighted by arrowheads. (PDF) [file pone.0082741.s008.pdf]

Supporting Figure S9

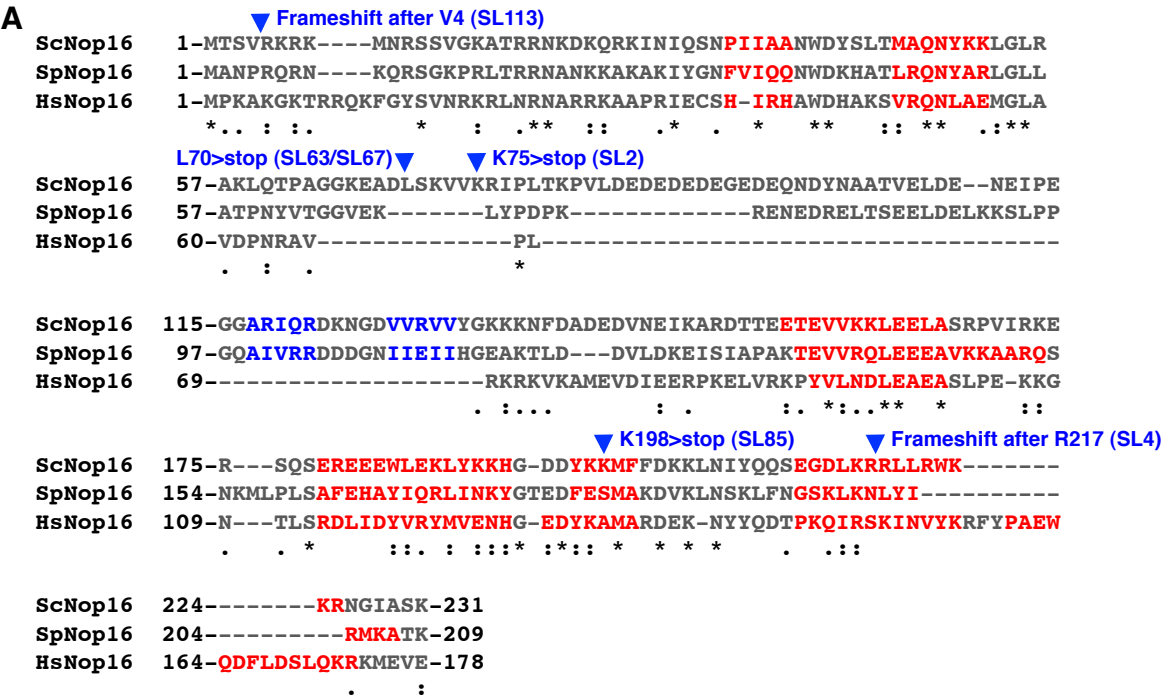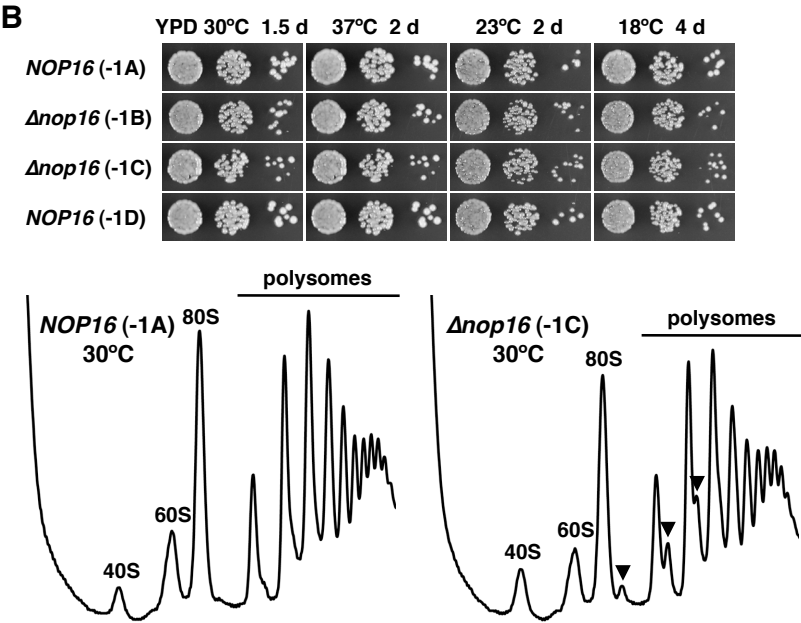

Supplement: Figure S9 — The ∆nop16 null mutant displays only minor growth and 60S biogenesis defects. (A) Multiple sequence alignment, generated in the ClustalW output format with T-Coffee, of Nop16 from S. cerevisiae, S. pombe (Accession: NP_596824) and H. sapiens (hNOP16 isoform 3; Accession: NP_057475). Conserved (*), strongly similar (:) and weakly similar (.) amino acids are indicated below the alignment. Secondary structure elements were predicted by PSIPRED; α-helices are highlighted in red and β-strands in blue. The effects of the nop16 mutations, present in sl-mutant strains SL2, SL4, SL63, SL67, SL85 and SL113, are shown and their positions indicated by blue arrowheads. (B) Growth phenotype (upper part) and polysome profile (lower part) of the ∆nop16 null mutant. The growth of spore clones from a complete tetrad of a heterozygous NOP16/nop16::natNT2 diploid was assessed by spotting cells in 10-fold serial dilution steps onto YPD plates, which were incubated for 1.5 d at 30ºC, 2 d at 37ºC, 2 d at 23ºC and 4 d at 18ºC (upper part). NOP16 and ∆nop16 null mutant cells were grown in YPD medium to an OD600 of ~0.8 at 30ºC. Cell extracts were prepared under polysome-conserving conditions and eight A260 units were resolved in 10-50% sucrose gradients. The absorption profiles were recorded by continuous monitoring at A254 (lower part). Sedimentation is from left to right. The peaks of free 40S and 60S subunits, 80S free couples/monosomes and polysomes are indicated. Half-mers are highlighted by arrowheads. (PDF) [file pone.0082741.s011.pdf]

Supporting Figure S12

A

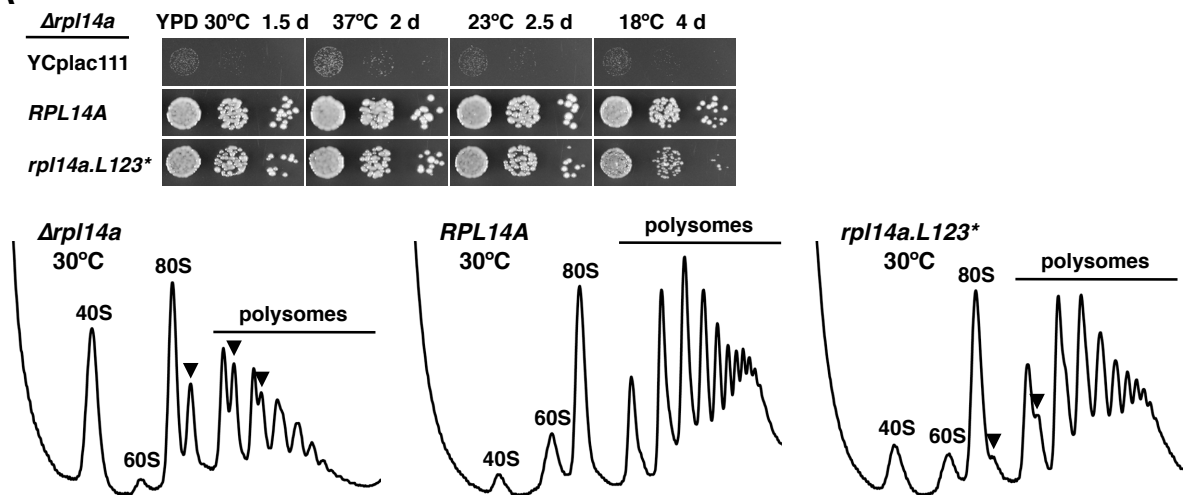

B

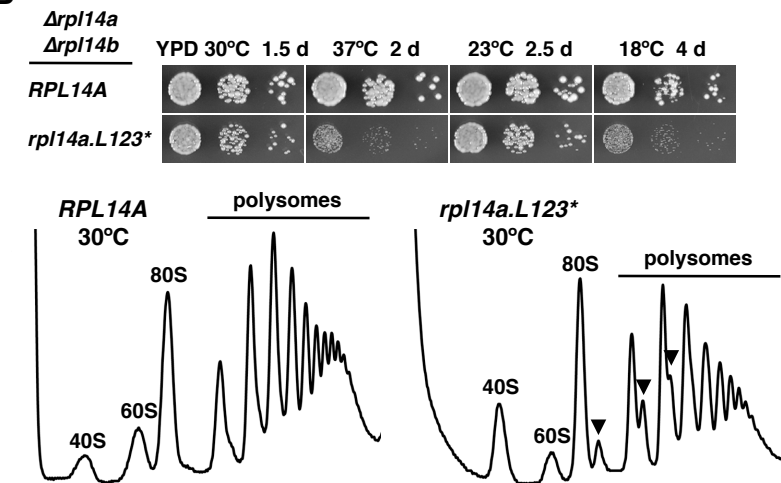

C

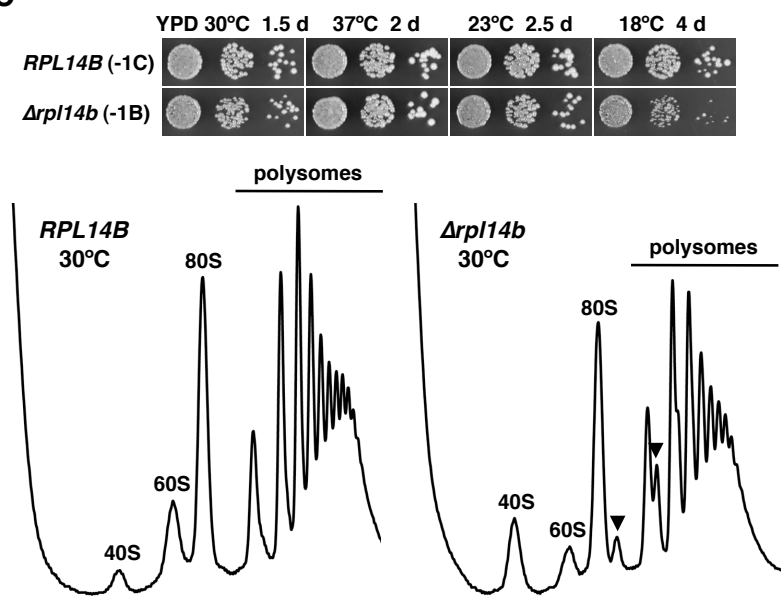

Supplement: Figure S12 — The rpl14a.L123* allele confers a 60S biogenesis defect. Growth phenotype (upper parts) and polysome profile (lower parts) of the rpl14a.L123* mutant in the ∆rpl14a/RPL14B (A) and ∆rpl14a/∆rpl14b (B) background. Empty vector or plasmid-borne wild-type RPL14A and the rpl14a.L123* allele under the control of the authentic promoter were transformed into RPL14A shuffle strains containing the genomic RPL14B copy (A) or carrying a ∆rpl14b null mutation (B). After plasmid shuffling on plates containing 5-FoA, cells were restreaked on YPD plates and then spotted in 10-fold serial dilution steps onto YPD plates, which were incubated for 1.5 d at 30°C, 2 d at 37°C, 2.5 d at 23°C and 4 d at 18°C (upper parts). Cells were grown in YPD medium to an OD600 of ~0.8 at 30°C. Cell extracts were prepared under polysome-conserving conditions and eight A260 units were resolved in 10-50% sucrose gradients. The absorption profiles were recorded by continuous monitoring at A254 (lower parts). Sedimentation is from left to right. The peaks of free 40S and 60S subunits, 80S free couples/monosomes and polysomes are indicated. Half-mers are highlighted by arrowheads. (C) Growth phenotype (upper part) and polysome profile (lower part) of the ∆rpl14b null mutant. The growth of a RPL14B and a ∆rpl14b spore clone, originating from the same complete tetrad of a heterozygous RPL14B/rpl14b::HIS3MX4 diploid, was assessed by spotting cells in 10-fold serial dilution steps onto YPD plates, which were incubated for 1.5 d at 30°C, 2 d at 37°C, 2.5 d at 23°C and 4 d at 18°C (upper part). RPL14B and ∆rpl14b null mutant cells were grown in YPD medium to an OD600 of ~0.8 at 30°C. Preparation of cell extracts as well as analysis and labeling of polysome profiles (lower part) is as described in the legend to Figures S12A and S12B. (PDF) [file pone.0082741.s014.pdf]

Supporting Figure S13

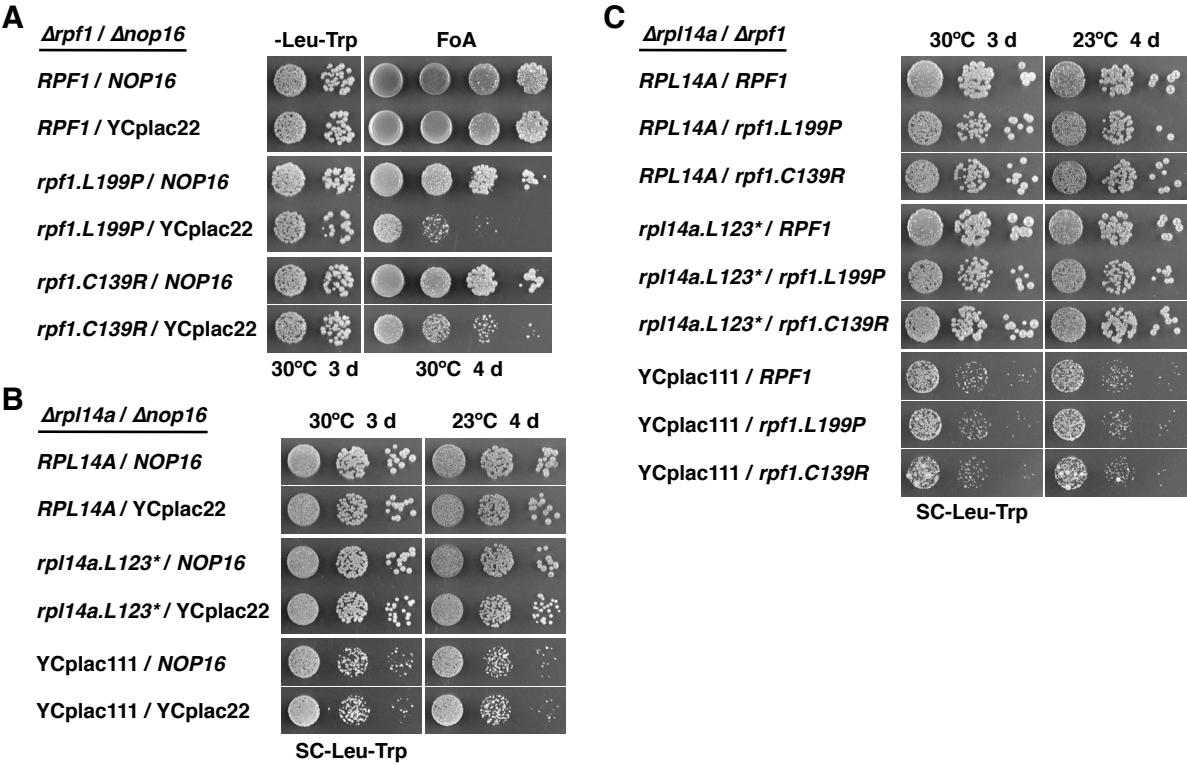

Supplement: Figure S13 — Analysis of synthetic enhancement interactions between rpf1, ∆nop16 and rpl14a.L123* alleles. RPF1/∆nop16 (A), RPL14A/∆nop16 (B) and RPL14A/RPF1 (C) shuffle or double shuffle strains were co-transformed with plasmids harbouring the indicated wild-type and mutant alleles and/or empty vectors (YCplac111 or YCplac22). Cells were restreaked on SC-Leu-Trp plates and then spotted in 10-fold serial dilution steps onto SC-Leu-Trp and SC+5-FoA-Leu-Trp plates, which were incubated for 3 d or 4 d at 30°C (A). In the case of the RPL14A/∆nop16 (B) and RPL14A/RPF1 (C) shuffle or double shuffle strains, transformed cells were restreaked, after plasmid shuffling on plates containing 5-FoA, on SC-Leu-Trp plates and then spotted in 10-fold serial dilution steps onto SC-Leu-Trp plates, which were incubated for 3 d at 30°C or 4 d at 23°C. (PDF) [file pone.0082741.s015.pdf]

Supporting Figure S14

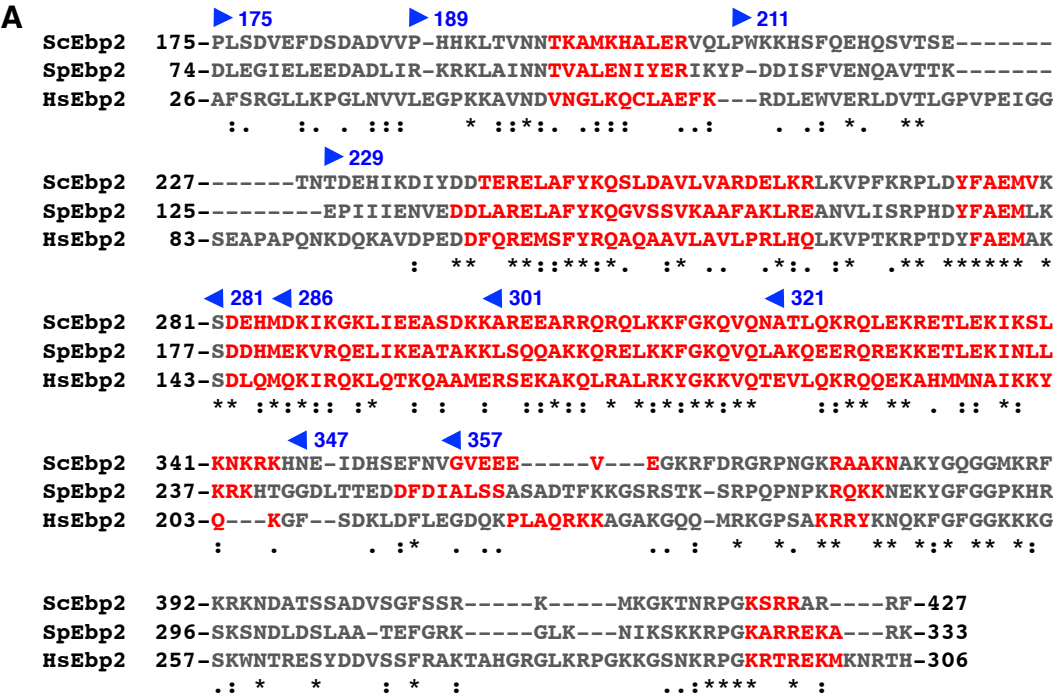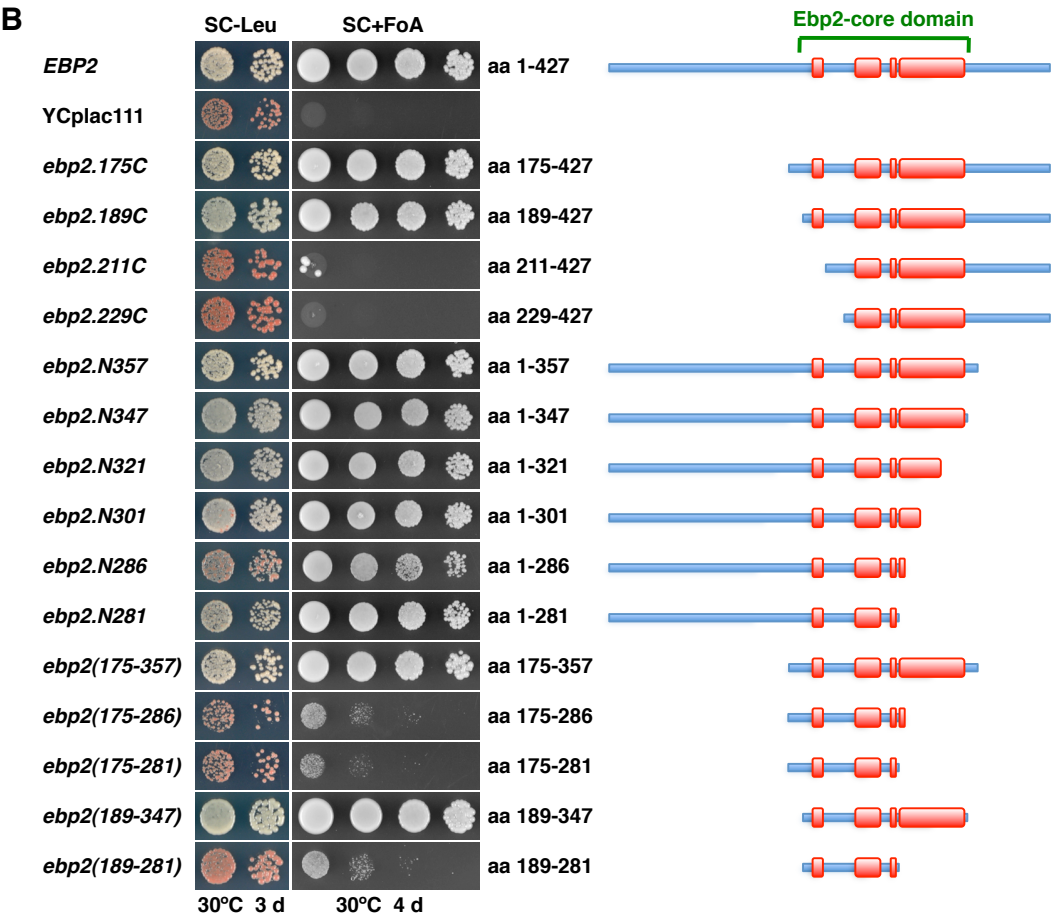

Supplement: Figure S14 — The conserved and α-helical Ebp2-core domain is sufficient to fulfill the essential Ebp2 function. (A) Multiple sequence alignment, generated in the ClustalW output format with T-Coffee, of Ebp2, excluding its weakly conserved N-terminal extension, from S. cerevisiae, S. pombe (Accession: NP_593575) and H. sapiens (hEBP2 isoform 2; Accession: NP_006815). Conserved (*), strongly similar (:) and weakly similar (.) amino acids are indicated below the alignment. Secondary structure elements were predicted by PSIPRED; α-helices are highlighted in red. Blue arrowheads indicate the N- and C-terminal ends of the different N- and/or C-terminal deletion constructs. (B) Growth phenotype of N- and/or C-terminal ebp2 deletion mutants. Empty vector or plasmid-borne wild-type EBP2 and the indicated ebp2 deletion mutants under the control of the authentic promoter were transformed into the EBP2 shuffle strain. Transformed cells were first restreaked on SC-Leu plates and then spotted in 10-fold serial dilution steps onto SC-Leu and SC+5-FoA-Leu plates, which were incubated for 3 d or 4 d at 30ºC. Schematic representations of Ebp2 and the N- and/or C-terminally truncated Ebp2 variants are depicted on the right. For simplicity, only the predicted α-helices, highlighted in red, within the conserved Ebp2-core domain are indicated. (PDF) [file pone.0082741.s016.pdf]

Supporting Figure S15

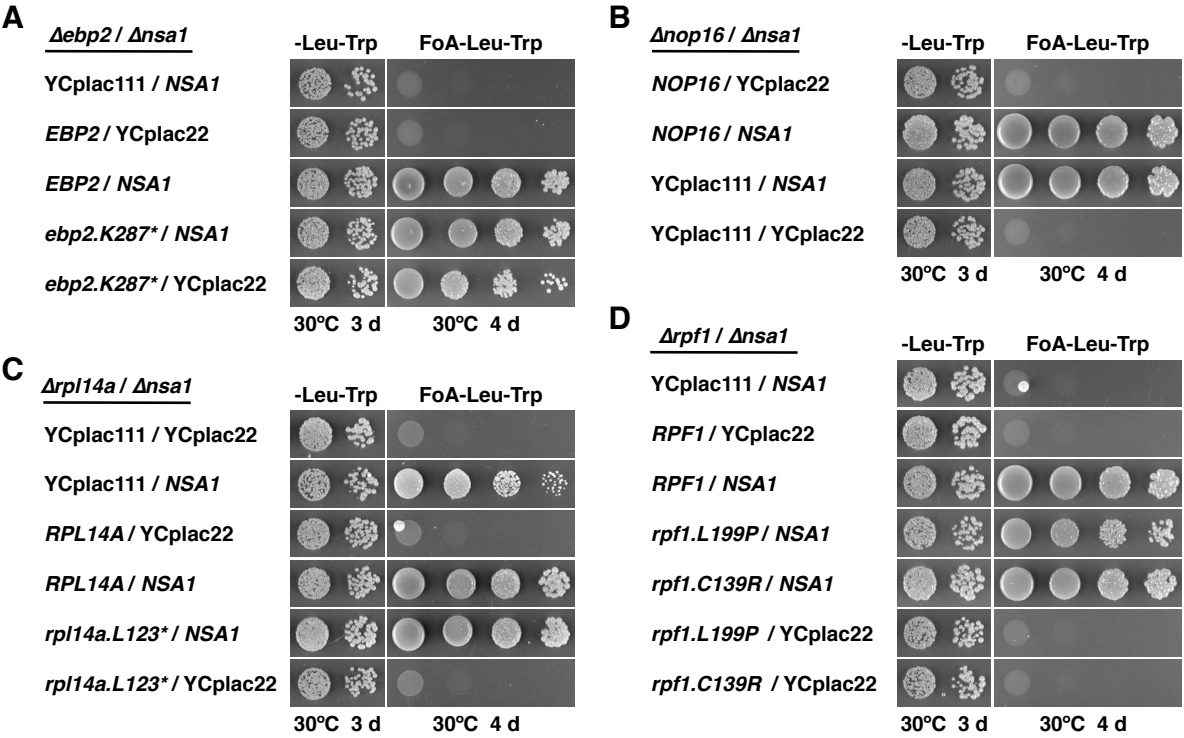

Supplement: Figure S15 — The ebp2.K287* allele suppresses the lethality of ∆nsa1 null mutant cells. EBP2/NSA1 (A), ∆nop16/NSA1 (B), RPL14A/NSA1 (C) and RPF1/NSA1 (D) single and double shuffle strains were co-transformed with plasmids harbouring the indicated wild-type and mutant alleles and/or empty vectors (YCplac111 or YCplac22). Transformed cells were restreaked on SC-Leu-Trp plates and then spotted in 10-fold serial dilution steps onto SC-Leu-Trp and SC+5-FoA-Leu-Trp plates, which were incubated for 3 d at 30°C and 4 d at 30°C, respectively. (PDF) [file pone.0082741.s017.pdf]
