# Supplementary material for: Mak5 and Ebp2 Act Together on Early Pre-60S Particles and Their Reduced Functionality Bypasses the Requirement for the Essential Pre-60S Factor Nsa1
Source: PLoS One. 2013 Dec 2;8(12):e82741. doi: 10.1371/journal.pone.0082741 (PMC3846774; doi:10.1371/journal.pone.0082741)

Supporting Figure S7

A

```
ScEbp2     1-MAKGFKLKELLSHQKEIEKAEKLENDLKKKKSQELKKEEPTIVTASNLKKLEKKEKKADV
SpEbp2     1-MA-GIESKQRRAQKKAAKA------AMKEKKNKES-NESSTSVEALNEKEMINTIK----
HsEbp2     1-M-----------------------------------------------------------
               *

ScEbp2    61-KKEVAADTEEYQSQALSKKEKRKLKKELKKMQEQDATEAQKHMSGDEDESGDDREEEEEE
SpEbp2       ------------------------------------------------------------
HsEbp2       ------------------------------------------------------------

ScEbp2   121-EEEEEGRLDLEKLAKSDSESEDDSESENDSEEDEDVVAKEESEEKEEQEEEQDVPL-SDV
SpEbp2    49---------------SPIIETADTADQENESEGSDE---------------VELSDL-EGI
HsEbp2     2-------------------DTPPLSDSESES--DESLV----------TD-RELQDAFSRG
                                 ::   ::.*.:*  .:.                 :     .

ScEbp2   180-EFDSDAD-VVPHHKLTVNNTKAMKHALERVQLPWKKHSFQEHQSVTSE------------
SpEbp2    79-ELEEDAD-LIRKRKLAINNTVALENIYERIKYP-DDISFVENQAVTTK------------
HsEbp2    31-LLKPGLNVVLEGPKKAVNDVNGLKQCLAEFK---RDLEWVERLDVTLGPVPEIGGSEAPA
               :. . : ::    * ::*:. .:::    ..:     . .: *.   **

ScEbp2   227--TNTDEHIKDIYDDTERELAFYKQSLDAVLVARDELKRLKVPFKRPLDYFAEMVKSDEHM
SpEbp2   125----EPIIIENVEDDLARELAFYKQGVSSVKAAFAKLREANVLISRPHDYFAEMLKSDDHM
HsEbp2    88-PQNKDQKAVDPEDDFQREMSFYRQAQAAVLAVLPRLHQLKVPTKRPTDYFAEMAKSDLQM
                             :  **  **::**:*.  :* ..  .*:. :*  .** ****** *** :*

             K287>stop (SL19)
ScEbp2   286-DKIKGKLIEEASDKKAREEARRQRQLKKFGKQVQNATLQKRQLEKRETLEKIKSLKNKRK
SpEbp2   182-EKVRQELIKEATAKKLSQQAKKQRELKKFGKQVQLAKQEERQREKKETLEKINLLKRKHT
HsEbp2   148-QKIRQKLQTKQAAMERSEKAKQLRALRKYGKKVQTEVLQKRQQEKAHMMNAIKKYQ---K
              :*:: :*  : :  :  ::*:: * *:*:**:**    ::** ** . :: *:  :   .

ScEbp2   346-HNE-IDHSEFNVGVEEE-----VEGKRF--DRGRPNGKRAAKNAKYGQGGMKRFKRKNDA
SpEbp2   242-GGDLTTEDDFDIALSSASADTFKKGSRSTKSRPQPNPKRQKKNEKYGFGGPKHRSKSNDL
HsEbp2   205-GF--SDKLDFLEGDQKPLAQRKKAGAKGQQMRKGPSAKRRYKNQKFGFGGKKKGSKWNTR
                     . :*  . ..         * :    *  *. **   ** *:* ** *: .: *

ScEbp2   398-TSSADVSGFSSR-----K-----MKGKTNRPGKSRRAR----RF-427
SpEbp2   302-DSLAA-TEFGRK-----GLK---NIKSKKRPGKARREKA---RK-333
HsEbp2   263-ESYDDVSSFRAKTAHGRGLKRPGKKGSNKRPGKRTREKMKNRTH-306
               *     : *   :               ..:****  * :
```

B

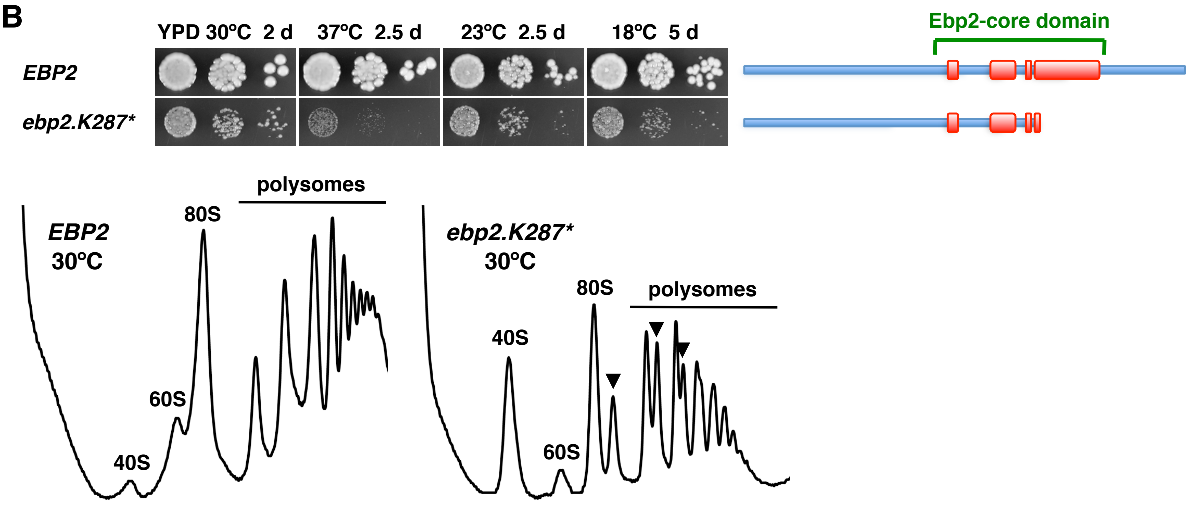

Supplement: Figure S7 — Ebp2 contains a conserved α-helical core domain and ebp2.K287* displays a 60S subunit deficiency. (A) Multiple sequence alignment, generated in the ClustalW output format with T-Coffee, of Ebp2 from S. cerevisiae, S. pombe (Accession: NP_593575) and H. sapiens (hEBP2 isoform 2; Accession: NP_006815). Conserved (*), strongly similar (:) and weakly similar (.) amino acids are indicated below the alignment. Secondary structure elements were predicted by PSIPRED; α-helices are highlighted in red. The position of the ebp2.K287* mutation, which truncates Ebp2 after amino acid 286, present in sl-mutant strain SL19 is indicated by a blue arrowhead. (B) Growth phenotype (upper part) and polysome profile (lower part) of the ebp2.K287* mutant. Plasmid-borne wild-type EBP2 or the ebp2.K287* allele under the control of the authentic promoter were transformed into the EBP2 shuffle strain. After plasmid shuffling on plates containing 5-FoA, cells were restreaked on YPD plates and then spotted in 10-fold serial dilution steps onto YPD plates, which were incubated for 2 d at 30ºC, 2.5 d at 37ºC, 2.5 d at 23ºC and 5 d at 18ºC (upper part). Schematic representations of Ebp2 and the C-terminally truncated Ebp2.K287* protein are depicted on the right of the upper part. For simplicity, only the predicted α-helices, highlighted in red, within the conserved Ebp2-core domain are indicated. EBP2 and ebp2.K287* cells were grown in YPD medium to an OD600 of ~0.8 at 30ºC. Cell extracts were prepared under polysome-conserving conditions and eight A260 units were resolved in 10-50% sucrose gradients. The absorption profiles were recorded by continuous monitoring at A254 (lower part). Sedimentation is from left to right. The peaks of free 40S and 60S subunits, 80S free couples/monosomes and polysomes are indicated. Half-mers are highlighted by arrowheads. (PDF) [file pone.0082741.s009.pdf]
