# Supplementary material for: Mak5 and Ebp2 Act Together on Early Pre-60S Particles and Their Reduced Functionality Bypasses the Requirement for the Essential Pre-60S Factor Nsa1
Source: PLoS One. 2013 Dec 2;8(12):e82741. doi: 10.1371/journal.pone.0082741 (PMC3846774; doi:10.1371/journal.pone.0082741)

Supporting Figure S8

A

```
ScRpf1     1-MALGN------------------------------------------------EINITNK
SpRpf1     1-MG-----------------------------------------------------KIKNK
HsRpf1     1-MAKAGDKSSSSGKKSLKRKAAAEELQEAAGAGDGATENGVQPPKAAAFPPGFSISEIKNK
             *.                                                     :*.**

ScRpf1    13-LKRQEIFADIKHEKNKERHTMRRKRAKEERENPELREQRLKENVTQTIENTRVYDETIN-
SpRpf1     8-IVRQQQYMKALHQKNKDKLERRKERAKEEEKDPEKKRLRLSENIPATIESKRVYDETIIE
HsRpf1    61-QRRHLMFTRWKQQQRKEKLAAKKKLKKEREA---LGDKAPPKPVPKTIDNQRVYDETTVD
                *:  :    ::::.*::    :::   **..            :  :.  **:.  ******

ScRpf1    72-----KEVEGDE--DDLMRYFNSNSNEPPKIFLTTNVNAKKSAYEFANILIEILPNVTFVK
SpRpf1    68-DKPDEELQAELKDDEFSAYFSEE-RKVPKLLVTTSKRASRKCYDFASELLDCFPNAEFRK
HsRpf1   118--PNDEEVAYDEATDEFASYFNK-Q-TSPKILITTSDRPHGRTVRLCEQLSTVIPNSHVYY
                  :*:  :    *::   **..      **:::**. ..        :.. *    :**   .

                               ▼ C139>R (SL33)
ScRpf1   126-RKFGYKLKEISDICIKRNFTDIVIINEDKKKVTGLTFIHLPEGPTFYFKLSSFVEVKKIV
SpRpf1   127-RTGDIEVHEIAEAAAKRGYTDLLVLNEDRKKTNALTLVHLPNGPSFYFTLSNLQTAKEIS
HsRpf1   175-RR-GLALKKIIPQCIARDFTDLIVINEDRKTPNGLILSHLPNGPTAHFKMSSVRLRKEIK
              *   .   :::*    .   *.:**::::***:*.  ..* :  ***:**: :*.:*..    *:*

                                ▼ L199>P (SL3)
ScRpf1   186-GHGR-PTSHIPELILNNFQTRLGQTVGRLFQSILPQNPDIEGRQVITLHNQRDYIFFRRH
SpRpf1   187-NHGR-STGHIPELIINNFSTRLGMTVARAFQSLFIQTPQIQGRQVVTIHCQRDFLFFRRH
HsRpf1   234-RRGKDPTEHIPEIILNNFTTRLGHSIGRMFASLFPHNPQFIGRQVATFHNQRDYIFFRFH
               :*:  .* ****:*:*** **** ::.* * *:: :.*:: **** *:* ***::*** *
                              σ70-like RNA binding motif
ScRpf1   245-RYVFKDNER------VGLQELGPQFTLKLKRLQRGIKE----ETEWEHKP-EMDKEKKKFYL-295
SpRpf1   246-RYAFREKSNMPDGIGTGLQELGPRFTMRLRMVQKGVWDRKEGEVFFESNA-GEESDRRKFWL-306
HsRpf1   294-RYIFRSEKK------VGIQELGPRFTLKLRSLQKGTFDSKYGEYEWVHKPREMDTSRRKFHL-349
             ** *:.:..      .*:*****:**::*: :*:*   :     *   :   :.    :..::** *
```

B

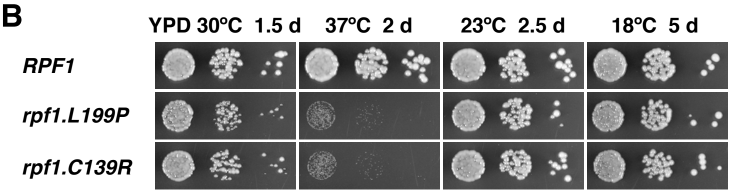

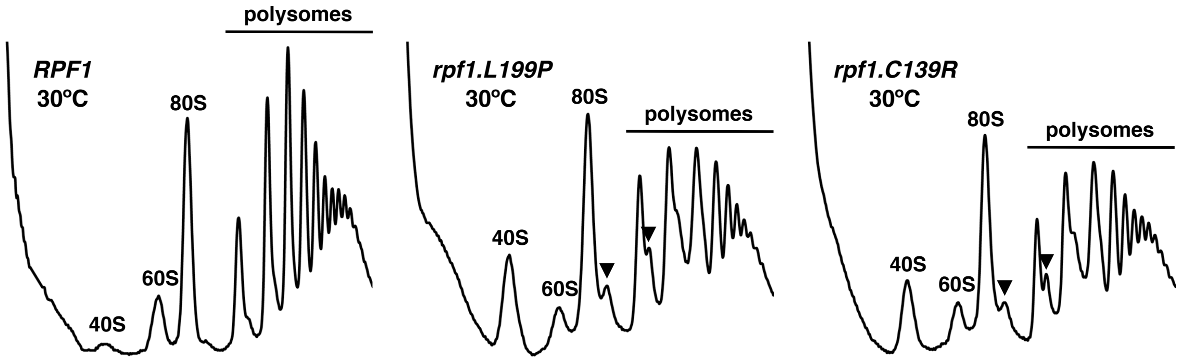

Supplement: Figure S8 — The rpf1.L199P and rpf1.C139R mutants are temperature sensitive and display a 60S biogenesis defect. (A) Multiple sequence alignment, generated in the ClustalW output format with T-Coffee, of Rpf1 from S. cerevisiae, S. pombe (Accession: NP_593868) and H. sapiens (Accession: NP_079341). Conserved (*), strongly similar (:) and weakly similar (.) amino acids are indicated below the alignment. Secondary structure elements were predicted by PSIPRED; α-helices are highlighted in red and β-strands in blue. The positions of the rpf1.L199P and rpf1.C139R mutations, present in sl-mutant strains SL3 and SL33, are indicated by blue arrowheads. The σ70-like RNA binding motif, which is conserved among Brix-family ribosome biogenesis factors, is also indicated. (B) Growth phenotype (upper part) and polysome profile (lower part) of the rpf1.L199P and rpf1.C139R mutants. Plasmid-borne wild-type RPF1 or the rpf1.L199P and rpf1.C139R alleles under the control of the authentic promoter were transformed into the RPF1 shuffle strain. After plasmid shuffling on plates containing 5-FoA, cells were restreaked on YPD plates and then spotted in 10-fold serial dilution steps onto YPD plates, which were incubated for 1.5 d at 30ºC, 2 d at 37ºC, 2.5 d at 23ºC and 5 d at 18ºC (upper part). RPF1, rpf1.L199P and rpf1.C139R cells were grown in YPD medium to an OD600 of ~0.8 at 30ºC. Cell extracts were prepared under polysome-conserving conditions and eight A260 units were resolved in 10-50% sucrose gradients. The absorption profiles were recorded by continuous monitoring at A254 (lower part). Sedimentation is from left to right. The peaks of free 40S and 60S subunits, 80S free couples/monosomes and polysomes are indicated. Half-mers are highlighted by arrowheads. (PDF) [file pone.0082741.s010.pdf]
