# Supplementary material for: Mak5 and Ebp2 Act Together on Early Pre-60S Particles and Their Reduced Functionality Bypasses the Requirement for the Essential Pre-60S Factor Nsa1
Source: PLoS One. 2013 Dec 2;8(12):e82741. doi: 10.1371/journal.pone.0082741 (PMC3846774; doi:10.1371/journal.pone.0082741)

Supporting Figure S10

A

```
                  Rpl9 (L6) binding surface
ScRpl14     1-MSTDSIVKASNWRLVEVGRVVLIKKGQSAGKLAAIVEIIDQKKVLIDGPK
SpRpl14     1-ME-------GFKRYVEVGRVVLVTKGEYTGKLAVIVDIVDHKRALIDSPC
HsRpl14     1-MV--------FRRFVEVGRVAYVSFGPHAGKLVAIVDVIDQNRALVDGPC
              *           * ******. :. *  :***..**:::*:::.*:*.*

                      W303 polymorphism: A89>G ▼
ScRpl14    51-AGVPRQAINLGQVVLTPLTFALPRGARTATVSKKWAAAGVCEKWAASSWA
SpRpl14    44-SEFPRQVIRYGSVVLTHIVMKLPRGARSGIVAKKWKAQDVCNKWASSAWA
HsRpl14    43-TQVRRQAMPFKCMQLTDFILKFPHSAHQKYVRQAWQKADINTKWAATRWA
              : . **.:    : ** : : :*:.*:   * : *   .:  ***:: **

                                    ▼ L123>stop (SL103)
ScRpl14   101-KKIAQRERRAALTDFERFQVMVLRKQKRYTVKKALAKA-138
SpRpl14    94-KKLEAKKVRSQLNDFDRFAVMRLKKQRREQVNVAVAKALKA-134
HsRpl14    93-KKIEARERKAKMTDFDRFKVMKAKKMRNRIIKNEVKKLQKAALLKAS-139-[X]-215
              **:  :: :: :.**:** **  :* :.  ::  : *
```

B

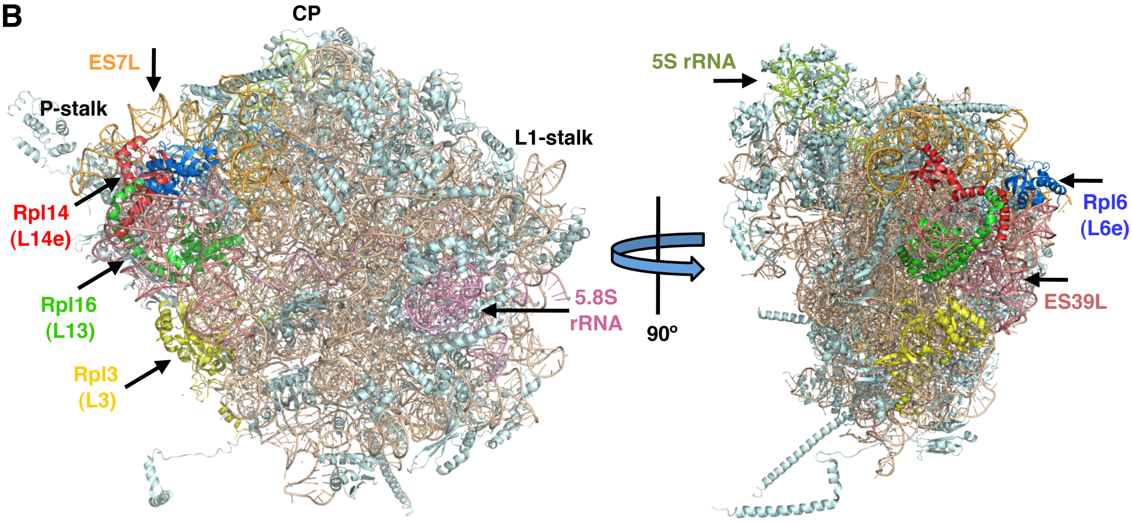

C

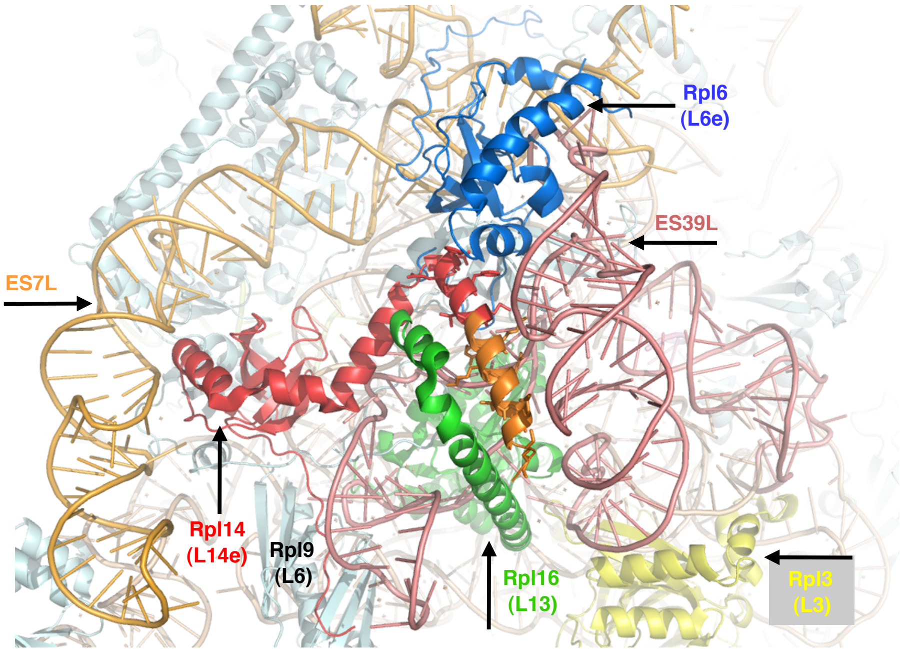

Supplement: Figure S10 — The rpl14a.L123* mutation truncates the C-terminal α-helix, which is part of a eukaryote-specific 60S subunit surface. (A) Multiple sequence alignment, generated in the ClustalW output format with T-Coffee, of Rpl14 (L14e) from S. cerevisiae (Rpl14a; W303 background), S. pombe (Accession: NP_593924) and H. sapiens (Accession: NP_003964). Conserved (*), strongly similar (:) and weakly similar (.) amino acids are indicated below the alignment. Secondary structure elements of Rpl14 were taken from the yeast 60S crystal structure (PDB 3U5H and 3U5I; [56]) and the human 60S cryo-EM structure (PDB 3J3B and 3J3F; [57]) or predicted by PSIPRED; α-helices are highlighted in red and β-strands in blue. The position of the rpl14a.L123* mutation, which truncates Rpl14a after amino acid 122, present in sl-mutant strain SL103 is indicated by a blue arrowhead. The portion of the C-terminal α-helix that is missing in the Rpl14a.L123* mutant protein is coloured in orange. The short N-terminal segment of S. cerevisiae Rpl14, mediating an interaction with Rpl9 (L6) (see panel C), is highlighted in green. (B) Position of Rpl14 and its close neighbours, forming a eukaryote-specific 60S subunit surface, on the S. cerevisiae 60S crystal structure (PDB 3U5H and 3U5I [56]). To facilitate the orientation, the 60S subunit is both shown in its solvent-side (back) view (left part) and when rotated counter-clockwise for 90º along the y-axis (right part); moreover characteristic features of 60S subunits, such as the central protuberance (CP), the P-stalk and the L1-stalk are indicated. The 25S, 5.8S and 5S rRNAs are shown in wheat, pink and lime, respectively. (C) Close-up of the eukaryote-specific 60S subunit surface formed by the r-proteins Rpl6 (L6e), Rpl14 (L14e) and Rpl16 (L13) and the rRNA expansion segments ES7L and ES39L. The r-proteins Rpl6, Rpl14 and Rpl16 are shown in blue, red and green, respectively. The rRNA expansion segments ES7L and ES39L are shown in bright orange and salmon. Mor [file pone.0082741.s012.pdf]
