# Supplementary material for: Mak5 and Ebp2 Act Together on Early Pre-60S Particles and Their Reduced Functionality Bypasses the Requirement for the Essential Pre-60S Factor Nsa1
Source: PLoS One. 2013 Dec 2;8(12):e82741. doi: 10.1371/journal.pone.0082741 (PMC3846774; doi:10.1371/journal.pone.0082741)
Supplement: Figure S11 — Rpl14 and Rpl16 contain strictly eukaryote-specific C-terminal α-helices. (A) Multiple sequence alignment, generated in the ClustalW output format with T-Coffee, of Rpl14 (L14e) from S. cerevisiae (Rpl14a; W303 background) and L14 from the archaeal organisms Sulfolobus solfataricus (Accession: YP_005643206) and Methanocaldococcus jannaschii (Accession: NP_247641). Conserved (*), strongly similar (:) and weakly similar (.) amino acids are indicated below the alignment. Secondary structure elements of Rpl14 were taken from the yeast 60S crystal structure (PDB 3U5H and 3U5I; [56]) or predicted by PSIPRED; α-helices are highlighted in red and β-strands in blue. The exclusively eukaryote-specific C-terminal α-helices of Rpl14 are indicated. The portion of the C-terminal α-helix that is missing in the Rpl14a.L123* mutant protein is coloured in orange. Note that L14 is not present in Haloarcula marismortui. (B) Multiple sequence alignment, generated in the ClustalW output format with T-Coffee, of Rpl16 (L13) from S. cerevisiae (Rpl16b) and L13 from the archaeal organisms S. solfataricus (Accession: NP_341641), M. jannaschii (Accession: NP_247162) and H. marismortui (Accession: YP_134851). Conserved (*), strongly similar (:) and weakly similar (.) amino acids are indicated below the alignment. Secondary structure elements of Rpl16 and L13 were taken from the yeast 60S crystal structure (PDB 3U5H and 3U5I; [56]) and the H. marismortui 50S crystal structure (PDB 1VQO; [98]), respectively, or predicted by PSIPRED; α-helices are highlighted in red and β-strands in blue. The exclusively eukaryote-specific C-terminal α-helix of Rpl16 is indicated. (PDF) [file pone.0082741.s013.pdf]

Supporting Figure S11

A

```
ScRpl14     1-MSTDSIVKASNWRLVEVGRVVLIKKGQSAGKLAAIVEIIDQKKVLIDGPK
SsL14       1-MP-----------AIEVGRICVKVKGREAGSKCVIVDIIDDNFVLVTGPK
MjL14       1-MP-----------AIEVGRVCIKTAGREAGKVCVIVDILDKNFVIVDGL-
              *.            :****: :   *:.**. ..**:*:*.: *:: *

ScRpl14    51---AGVPRQAINLGQVVLTPLTFALPRGARTATVSKKWAAAGVCEKWAASS
SsL14      40-DITGVKRRRVNILHLEPTDKKIDIQKGASDEEVKKKLEESNLTEYMKEKI
MjL14      39-----VKRRRCNIKHLEPTEKKVDIPKGASTEEVKLALDAAGLLKEE-----80
                  * *:  *: ::  *  .. : :**    *.     :.: :
                   eukaryote-specific C-terminal α-helices
ScRpl14    99-WAKKIAQRERRAALTDFERFQVMVLRKQKRYTVKKALAKA-138
SsL14      90-KIRMPTL-96
MjL14
```

B

```
ScRpl16b    1-MSQ----PVVVIDAKDHLLGRLASTIAKQVLNGQKIVVVRAEALNISGEF
SsL13       1-MSTQVQEQVVIINAEGQILGRMASNVVRLLKEGKKVIIVNGEKAVISGEK
MjL13       1-M--------TVIDAEGAILGRLASEVAKRVLRGEEIVIVNAEMVVITGNK
HmL13       1-MSVAEFDADVIVDARDCIMGRVASQVAEQALDGETVAVVNAERAVITGRE
              *        .:::*.. ::**:** :..    *: : :*..*   *:*.

ScRpl16b   47-FRNKLKYHDFLRKATAFNK-TRGPFHFRAPSRILYKAIRGMVSHKTARGK
SsL13      51-NRVIESYKLLLTVKTLFNPYRNGIRRPRSPINIVKRTIRGMLP-KSSKGR
MjL13      43-DWIIKTYQEEREKKNVANPRRFGPKFPRRPDDILRRTIRKMLPYKKPKGR
HmL13      51-EQIVEKYEKRVD---IGN--DNGYFYPKRPDGIFKRTIRGMLPHKKQRGR
                   .*.         *    *    : *  *. ::** *:. *. :*:

ScRpl16b   96-AALERLKIFEGIPPPYDKKKRVVVPQALRVLRLKPGRKYTTLGKLSTSVG
SsL13     100-RMLKNVKIYVGVPKEFEGRQFIKFPDS-DVSRLK--GKYVTVEVVSKELG
MjL13      93-EAFKRVKVYVGNPKNLTV------DEK-ISHKLN-TTKYITLAELSKHLG
HmL13      96-EAFESVRVYLGNPYDEDGE--VL-DGT-SLDRLS-NIKFVTLGEISETLG
                :: :::: * *                  :*.   *: *:  :*  :*
                          eukaryote-specific C-terminal α-helix
ScRpl16b  146-WKYEDVVAKLEDKRKVRSAEYYAKKRAFTKKVSSASAAASESDVAKQLASFGY-198
SsL13     147-WSG--------------------------------------------------149
MjL13     135-AKF--------------------------------------------------137
HmL13     141-ANKTW------------------------------------------------145
```
